# Dose imbalance of DYRK1A kinase causes systemic progeroid status in Down syndrome by increasing the un-repaired DNA damage and reducing LaminB1 levels

**DOI:** 10.1016/j.ebiom.2023.104692

**Published:** 2023-07-12

**Authors:** Aoife Murray, Gillian Gough, Ana Cindrić, Frano Vučković, David Koschut, Vincenzo Borelli, Dražen J. Petrović, Ana Bekavac, Ante Plećaš, Valentina Hribljan, Reinhard Brunmeir, Julija Jurić, Maja Pučić-Baković, Anita Slana, Helena Deriš, Azra Frkatović, Jűrgen Groet, Niamh L. O’Brien, Hong Yu Chen, Yee Jie Yeap, Frederic Delom, Steven Havlicek, Luke Gammon, Sarah Hamburg, Carla Startin, Hana D’Souza, Dinko Mitrečić, Mijana Kero, Ljubica Odak, Božo Krušlin, Željka Krsnik, Ivica Kostović, Jia Nee Foo, Yuin-Han Loh, Norris Ray Dunn, Susana de la Luna, Tim Spector, Ingeborg Barišić, Michael S.C. Thomas, Andre Strydom, Claudio Franceschi, Gordan Lauc, Jasminka Krištić, Ivan Alić, Dean Nižetić

**Affiliations:** a Faculty of Medicine and Dentistry, Blizard Institute, Queen Mary University of London, London, UK; b The London Down Syndrome Consortium (LonDownS), London, UK; c Lee Kong Chian School of Medicine, Nanyang Technological University, Singapore; d Glycoscience Research Laboratory, Genos Ltd., Zagreb, Croatia; e Disease Intervention Technology Laboratory (DITL), Institute of Molecular and Cellular Biology (IMCB), Agency for Science, Technology and Research (A∗STAR), Singapore; f Department of Medical and Surgical Sciences, Alma Mater Studiorum University of Bologna, Italy; g Croatian Institute for Brain Research, School of Medicine, University of Zagreb, Zagreb, Croatia; h Faculty of Veterinary Medicine, Department of Anatomy, Histology and Embryology, University of Zagreb, Zagreb, Croatia; i Institute of Molecular and Cell Biology (IMCB), A∗STAR, Singapore; j Laboratory of Neurogenetics, Genome Institute of Singapore, A∗STAR, Singapore; k Department of Forensic and Neurodevelopmental Sciences, Institute of Psychiatry, Psychology and Neuroscience, King's College London, London, UK; l Division of Psychiatry, University College London, London, UK; m School of Psychology, University of Roehampton, London, UK; n Centre for Brain and Cognitive Development, Birkbeck, University of London, London, UK; o Department of Medical Genetics, Children’s Hospital Zagreb, Centre of Excellence for Reproductive and Regenerative Medicine, School of Medicine, University of Zagreb, Zagreb, Croatia; p Department of Pathology, School of Medicine, University of Zagreb, Zagreb, Croatia; q ICREA, Genome Biology Programme (CRG), Universitat Pompeu Fabra (UPF), CIBER of Rare Diseases, Barcelona, Spain; r Department of Twin Research and Genetic Epidemiology, King's College London, London, UK; s Institute of Information Technologies, Mathematics and Mechanics, Lobachevsky State University, Nizhny Novgorod 603022, Russia; t Faculty of Pharmacy and Biochemistry, University of Zagreb, Zagreb, Croatia

**Keywords:** Down syndrome, Down syndrome critical region, Chromosome 21, Ageing, DYRK1A, DYRK1A inhibitors, LaminB1, IgG glycan

## Abstract

**Background:**

People with Down syndrome (DS) show clinical signs of accelerated ageing. Causative mechanisms remain unknown and hypotheses range from the (essentially untreatable) amplified-chromosomal-instability explanation, to potential actions of individual supernumerary chromosome-21 genes. The latter explanation could open a route to therapeutic amelioration if the specific over-acting genes could be identified and their action toned-down.

**Methods:**

Biological age was estimated through patterns of sugar molecules attached to plasma immunoglobulin-G (IgG-glycans, an established “biological-ageing-clock”) in n = 246 individuals with DS from three European populations, clinically characterised for the presence of co-morbidities, and compared to n = 256 age-, sex- and demography-matched healthy controls. Isogenic human induced pluripotent stem cell (hiPSCs) models of full and partial trisomy-21 with CRISPR-Cas9 gene editing and two kinase inhibitors were studied prior and after differentiation to cerebral organoids.

**Findings:**

Biological age in adults with DS is (on average) 18.4–19.1 years older than in chronological-age-matched controls independent of co-morbidities, and this shift remains constant throughout lifespan. Changes are detectable from early childhood, and do not require a supernumerary chromosome, but are seen in segmental duplication of only 31 genes, along with increased DNA damage and decreased levels of LaminB1 in nucleated blood cells. We demonstrate that these cell-autonomous phenotypes can be gene-dose-modelled and pharmacologically corrected in hiPSCs and derived cerebral organoids. Using isogenic hiPSC models we show that chromosome-21 gene *DYRK1A* overdose is sufficient and necessary to cause excess unrepaired DNA damage.

**Interpretation:**

Explanation of hitherto observed accelerated ageing in DS as a developmental progeroid syndrome driven by DYRK1A overdose provides a target for early pharmacological preventative intervention strategies.

**Funding:**

Main funding came from the “Research Cooperability” Program of the 10.13039/501100004488Croatian Science Foundation funded by the 10.13039/501100000780European Union from the 10.13039/501100004895European Social Fund under the Operational Programme Efficient Human Resources 2014–2020, Project PZS-2019-02-4277, and the Wellcome Trust Grants 098330/Z/12/Z and 217199/Z/19/Z (UK). All other funding is described in details in the “Acknowledgements”.

**Research in context**

**Evidence before this study**

Primary DS foetal neurons grown *in vitro* show increased reactive oxygen species (ROS) production, malfunctioning mitochondria, and an increased tendency to apoptosis. A recent study during neuronal differentiation from T21 iPSCs found that at the neural progenitor cell stage, T21 causes a major upheaval of epigenetic organisation of the nucleus, similar to the epigenetic changes in oxidative-stress-induced-senescence. This study did not find significant changes at the pluripotent undifferentiated iPSC stage, nor measured DNA damage. However, signs of increased DNA damage, and/or malfunctioning DNA-damage-repair (DDR) were seen in other bodily systems: primary DS gingival cells, fibroblasts and lymphocytes. Also recently, accelerated immune system ageing was reported in DS (n = 26 individuals), using cytometry-based immunophenotyping, with the T-cell compartment being the most affected. Evidence for accelerated ageing defined by the DNA-methylation-based “epigenetic clock” has been recorded for some cell types in DS. It remains unanswered whether accelerated biological ageing in such diverse DS tissues and cell types is caused by the actions of different, tissue and cell-type specific overdosed chr21 genes via different mechanisms, or by an over-arching mechanism driving DS cells of multiple tissues into progeroid state starting very early in development.

**Added value of this study**

Our study adds a unifying mechanism (cell-autonomous excess unrepaired DNA damage driving a laminopathy-associated progeroid status) and points to a single dose-sensitive chr21 gene causing it (*DYRK1A*), that so far had not been directly linked to accelerated ageing. It represents a comprehensive assessment of the degree of systemic biological ageing in DS. We calculate that the average biological age of adults with DS corresponds to that of 18.4–19.1 years older healthy control individuals. We show that T21 alone, in the absence of co-morbidities (Alzheimer’s dementia, thyroid disease, autoimmune diseases or frequent infections), can drive this phenotype. We show that the slope of biological ageing curves (the rate of age-dependent IgG-glycan changes) between DS and euploid adults remains the same throughout lifetime, providing direct evidence against the “amplified genomic instability concept” which would predict a progressively amplifying ageing curve. We show that trisomic dose of just 31 chr21 genes (13% of chr21 gene content, without a freely segregating supernumerary chromosome) is sufficient to increase IgG-glycan ageing marks in the serum and cause increased DNA double-strand breaks (DSB) with reduced Lamin B1 protein in peripheral blood mononuclear cells. We show that these effects of T21 can be modelled in undifferentiated isogenic iPSCs, and assign these abnormalities to a specific chromosomal region, and a specific gene, *DYRK1A*. We verify this by chemical inhibition of DYRK1A kinase activity, and overexpression of wild-type or kinase-dead mutant of DYRK1A in full chromosome T21 iPSCs in which *DYRK1A* has been knocked out by gene editing. We further demonstrate that hypo-expression of Lamin B1 seen in iPSCs is also observed in some foetal and infant DS tissues, including liver, skin fibroblasts and brain. Using cerebral organoids, we demonstrate that reduced cortical folding (a shared phenotype of DS and *LMNB1*^+/−^ patients) can be restored by CRISPR-Cas9 reduction of *DYRK1A* copy number, and that neuronal DNA damage, senescence and Lamin B1 depletion in early DS organoids can be corrected by pharmacological inhibition of DYRK1A.

**Implications of all the available evidence**

Recently, clinical trials using the DYRK1A inhibitor epigallocatechin gallate (EGCG) have been conducted in young adults with DS. Our data suggests that inhibition of DYRK1A-overdose effects, if applied to earlier developmental stages, might ameliorate important neurodevelopmental issues associated with DS. This concept should be approached carefully, as constitutional DYRK1A haploinsufficiency causes microcephaly. It therefore remains a challenge to find modalities of fine-tuning this inhibition, in order to ameliorate, and not worsen, the neurodevelopmental consequences in DS. The list of DS neurodevelopmental phenotypes that could be counter-acted by the early therapeutic restoration of Lamin B1 levels can best be estimated by comparing the pathological phenotypes between individuals with DS, *LMNB1*^+/−^ patients, and *Lmnb1*^−/−^ mice, and it includes: impaired neuronal migration, reduced neuron numbers at specific brain locations, gliogenic shift during brain development, reductions in gyral folding, dendritic spines and synaptic puncta. Mature T21 neurons show additional, neuron-specific toxic insults that destabilise ROS and accelerate DNA damage, potentially necessitating also the inhibition of other chr21 gene products (such as Aβ peptides) at later stages of brain development.

## Introduction

Down syndrome (DS) is an aneuploid condition caused by full or partial trisomy 21 (T21).^1^^,^^2^ Besides characteristic features resulting from facial, skeletal, muscular and soft-tissue changes, it is the most common genetic cause of intellectual disability, early-onset Alzheimer’s disease (AD) and dementia.^3^ In addition to AD, signs of ageing-related reduction in tissue regenerative capacity (such as alopecia, xerosis, delayed wound healing, chronic periodontitis, osteoporosis and immunosenescence) are often seen in DS earlier than in age-matched euploid individuals.^4^, ^5^, ^6^, ^7^ While increased incidence and earlier onset of AD in DS is clearly caused by the triplication of the chromosome 21 (chr21) gene for amyloid precursor protein (APP),^8^^,^^9^ the explanations for all other ageing-related phenomena are less well understood.^10^^,^^11^ Signs of increased DNA damage and/or hypo-functioning DNA-damage-repair (DDR) mechanisms were seen in DS neural progenitor cells (NPCs) and neurons,^12^, ^13^, ^14^ fibroblasts,^15^, ^16^, ^17^ lymphocytes^18^, ^19^, ^20^, ^21^ and gingival cells.^6^^,^^22^ Evidence for accelerated ageing defined by the DNA-methylation-based “epigenetic clock” has been recorded for some cell types in DS,^23^ but the extent to which this affects the other aspects of biological ageing and its relationship to co-morbidities of DS remains unclear.

We recently established a “non-epigenetic-clock” for both chronological and biological ageing using Immunoglobulin-G (IgG) glycosylation as a biomarker.^24^^,^^25^ A study of 5117 individuals from four European populations revealed very extensive and complex changes in IgG glycosylation with age. The combined index composed of only three IgG glycan traits (one glycan without galactose and two glycans with two galactoses) explained up to 64% of variance in age, considerably more than other biomarkers of age, including telomere lengths. The remaining variance in these glycans strongly correlated with physiological parameters associated with general health status.^24^

With the aim of measuring DS population ageing and its relationship with co-morbidities using this non-epigenetic clock, we performed a systematic analysis of IgG glycosylation patterns in three cohorts of adults with DS that were also characterised for the most common co-morbidities: Alzheimer’s dementia, thyroid dysfunction, other autoimmune diseases, and frequent respiratory tract infections. The comparison with IgG glycosylation profiles of healthy euploid individuals matched for age and sex was performed on each DS cohort as a whole, and separately for the sub-cohorts with, and those without, specific co-morbidities. We also measured IgG-glycan age profiles in 38 children with DS compared to 11 euploid children of a similar age range. Surprisingly, a 2-year-old child with DS, but without any supernumerary chromosome, showed IgG-glycan ageing profiles indistinguishable from full T21 DS children, and outside the euploid children’s profile range. We show that a duplicated segment on one of their chromosomes-21 encompassing only 13% of chromosomal gene content is sufficient to cause accelerated IgG-glycan ageing changes seen in DS cohorts, accompanied by an increased presence of DNA double-strand break (DSB)-repair foci and decreased Lamin B1 levels in blood cell nuclei. We further show that these cellular phenotypes can be modelled in undifferentiated human induced pluripotent stem cells (hiPSCs) and derived cerebral organoids, and identify that trisomic dose and functional activity of one gene in this duplicated segment, *DYRK1A*, is necessary to cause the accelerated cellular ageing.

## Methods

### Human samples

This study was based on banked plasma samples obtained from three European cohorts of persons with DS:

A) DS cohort from France

Plasma samples from 98 adult individuals with DS aged 30–67 years (median 46 years) were provided by CRB-BioJeL. DS samples in the French cohort were stratified for the presence or absence of dementia, autoimmune diseases (type of autoimmune disease was also specified) and frequent infection. An age- and sex-matched control group of plasma samples from 109 healthy individuals aged 22–67 years (median 46 years) was selected from the Split cohort which contains samples from individuals from the Croatian city of Split collected through the “10,001 Dalmatians” project.^26^^,^^27^

B) DS cohort from Italy

Plasma samples from 57 adult individuals with DS aged 22–66 years (median 36 years) from the Italian DS cohort were used in this study. Individuals with DS from the Italian cohort were stratified for the presence or absence and type of autoimmune diseases and frequent infection. Plasma samples from 53 healthy adult individuals aged 22–66 years (median 38 years) of Italian ethnicity, selected from banked samples collected through the “PainOmics” project,^28^ served as control samples. In addition, plasma samples from 35 individuals with DS (ages 10–58 years, median age 26 years) and 35 of their healthy siblings (ages 9–52 years, median age 31 years) were obtained from the Italian DS cohort and were analysed in this study. These sibling pairs were recruited in the Emilia-Romagna region (Bologna and Ferrara provinces) in Italy.

C) DS cohort from the UK

Plasma samples from 53 adult individuals with DS aged 22–73 years (median 49 years) from the London Down Syndrome Consortium (LonDownS) cohort^29^ were used in this study. DS samples in the UK cohort were stratified for the presence or absence of dementia, autoimmune diseases (type of autoimmune disease was also specified) and frequent infection. Control plasma samples were selected from the TwinsUK cohort which is the UK’s largest adult twin registry and contains over 14,000 twins.^30^ In total, samples from 42 individuals aged 22–82 years (median age 47 years) from the TwinsUK cohort were included in this study. Individuals from the TwinsUK cohort have been shown to have comparable disease-related, lifestyle and anthropomorphic characteristics to those of age-matched individuals from the general UK population.^31^ In addition, 38 plasma samples from children with DS with age ranging from 0.58 years to 5.25 years from the LonDownS Consortium cohort were analysed in this study. Among these were eight samples obtained from around 4-year-old DS children (three boys and five girls, ages 3.58–4.17 years). Plasma samples from 17 healthy control children who were from 3 to 5 years old were provided by Children’s Hospital Srebrnjak (CHS) in Zagreb, Croatia. These control children samples were collected through the “ATOPICA” project at CHS, as previously described.^32^ Among these were 11 samples obtained from healthy 4-year-old children (three boys and eight girls).

D) CRO1 partial trisomy

The sample CRO1 was obtained from a 2-year-old child, having observed the clinical features of DS. After finding a normal number of chromosomes, high-resolution banding cytogenetic analysis, followed by genomic DNA analysis using CGH array, confirmed the presence of a small segmental duplication in 21q22. This duplication was fine-mapped by a SNP array on an Illumina OmniExpress v1.1 chip. Analysis was performed and figures were generated using GenomeStudio 2.0 software. Following the list of features reported for other partial trisomy 21 cases,^33^ the clinical observations were subsequently followed up in greater detail (summarised in Fig. 9).

### Ethics

This study was performed in accordance with the Helsinki declaration. Written informed consent was obtained from all individual participants included in the study or from the participant’s parents or guardians. Ethical approvals were obtained by relevant ethics committees: for DS cohort from the CRB-BioJeL in Paris, France approval was obtained from the Ministry of Higher Education, Research and Innovation for biobanking activities (AC-2015-2579), and for human samples exportation (IE-2015-814); for Italian cohort of Down syndrome ethical approval was obtained from the local Ethical Committee (S. Orsola Hospital, University of Bologna); for LonDowns cohort ethical approval was obtained from the North West Wales National Health Service (NHS) Research Ethics Committee (13/WA/0194); “10,001 Dalmatians” study was approved by Ethical Board of the Medical School, University of Split, Croatia; “PainOmics” study was approved by Ethical Committee of University of Parma (UNIPR), Italy and Fondazione IRCCS Policlinico San Matteo Hospital (OSM), Italy; the TwinsUK study was approved by Westminster Research Ethics Committee; “ATOPICA” study was approved by Children’s Hospital Srebrnjak (CHS) Ethics Committee; for the partial trisomy 21 child, the study was approved by the Ethical Research Committee of the Children’s Hospital Zagreb (University of Zagreb, School of Medicine). To ensure a blinded study, the plasma samples were coded by number or by combination of letters and numbers.

### Experimental design: randomisation, blocking and used standards

The cohort size was based on the maximum sample number we could assemble from clinically characterised individuals with DS in the UK, France and Italy. Plasma samples from DS individuals and healthy individuals which served as controls were randomised across seven 96-well collection plates. To ensure that each of the seven plates had the same age distribution, sex ratio and ratio of persons with DS and controls as the entire collection of samples and also to ensure an approximately equal number of individuals from each individual cohort on each plate, blocking was performed. In addition to plasma samples from individuals with DS and healthy controls, each plate contained 3–5 wells loaded with human plasma which served as a standard and was obtained from the Croatian National Institute of Transfusion Medicine. One well on each plate contained no plasma and served as a negative control sample. The randomisation and blocking methods used in this study are described more precisely in.^34^

### Immunoglobulin G (IgG) isolation

Plasma samples were vortexed after thawing and centrifuged at 12,100*g* for 3 min or 5000*g* for 10 min. Then, 100 μL of each plasma sample was aliquoted to 1 mL 96-well collection plates (Waters, Milford, MA, USA) following a predetermined experimental design described above. Plasma samples were diluted with 700 μL of PBS, pH 7.4, and filtered through a 0.45 μm GHP filter plate (Pall Corporation, Ann Arbor, MI, USA). IgG was isolated from plasma samples by affinity chromatography using 96-well monolithic plates with bound Protein G (BIA Separations, Ajdovščina, Slovenia) as described previously.^25^ Following IgG isolation, IgG concentrations were measured at 280 nm using a NanoDrop spectrophotometer (NanoDrop 8000, Thermo Scientific, USA).

### IgG N-glycan release, labelling and clean-up

The whole procedure was performed as described previously.^35^ Briefly, 300 μL of IgG eluates were dried in a vacuum centrifuge. After drying, IgG was denatured with sodium dodecyl sulphate (SDS) (Invitrogen, Carlsbad, CA, USA) and a 10 min incubation at 65 °C. The excess of SDS was neutralised with Igepal-CA630 (Sigma-Aldrich, St. Louis, MO, USA). N-glycans were released from IgG by overnight digestion with PNGase F (Promega, Madison, WI, USA). The released IgG N-glycans were labelled with 2-aminobenzamide (2-AB, Sigma-Aldrich, St. Louis, MO, USA). Free label and reducing agent were removed from the samples using hydrophilic interaction liquid chromatography solid-phase extraction (HILIC-SPE) on a 0.2 μm GHP filter plate (Pall Corporation, Ann Arbor, MI, USA). IgG N-glycans were eluted with ultrapure water and stored at −20 °C until use.

### Ultra-high-performance liquid chromatography (UHPLC)

Fluorescently labelled N-glycans were separated by hydrophilic interaction chromatography (HILIC) on a Waters Acquity UPLC instrument (Milford, MA, USA) consisting of a quaternary solvent manager, sample manager and a FLR fluorescence detector set with excitation and emission wavelengths of 250 and 428 nm, respectively. The instrument was under the control of Empower 3 software, build 3471 (Waters, Milford, MA, USA). Labelled N-glycans were separated on a Waters BEH Glycan chromatography column, 100 × 2.1 mm i.d., 1.7 μm BEH particles, with 100 mM ammonium formate, pH 4.4, as solvent A and ACN as solvent B. The separation method used a linear gradient of 75%–62% ACN (v/v) at flow rate of 0.4 mL/min over 27 min. Samples were maintained at 10 °C before injection and the separation temperature was 60 °C. The system was calibrated using an external standard of hydrolysed and 2-AB-labelled glucose oligomers from which the retention times for the individual glycans were converted to glucose units (GU). Data processing was performed using an automatic processing method with a traditional integration algorithm, after which each chromatogram was manually corrected to maintain the same intervals of integration for all the samples. All chromatograms were separated in the same manner into 24 peaks (GP1–GP24) as previously reported.^35^ The amount of glycans in each peak was expressed as a percentage of total integrated area (% area). To confirm that glycan structures found in each of the 24 peaks are those reported by,^25^ GU values of each peak were compared to the reference values in the “GlycoStore” database available at https://glycostore.org/. All glycan structures were further confirmed with exoglycosidase digestions. The following enzymes, all purchased from New England Biolabs (NEB, Ipswich, MA, USA) were used for digestions: α2-3,6,8,9 Neuraminidase A, β1-4 Galactosidase S, β1-3 Galactosidase, β-N-Acetylglucosaminidase S, α1-2,4,6 Fucosidase O. Aliquots of the 2-AB labelled glycan pool were dried down and digested according to the manufacturer's protocol. After overnight incubation at 37 °C, enzymes were removed by filtration through AcroPrep 96 Filter Plates, 10K (Pall Corporation, Ann Arbor, MI, USA). Digested glycans were then separated by HILIC-UHPLC for comparison against undigested glycans.

### Statistical analysis for IgG-glycan studies

To remove experimental variation from measurements, normalisation and batch correction were performed on UHPLC glycan data. To make measurements across samples comparable, normalisation by total area was performed where peak area of each of 24 glycan structures was divided by total area of the corresponding chromatogram. Prior to batch correction, normalised glycan measurements were log transformed due to right-skewing of their distributions and multiplicative nature of batch effects. Batch correction was performed on log-transformed measurements using the ComBat method (R package sva), where technical source of variation (which sample was analysed on which plate) was modelled as batch covariate. To get measurements corrected for experimental noise, estimated batch effects were subtracted from log-transformed measurements. Glycan peaks 20 and 21 (GP20 and GP21) were not well separated in UHPLC glycan profiles of samples from the fourth and fifth plates. Therefore, these two peaks were excluded from statistical analysis and derived trait calculations. In addition to 22 directly measured IgG glycans (glycan peaks), six derived traits were calculated from the directly measured glycans. These derived traits average glycosylation features across different individual glycan structures and are consequently more closely related to individual enzymatic activities and underlying genetic polymorphisms. Formulas used for the calculation of derived IgG glycan traits were as follows: IgG glycans without galactose G0 total = GP1 + GP2 + GP3 + GP4 + GP6; IgG glycans with one galactose G1 total = GP7 + GP8 + GP9 + GP10 + GP11; IgG glycans with two galactoses G2 total = GP12 + GP13 + GP14 + GP15, IgG glycans with sialic acid(s) S total = GP16 + GP17 + GP18 + GP19 + GP22 + GP23 + GP24; IgG glycans with core fucose F total = GP1 + GP4 + GP6 + GP8 + GP9 + GP10 + GP11 + GP14 + GP15 + GP16 + GP18 + GP19 + GP23 + GP24; IgG glycans with bisecting GlcNAc B total = GP3 + GP6 + GP10 + GP11 + GP13 + GP15 + GP19 + GP22 + GP24.

Differences in N-glycosylation of IgG between individuals with DS and healthy controls were analysed using a/the general linear model. Age and sex variables were included in the model to control for their effects. A/The general linear model was also used to determine whether associations exist between IgG N-glycome and various clinical variables (e.g., autoimmunity, dementia, etc.) within the DS group. Differences in IgG N-glycome between individuals with DS and their siblings were analysed using a/the linear mixed effects model where family ID was included in a model as a random intercept, with age and sex included as additional covariates. Analyses were firstly performed for each cohort separately and then combined using a fixed effects meta-analysis approach (R package meta, metagen (method = “FE”)). Prior to analyses, glycan variables were all transformed to standard Normal distribution (mean = 0, sd = 1) by inverse transformation of ranks to Normality (R package "GenABEL", function rntransform). Using rank transformed variables in analyses makes estimated effects of different glycans in different cohorts comparable as transformed glycan variables have the same standardised variance. False discovery rate was controlled using the Benjamini–Hochberg procedure (function p.adjust (method = “BH”)). Data was analysed and visualised using R programming language (version 3.5.2). Differences in N-glycosylation between children with DS, including CRO1, and healthy children were visualised using principal components analysis (PCA). PCA was applied on directly measured IgG glycan peaks (GP1–GP24) using GraphPad Prism v9.2.0 PCA with standardised scale. Input was all individual GP1–GP24 values, unbiased. PCA was also applied on five derived glycan traits (G0, G1, G2, S and F) the levels of which were found to be significantly different between persons with DS and healthy controls in a large, combined adult cohort.

### Foetal fibroblasts

Four control and three DS primary foetal fibroblast lines were obtained from Galliera tissue bank as detailed in Supplementary Table S1. The cells were gestational-age and passage number matched for experiments. Fibroblasts were cultured in Hams’s F10 with 20% FBS, 2 mM l-Glutamine and 1× penicillin/streptomycin at 37 °C, 5% CO_2_, and passaged with 0.05% trypsin. For immunostaining, glass coverslips were coated with 0.1% gelatin before seeding cells.

### Primary PBMCs and immunostaining

During standard clinical procedure, blood samples of CRO1 child as well as a typically developing euploid, age and sex matched child were collected. From consented material surplus to clinical procedure, peripheral blood mononuclear cells (PBMCs) were isolated by laying blood onto twice the volume of Lymphoprep. The sample was centrifuged at 800×*g* for 30 min, with no brake, to avoid any disruption of blood layers upon stopping. The upper layer of plasma was removed and stored at −80 °C and the next layer of PBMCs was transferred to a fresh tube, washed with 5 mL PBS by centrifuging at 241×*g* for 5 min with standard braking. For immunostaining, freshly isolated PBMCs were cytospun onto glass slides and air-dried for 90 min. Air-dried cells were fixed with 4% paraformaldehyde (PFA) for 10 min, rinsed with distilled water and stored till staining. Immunostaining was performed as described below. Critical step for the staining of those cells was to perform antigen retrieval with 0.01 M citrate buffer (pH 6.0) before blocking/permeabilisation.

### iPSC generation, validation and culture

#### Erythroblast differentiation and Sendai reprogramming

CRO1 refers to a young female individual with DS caused by a microduplication of a 4.083 Mb region overlapping the DSCR. Patient blood sample was collected, as described above, during routine diagnostic tests, and consented surplus material was used to generate the iPSCs.

Extracted PBMCs were cultured in Erythroblast differentiation medium (StemSpan Serum-Free Expansion Medium II (SFEM II; StemCell Technologies) supplemented with: 2 U/mL Human Erythropoietin, 50 μg/mL l-ascorbic acid, 50 ng/mL Stem Cell Factor, 40 ng/mL IGF-1, 10 ng/mL IL-3, 0.4 ng/mL Dexamethasone, 1% l-glutamine and 1% MEM Non-Essential Amino Acids) for 10 days to allow growth and expansion of erythroblasts. Erythroblasts were reprogrammed using the iPS 2.0 Sendai Reprogramming Kit comprising three reprogramming vectors containing the four Yamanaka factors: KOS (human Klf4 and human Oct3/4, Sox2), hc-Myc (human c-Myc) and hKlf4 (human Klf4) (CytoTune2.0, Invitrogen, Thermo Fisher). Individual colonies were expanded and two clones were selected for experimental use after pluripotency and genome validation.

#### Germ layer differentiation for pluripotency validation

iPSCs were dissociated to single cells, PBS rinsed and counted before being resuspended at 9000 cells per 150 μL HESC media (DMEM F12, 20% KnockOut Serum Replacement, 3% ES-quality foetal bovine serum, 1% GlutaMAX supplement, 1% MEM-Non-Essential Amino Acids and 50 μM 2-mercaptoethanol supplemented with 4 ng/mL βFGF and 50 μM RI). The cell suspension was plated in a 96-well round-bottomed ultra-low attachment plate at 150 μL per well. The plate was centrifuged at 200×*g* for 5 min to aid embryoid body (EB) formation and incubated at 37 °C for three days. On day three, 50% spent media was replaced with fresh HESC media (no βFGF or RI). The EBs were fed bi-weekly for a total of seven days to allow self-directed differentiation into cells of the three embryonic germ layers (endoderm, mesoderm and ectoderm). On day seven, each EB was transferred to a geltrex-coated coverslips in HESC media. The EBs were fed bi-weekly for a further seven days, until the EB attached to the coverslip and the cells began to migrate out. After a total of 14 days in culture (7 days suspension plus 7 days adherent culture), coverslips were fixed for immunofluorescence analysis of the three germ layers.

#### Alkaline phosphatase staining

iPSCs were stained with Alkaline Phosphatase (Merk) according to the manufacturer’s instructions. Briefly, PBS-rinsed fixed iPSCs were incubated with 2:1:1 ratio of FRV: Naphthol As-B1: H_2_O for 15 min at room temperature in the dark. Cells were then rinsed once in PBS them imaged by brightfield microscopy.

#### SNP arrays

Genomic DNA was isolated from iPSCs using standard column kits. DNA of all iPSC lines shown in the manuscript were analysed by SNP array (Illumina OmniExpress v1.1 chips) and analysis performed in Genome Studio 2.0 software. Following CRISPR editing, edited clones were further analysed by SNP array to validate the genomic integrity compared to the parental line used for genome editing. Data for chromosome 21 is presented in the appropriate figures. The rest are not shown for the lack of space, but the data are available upon reasonable request.

The isogenic iPSC model was (NIZEDSM1iD21-C3 -C7 -C9, NIZEDSM1iT21-C5 -C6 -C13), described and characterised in a previous report.^14^

#### Mycoplasma testing

All cells used were confirmed to be mycoplasma free by testing with the EZ-PCR Mycoplasma Detection Kit (Biological Industries).

#### FISH

Chromosome 21 disomic and trisomic status was revalidated in the isogenic NIZEDSM1iD21/NIZEDSM1iT21 model by Fluorescence In-Situ Hybridisation with the XA 13/18/21 Probe (D-5607-100-TC, MetaSystems Probes).

#### Cell lines

Cell lines will be made freely available for non-commercial use, subject to institutional MTAs. Upon publication we aim to deposit the original cell lines generated as part of this study with the UK Stem Cell Bank (https://www.nibsc.org/ukstemcellbank).

### CRISPR/Cas9 genome editing

The guide-RNA (gRNA) [5′-ACTGGAGAACCTCTGTTCAG-3′] targeting *DYRK1A* exon7 was cloned into a vector containing the high fidelity SpCas9-HF1^36^ and blasticidin S resistance gene. The complete plasmid was delivered via Lipofectamine3000 to a trisomic iPSC clone T21C5 (full official name NIZEDSM1iT21-C5), which was described and characterised in a previous report,^14^ and also to CRO1 iPSCs (described in this work).

Untransfected iPSCs were removed by treatment with blasticidin (2 μg/mL for 48 h). Individual colonies were picked and further sub-cloned by limiting dilution to achieve clonal cell lines. DNA was purified from individual clones, PCR amplified and sequenced by Sanger Sequencing. Primer sequences and genome locations are listed in Supplementary Table S2. Sequences were analysed in Mutation Surveyor (V3.1.0) and “Tracking InDels by dEcomposition (TIDE)” (TIDE V2.0.1, Desktop Genetics). TIDE analysis of the CRISPR-targeted clones DNA sequence gave scores which gave an indication if one, two or three alleles were targeted, based on the % of wild-type read remaining.

The quality of the gRNA was assessed using two different prediction software platforms: CCTop online software,^37^ and the MIT online platform (http://crispr.mit.edu/). The same two software platforms were used to predict the off-target sites. Neither platform found any off-targets with 0, 1, or 2 mismatches. The top 10 CCTop-predicted sites were PCR amplified in all targeted clones, then sequenced by Sanger Sequencing (using primers listed in Supplementary Table S2) to rule out off-target events.

#### Clonal isolation of iPSCs

Colonies with a mutation as indicated by Sanger sequencing were harvested by incubation with Accutase (Life Technologies) for 5–10 min until a single cell suspension was achieved. The cells were counted and plated at 500 cells/cm^2^ in a 6-wellplate for clonal cell expansion. Individual colonies were picked into 12 well Matrigel or Vitronectin coated plates approximately 14 days after seeding and expanded for DNA isolation, cryopreservation, and maintenance in culture.

### PCR, DNA sequencing and sequence analysis

5 μL of a 25 μL PCR product was used for gel electrophoresis (1.5% TAE/Agarose gel) and the remaining 20 μL was purified (FavorPrep, Biotech Corp.). The purified PCR products were either analysed with a T7 endonuclease assay (for gRNA efficiency tests) or sent for Sanger sequencing (for identification of insertions or deletions (indels) in targeted iPSCs). Forward primers used for PCR were used for one directional sequencing of PCR products.

Sequence files were analysed in Mutation Surveyor V3.1.0 or uploaded to the Tracking of InDels by Sequence Trace Decomposition (TIDE) webtool for analysis. TIDE, developed by^38^ uses a decomposition algorithm on a pair of sequences uploaded by the user (edited and unedited) to identify major induced mutations at a CRISPR-SpCas9 target site. The software accurately determines the frequency of mutations in the cell population. For example, a heterozygous mutation in disomic cells would be displayed as a sequence trace with 50% normal and 50% mutated sequence. For trisomic cells, 33.3% would be expected per allele.

### DYRK1A inhibitor treatment

For treatment with DYRK1A inhibitors, 48 h after plating, iPSCs were treated for a period of 12 h with harmine (300 nM) or ID8 (10 μM or 500 nM) or an equivalent volume of DMSO as a vehicle only control. Cells were then fixed and stained as standard.

### Cerebral organoid culture and fixation

Cerebral organoids (CO) were generated as previously described.^39^ For treatment with DYRK1A inhibitors, organoids were allowed to form and cultured as normal until DIV30. From this point onwards harmine (300 nM) or ID8 (10 μM or 500 nM) was included in the media at each media change until the organoids were harvested at DIV50 or DIV70. An equivalent volume of DMSO was added to media as a vehicle only control.

### Cerebral organoid size and folding

Average total organoid surface area and cortical folding were quantified on bright-field images using ImageJ software of n = 7–12 CO images per cell line from two separate CO batches. 24 COs were generated from each of the three cell lines per batch and a panel of CO images were captured at the same time point for both batches. The surface area of COs from the CRISPR cell lines were compared to those of the parental CRO1 cell line at three time points: day 3, which represents EBs prior to neural induction, day 10, which represents neuro-EBs after 4 days of neural induction and day 14, which represent COs during neural expansion and differentiation. The organoid cortical folds were manually traced using Adobe Illustrator to quantify fold differences between the CRISPR clones and the parental CRO1 cell line. For each organoid image, the percentage of black pixels (representing rosette folds) vs the percentage of white pixels, were quantified per 10,000 cm^2^.

### Transient transfection with Lipofectamine

pLV-DYRK1A-IRES-GFP plasmids expressing human *DYRK1A* or a K179R kinase dead mutant of human *DYRK1A* have been described.^40^ Two days after plating, iPSCs were transfected with 2.5 μg plasmid per well of a 6-well plate, using Lipofectamine3000, following manufacturer’s protocol.

Approximately 72 h post-transfection cells were fixed in 4% PFA for 15 min at room temperature and rinsed with PBS before immunostaining and imaging as detailed below.

### RNA extraction, cDNA synthesis, and qPCR

RNA was extracted using the Total RNA Miniprep Kit from Monarch following manufacturer’s protocol. cDNA was synthesised with equal concentrations of RNA using the High Capacity cDNA Reverse Transcription Kit from Applied Biosystems following the kit protocol. qPCR was performed using PowerTrack™ SYBR™ Green Master Mix from Applied Biosytems, following manufacturer’s protocol, and using the primers listed in Supplementary Table S3. Fold change was calculated using the ΔΔCT method.

### Western blotting

For western blots, whole-cell lysates of iPSCs or foetal tissues were separated in a 10% acrylamide gel by SDS-PAGE and transferred to a nitrocellulose membrane according to the manufacturers protocols (Bio-Rad). Following a 60 min incubation in 5% non-fat milk in TBS-T the membrane was incubated with primary and secondary antibodies (Supplementary Tables S4 and S5). Quantification was carried out using BioRad software, strictly using the same membranes re-stained using the antibodies shown. Lamin B1 signal was normalised to corresponding β-actin or GAPDH loading control for all samples.

### Histone purification

For the histone purification 2 × 6 well plate/per genotype/condition were used. Cells were resuspended in 1 mL of Hypotonic Lysis Buffer and Eppendorf tubes were rotated at 4 °C for 30 min. Intact nuclei pellet was centrifuged at 10,000×*g*, 4 °C for 10 min. Nuclei were then re-suspended in 400 μL 0.4 N H_2_SO_4_ with Protease Inhibitor Complex and 5 mM Sodium butyrate and rotated one more time at 4 °C for following 30 min. To remove nuclear debris samples were centrifuged at 16,000×*g*, 4 °C for 10 min and supernatants containing the histones were transferred to the new tubes. Histones were precipitated by adding 132 μL Trichloroacetic acid (Fisher Scientific, BP555-250) dropwise and incubated on ice for 30 min. Precipitated histones were centrifuged at 16,000×*g*, 4 °C for 10 min, followed by two washes with cold acetone and centrifugation at 16,000*g*, 4 °C for 5 min. Samples were then air dried at room temperature for 20 min. Finally, extracted histones were re-suspended in 100 μL H_2_O and BCA assay was performed.

### Histology and immunostaining

Unless otherwise stated cells/tissues were fixed with 4% PFA.

Human anonymised post-mortem brain samples were obtained from the Brain Bank of the Croatian Institute for Brain Research (CIBR) while the PFA-fixed, paraffin-embedded liver samples were obtained from Department of Pathology, School of Medicine, University of Zagreb (Supplementary Table S6). Immunostaining was performed as previously described.^39^ Briefly, paraffin embedded human tissues were deparaffinised, rehydrated and antigen retrieval with 0.01 M citrate buffer (pH 6.0) was performed. Following antigen retrieval, slides were incubated in blocking/permeabilisation solution (0.2% Triton X-100 in PBS + 3% donkey serum) for 1 h at room temperature. The slides were incubated over night at 4 °C with primary antibodies (Supplementary Table S4) in solution (0.2% Triton X-100 in PBS + 1% donkey serum). Next day primary antibodies were rinsed 3 × 5 min in PBS and incubated for 2 h with secondary antibodies (Supplementary Table S5) in 0.2% Triton X-100 in PBS at room temperature and rinsed 3 × 5 min in PBS. Nuclei were counterstained with DAPI for 10 min, rinsed 3 × 5 min in PBS and mounted with Dako Fluorescent Mounting Medium. As negative controls for all antibodies, secondary antibody only controls were carried out. For the bright immunostaining instead fluorescent secondary antibodies “VECTASTAIN ABC HRP Kit” was used.

Immunostaining of PFA fixed cerebral organoids and iPSCs followed the same protocol, without deparaffinisation and antigen retrieval. For the staining with mouse Lamin B1 antibody, iPSCs were fixed with ice-cold Methanol at 4 °C for 10 min. Following fixation, the same protocol for immunostaining was performed. Figures were captured on following confocal microscopes: LSM800 Inverted Confocal Microscope with Airyscan (ZEISS) [Singapore], LSM880 Inverted Confocal Microscope with Airyscan (ZEISS) [London], Olympus FV 3000 [Zagreb] and with NanoZoomer 2.0RS (HAMAMATSU) slide scanner.

### Image analysis with Imaris

Image analysis was performed using IMARIS ×64-v9.1.2. Software (BITPLANE, An Oxford Instruments Co., Zurich, Switzerland) (Singapore, London and Zagreb). Quantification was performed blinded to the genotype, using “surface” and “spot” option of IMARIS software. For quantification of Lamin B1, total fluorescence intensity of positive signals for Lamin B1 was normalised to the total fluorescence intensity for DAPI as a nuclear dye in iPSCs or to the total fluorescence intensity for MAP2 as a pan-neuronal marker. Foci, stained with γH2AX, RNF169 and 53BP1 were analysed using “spot” analysis. For each figure, in the first step a DAPI surface was created, using software threshold, to count and label every single nucleus. In the next step spots were labelled and threshold was automatically calculated by software. Finally, spots were counted and analysed on the masked DAPI channel. The percentage of p21-positive cells were also counted using the “spot” option; p21 positive cells were labelled as a spot and normalised with DAPI+ nuclei.

### Comet assay kit (3-well slides) ab238544

Comet Assay on CRO1 iPSCs was performed according to the manufacturer instructions. In brief, Lysis buffer, Alkaline solution and Electrophoresis running buffer (TBE) were stored at 4 °C while the Comet Agarose was heated in water bath at 37 °C. In total, 75 μL heated Comet Agarose per well was added onto the Comet slide to create a Base Layer. Adherent iPSCs colonies were rinsed with PBS and incubated with Accutase. Single cells were re-suspended at 1 × 10^5^ cells/mL in ice-cold PBS. The cells were combined with Comet Agarose at 1/10 ratio and transferred onto the top of the Comet Agarose Base Layer and maintained horizontally at 4 °C in the dark for 15 min. Slides were transferred to the Lysis buffer at 4 °C in the dark for 45 min. Lysis buffer was replaced with pre-chilled Alkaline Solution at 4 °C in the dark for 30 min. Alkaline Solution was replaced with pre-chilled TBE buffer and electrophoresis was performed at 15 V for 15 min. Slides were rinsed 3 × 2 min with pre-chilled dH_2_O and the last water rise was replaced with 70% Ethanol for 5 min. Finally, air dried slides were stained with 100 μL/well with diluted Vista Green DNA Dye at room temperature for 15 min. Fluorescence was captured on EVOS FL Auto System (Thermo Fisher Scientific) fluorescent microscope using FITC filter. Figures were analysed with the Open Comet plug-in of ImageJ.^41^ In our study we have analysed and showed “Olive Moment” which represent the product of tail DNA% and the distance between the intensity-weighted centroids of head and tail.

### Statistics

For cellular experiments (other than the glycan experiments) statistical analyses were carried out using standard procedures and guidelines in GraphPad Prism Software (v.9.2.0). Specific tests used are noted in the figure legend for each set of data. When comparing a single pair of samples, a Student’s T-test was used. For comparisons of more than one sample, one-way ANOVA was carried out, followed by Dunnett’s (if comparing to a control sample) or Tukey’s (if comparing all samples to each other) tests. Unless otherwise stated, statistical significance was defined as p > 0.05 (ns), p < 0.05 (∗), p < 0.01 (∗∗), p < 0.001 (∗∗∗), p < 0.0001 (∗∗∗∗).

### Chemicals and reagents

General chemicals used are listed in Supplementary Table S7.

### Role of funders

The funders had no role in the study design, collection, data analysis, interpretation of writing of the report.

## Results

### Down syndrome populations from three different countries show similar significant alterations in IgG-glycan profiles

**Supplementary Figure S1:**
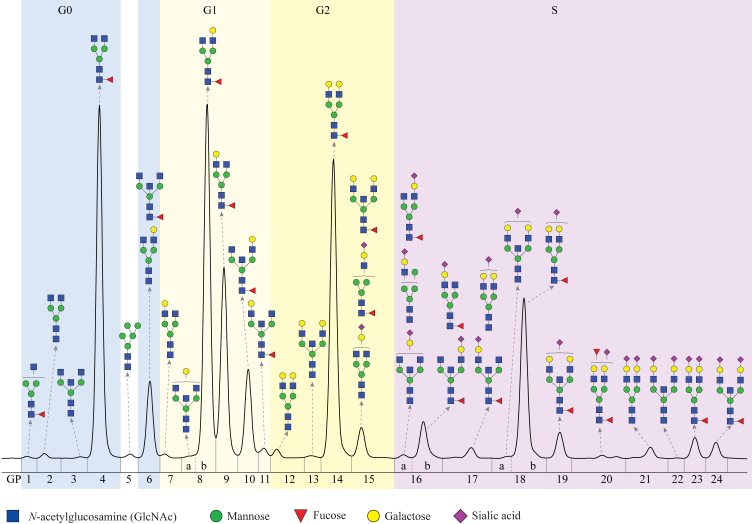


**Supplementary Figure S2:**
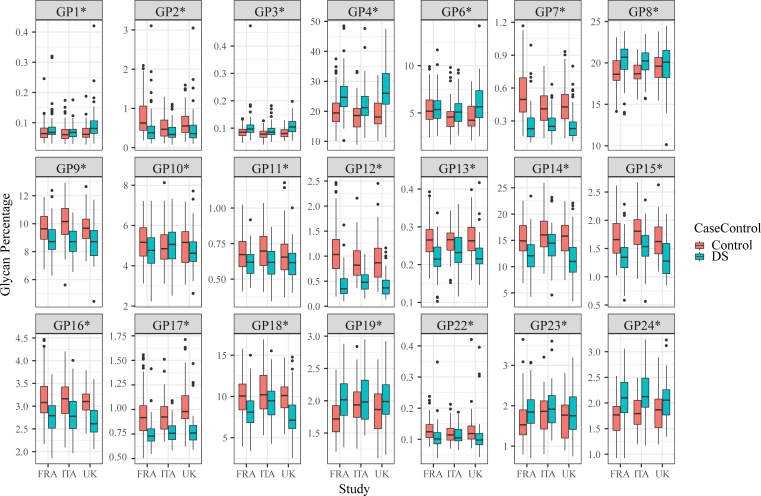


We analysed the glycosylation profiles of IgG in three independent European cohorts of people with DS from France, Italy and the UK (Supplementary Figures S1 and S2). The basic characteristics of the cohorts are given in Table 1, sex-disaggregated data are given in Supplementary Table S8 and a description of individual glycan peaks and derived traits are given in Methods, Supplementary Figure S1 and Supplementary Discussion.

**Table 1:** Characteristics of Down syndrome cohorts and healthy controls.

Characteristic	FRA, N = 207		ITA, N = 110		UK, N = 95	
	Control, N = 109	DS, N = 98	Control, N = 53	DS, N = 57	Control, N = 42	DS, N = 53
Age	46 (41–52)^a^	46 (43–52)^a^	38 (31–45)^a^	36 (29–45)^a^	47 (38–53)^a^	49 (44–53)^a^
Sex						
F	54 (50%)	51 (52%)	25 (47%)	24 (42%)	17 (40%)	22 (42%)
M	55 (50%)	47 (48%)	28 (53%)	33 (58%)	25 (60%)	31 (58%)
Autoimmunity	0 (NA)	34 (35%)	0 (NA)	22 (39%)	0 (NA)	25 (48%)
Thyroid Dis.	0 (NA)	23 (23%)	0 (NA)	24 (43%)	0 (NA)	24 (46%)
Dementia	0 (NA)	20 (20%)	0 (NA)	0 (NA)	0 (NA)	13 (25%)
FreqInfections	0 (NA)	15 (15%)	0 (NA)	16 (29%)	0 (NA)	7 (15%)

a Median (IQR).

**Fig. 1:**
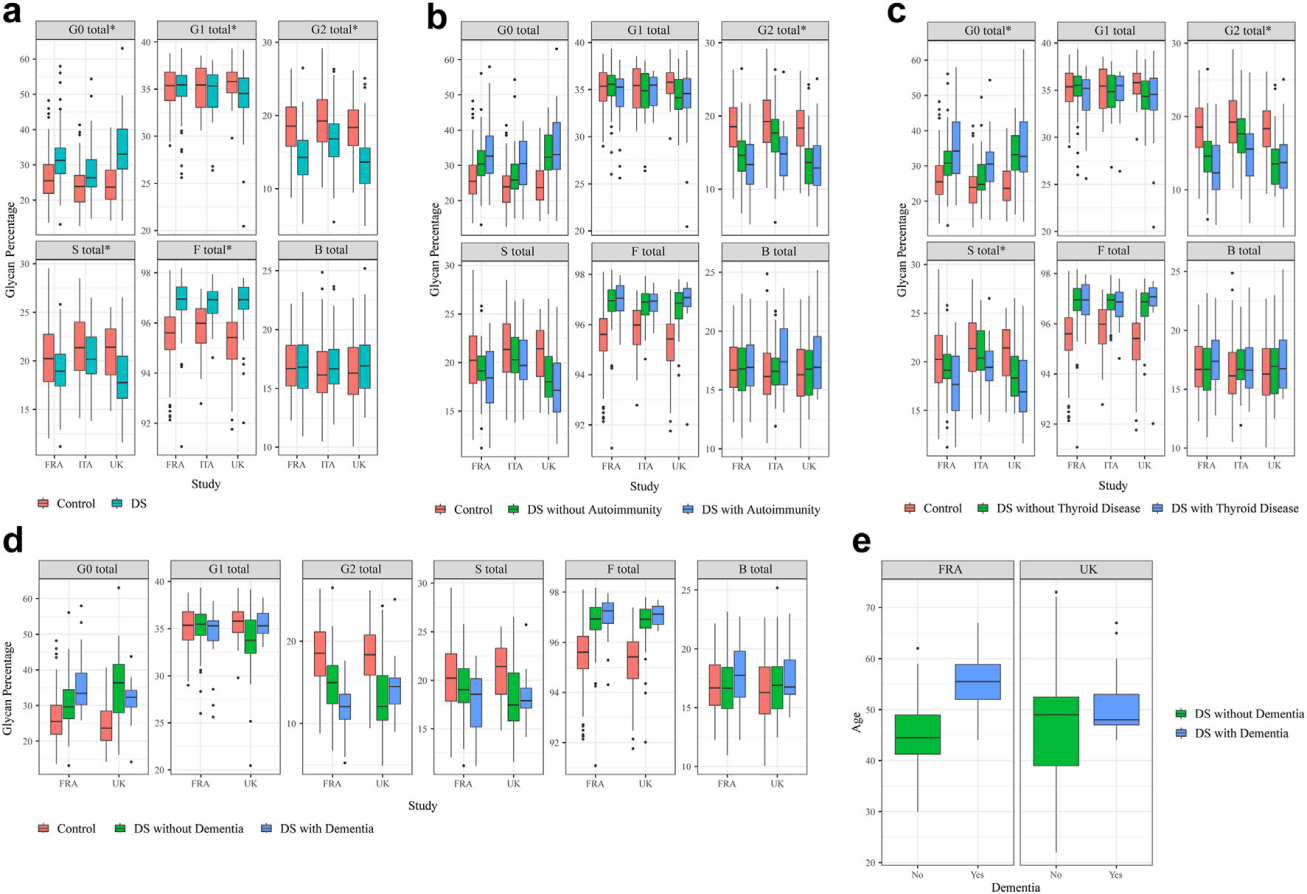
**Levels of six derived IgG glycan traits in persons with DS and in healthy controls shown separately for three cohorts of adults with DS from France (FRA), Italy (ITA) and United Kingdom (UK). (a)** Total data, not filtered for presence or absence of co-morbidities: G0 total–sum of IgG glycans without galactose, G1 total–sum of IgG glycans with one galactose, G2 total–sum of IgG glycans with two galactoses, S total–sum of IgG glycans with sialic acid(s), F total–sum of IgG glycans with core fucose, B total–sum of IgG glycans with bisecting N-acetylglucosamine (see Supplementary Figure S1 for definition of the traits). Statistical calculations and additional information are available in Supplementary Table S9 and Methods. **(b)** Comparison of levels of derived IgG glycan traits between controls and persons with DS with or without any type of autoimmune disease. **(c)** Comparison of levels of derived IgG glycan traits between controls and persons with DS with or without autoimmune thyroid disease (additional information is available in Supplementary Table S12). **(d)** Comparison of levels of derived IgG glycan traits between healthy control individuals, persons with DS without dementia and persons with DS with dementia. FRA—DS cohort from France, UK—DS cohort from the UK. No significant difference was observed between persons with DS with or without dementia (additional information is available in Supplementary Tables S11 and S12). **(e)** Age distribution of DS individuals with and without dementia within the French and UK cohorts of DS. For all graphs **(a–e)**, data are shown as box plots. Each box represents the 25th–75th percentiles (the interquartile range (IQR)). Lines inside the boxes represent the median. Lines outside the boxes indicate data within 1.5 × IQR from the 25th and 75th percentiles. Black dots indicate outliers. Asterisk ∗ indicates statistically significant differences (p < 0.05, meta-analysis) between DS individuals and healthy controls (statistical calculations and additional information is available in Supplementary Tables S9, S11 and S12 and Methods).

We examined the differences in IgG glycosylation between adults with DS and healthy subjects from the general population matched for age, sex and demography (except in the case of the DS cohort from France which remained unmatched for demography (see Methods)). Comparison of the derived IgG glycan traits revealed a higher level of IgG glycans without galactose (G0) (effect size 0.72) and IgG glycans with core fucose (F) (effect size 1.08) in persons with DS (Fig. 1a and Supplementary Tables S9 and S10). At the same time, the levels of IgG glycans with two galactoses (G2) (effect size −0.83) and IgG glycans with sialic acid(s) (S) (effect size −0.49) were lower in persons with DS compared to healthy controls (Fig. 1a). These observations were replicated independently in each of the three cohorts (Fig. 1a and Supplementary Tables S9 and S10). When all three cohorts were combined in the meta-analysis, the level of IgG glycans with one galactose (G1) was also found to be significantly lower in persons with DS than in controls (effect size −0.24) (Supplementary Table S9).

### Ageing-related IgG-glycan profile changes can be caused by trisomy 21 alone, in absence of co-morbidities

Next, we assessed the possibility that differences in IgG glycosylation observed between persons with DS and controls might not be associated with DS itself, but attributed to additional diseases occurring in persons with DS. To explore this, we divided persons with DS into those with and without a certain co-morbidity.

We first compared a group of people with DS without co-morbidity to a group of healthy, non-DS individuals. These results are presented in Fig. 1b–d and Supplementary Table S11, and a detailed description is given in Supplementary Discussion relating to these figures and table. In summary, for DS without diagnosed co-morbidities (Fig. 1b–d), we found nearly identical IgG glycan differences as for the whole (co-morbidity-unfiltered) DS cohorts (Fig. 1a), showing these alterations can be caused by T21 alone, as a genetic condition, with or without secondary effects of DS co-morbidities.

**Supplementary Figure S3:**
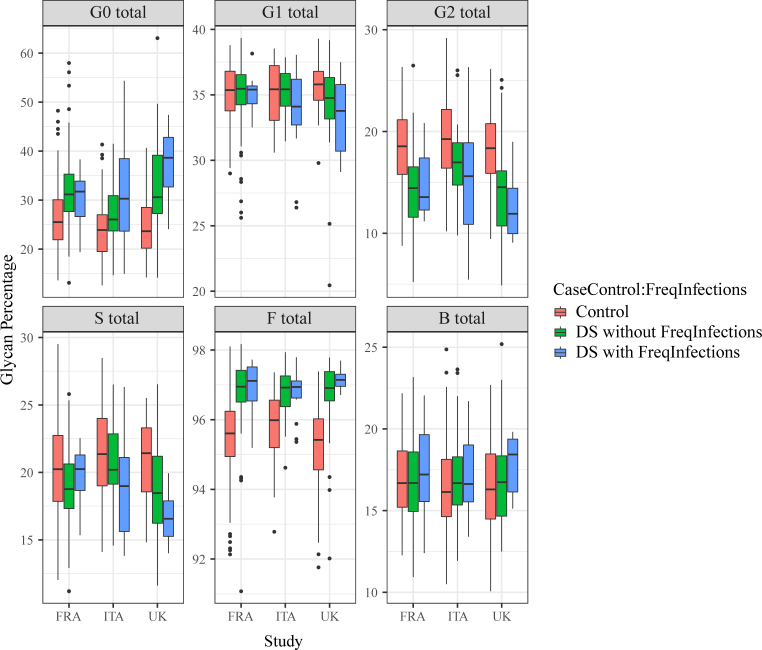


**Supplementary Figure S4:**
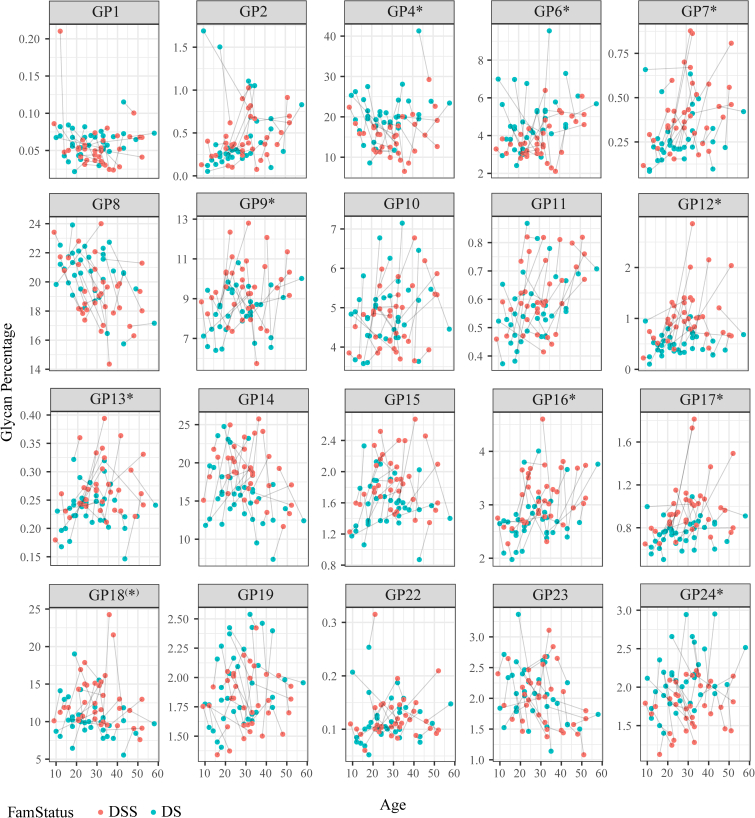


**Supplementary Figure S5:**
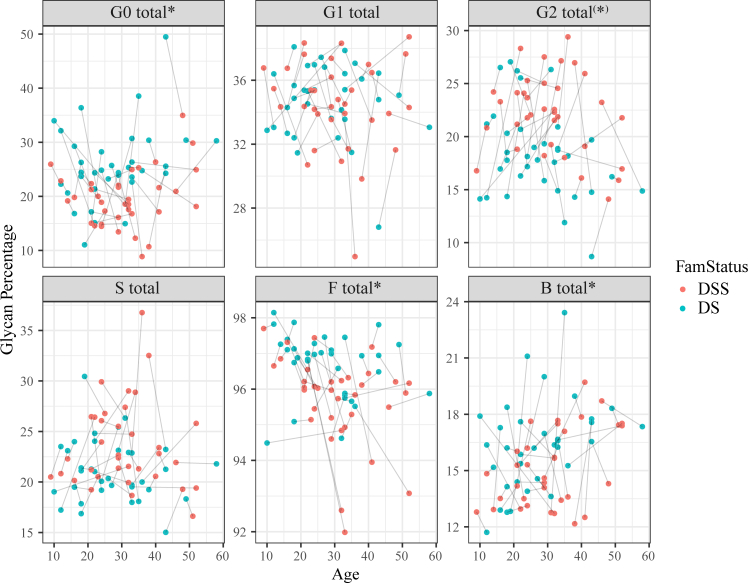


Secondly, we explored whether differences exist in IgG glycosylation between DS study participants with and without specific co-morbidities (Fig. 1b–d, Supplementary Figure S3, Supplementary Table S12, Supplementary Discussion). Slight additional effects (Fig. 1b and c) were found for autoimmune diseases and thyroid disease, namely a decrease in G2 for both (effect sizes −0.33 and −0.38, respectively), as well as an increase in G0 (effect size 0.4) and a decrease in S (effect size −0.47) specific to thyroid disease. Comparisons of DS IgG-glycan profiles to those of their euploid siblings (available for the Italian population) ruled out genotype differences as a driver of glycan profile changes (Supplementary Figures S4 and S5). Potential additional effects of clinical dementia (clinical data available for the UK and France populations) could not be excluded or confirmed, as they correlated strictly with age (Fig. 1d and e), just as the glycan profiles (for the detailed description of this section, please see Supplementary Discussion).

### IgG-glycan biological age of adults with DS is increased by 18.4–19.1 years compared to euploids matched by chronological age, and the rate of ageing does not increase throughout lifespan

**Fig. 2:**
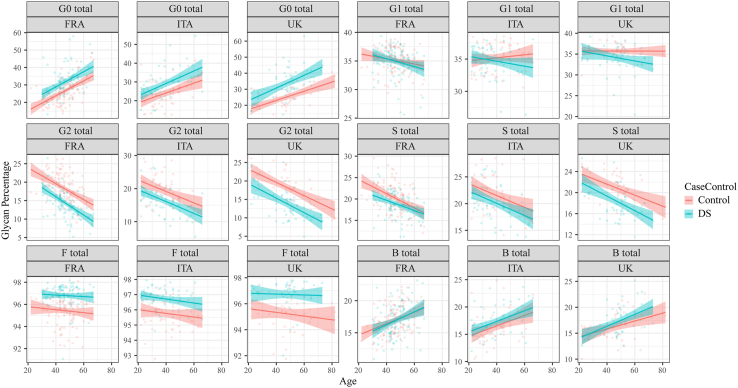
**Relationship between age and levels of six derived IgG glycan traits in persons with DS and in healthy controls shown separately for three cohorts of adults with DS from France (FRA), Italy (ITA) and the UK.** Red and blue lines represent fitted linear regression models for the control and DS data, respectively. The shaded region is a 95% confidence interval on the fitted values. Individual subject data points are presented on the background. G0 total–sum of IgG glycans without galactose, G1 total–sum of IgG glycans with one galactose, G2 total–sum of IgG glycans with two galactoses, S total–sum of IgG glycans with sialic acid(s), F total–sum of IgG glycans with core fucose, B total–sum of IgG glycans with bisecting GlcNAc. Trendline equations for the two derived traits that change the most with age: G0 FRA control: y = 0.43x + 6.72; G0 FRA DS: y = 0.44x + 11.42; G0 ITA control: y = 0.26x + 13.74; G0 ITA DS: y = 0.33x + 15.96; G0 UK control: y = 0.29x + 11.63; G0 UK DS: y = 0.40x + 14.92; G2 FRA control: y = −0.22x + 28.25; G2 FRA DS: y = −0.25x + 25.96; G2 ITA control: y = −0.17x + 26.04; G2 ITA DS: y = −0.18x + 23.23; G2 UK control: y = −0.18x + 26.79; G2 UK DS: y = −0.20x + 23.22.

Next, we examined whether persons with DS also exhibit changes in levels of IgG glycans with age and, if so, whether persons with DS show a similar pattern of changes in IgG glycans to that observed in the control group. We found that all derived IgG glycan traits whose levels were found to change significantly with age in the control group also changed significantly with age in persons with DS (Fig. 2 and Supplementary Table S13). Specifically, levels of G0 and B IgG glycans increased significantly with age whereas levels of G2 and S IgG glycans decreased significantly with age in persons with DS.

Next, we determined whether a difference exists in the extent of change in levels of derived IgG glycan traits with age between persons with DS and healthy controls. The shape and slope of plotted age-related glycan trend curves for persons with DS and controls were very similar (Fig. 2) and statistical analysis showed no difference in the rate of age-dependent changes in the level of derived IgG glycan traits (Supplementary Table S14). However, the position of curves showing IgG glycan levels as a function of age differed significantly between persons with DS and controls (Fig. 2). Specifically, we observed that the G0 glycan trait curve for persons with DS lays entirely above the curve corresponding to controls and that the G2 glycan trait curve for persons with DS lays entirely below the curve corresponding to controls. The amplified-genomic-instability model would predict a progressive increase in the rate of ageing for DS. The parallel slopes of the DS and euploid curves show that throughout the entire lifespan examined (age range 22–82) the extent of the IgG-glycan profile shift towards older biological age of DS does not change, i.e., the rate of ageing changes remains the same, contrary to the amplified instability model. The curve showing the level of G0 IgG glycans as a function of age (Fig. 2, three country panels on top left), allowed us to determine that, on average, the levels of G0 glycan trait in persons with DS corresponded to levels found in 18.4 years older individuals from the general population (France (FRA) 11.7–12.2 years (average 11.9 years); Italy (ITA) 15.1–20.0 years (average 17.5 years); UK 20.4–31.1 years (average 25.8 years)). Based on the curve showing the level of G2 IgG glycans as a function of age (Fig. 2, three country panels middle-left), we found that the level of G2 glycans in persons with DS corresponded to levels found in 19.1 years older individuals from the general population (FRA 15.0–17.6 years (average 16.3 years); ITA 17.1–17.9 years (average 17.5 years), UK 22.1–25.1 years (average 23.6 years)).

### IgG-glycan ageing acceleration is visible from early childhood

**Fig. 3:**
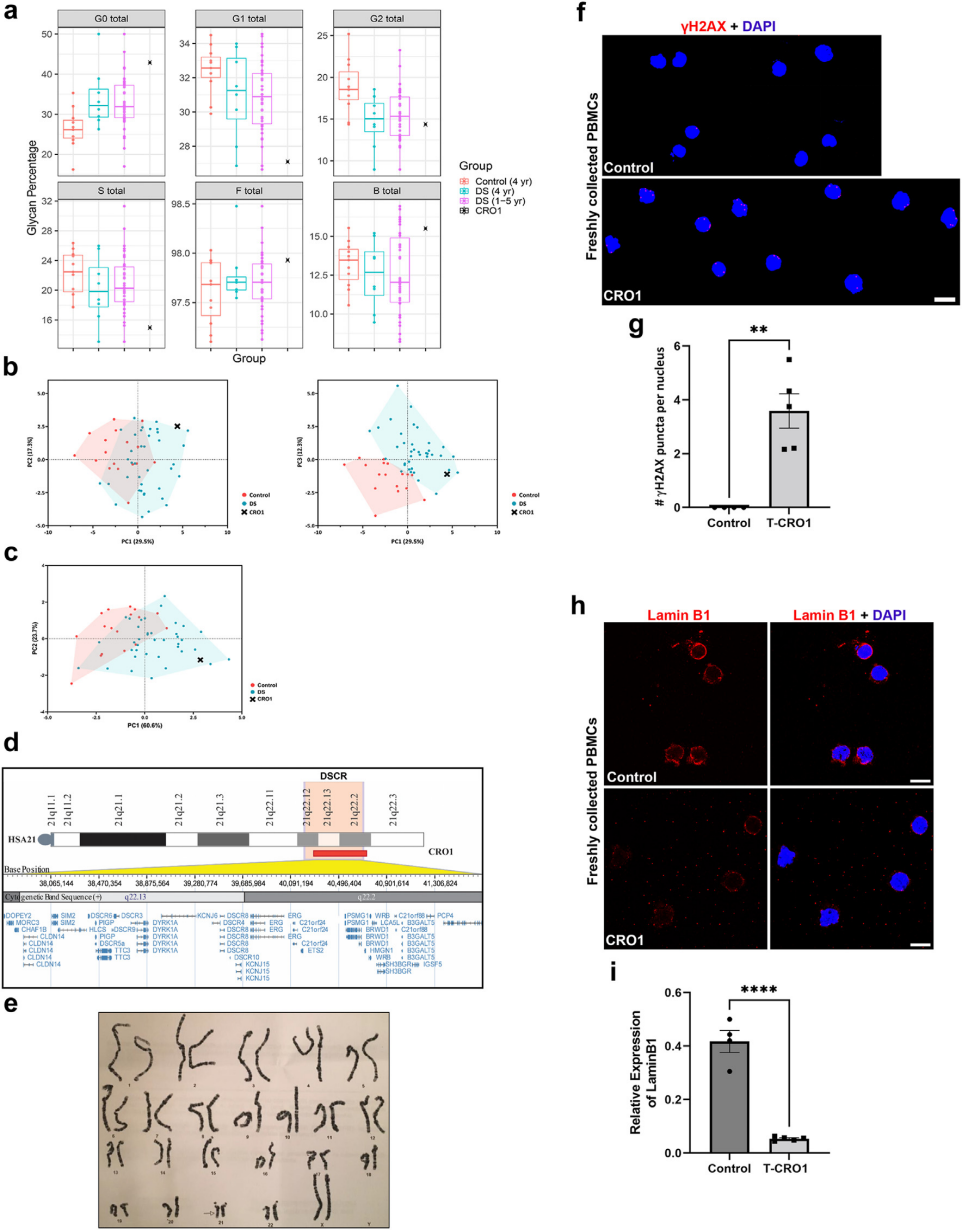
**IgG glycosylation in children with DS including a child with segmental T21 and cellular ageing marks in its blood cells. (a)** Levels of six derived IgG glycan traits in 4-year-old children with DS from the UK cohort (blue), age-matched 4-year-old healthy children (orange), in all DS children in the study aged 1–5 years (purple), and in CRO1 child with segmental T21 (black cross). G0 total–sum of IgG glycans without galactose, G1 total–sum of IgG glycans with one galactose, G2 total–sum of IgG glycans with two galactoses, S total–sum of IgG glycans with sialic acid(s), F total–sum of IgG glycans with core fucose, B total–sum of IgG glycans with bisecting GlcNAc. Data are shown as box plots with individual data points. Each box represents the 25th–75th percentiles (IQR). Lines inside the boxes represent the median. Lines outside the boxes indicate data within 1.5 × IQR from the 25th and 75th percentiles. Each dot represents one child. CRO1 child is marked as single black cross. **(b)** Principal component analysis (PCA) displaying differences in IgG glycosylation between all available DS children in the study and all available healthy control children: PC1 vs PC2 plot (left) and PC1 vs PC3 plot (right). PCA positioned CRO1 child with segmental T21 (marked as a black cross) within the DS sample cluster. Each dot represents one child. n (children with DS) = 38 + CRO1 child, n (euploid children) = 17. PCA was performed on directly measured IgG glycan peaks (GP1–GP24). **(c)** PCA on same cohorts as in part b, for G0, G1, G2, S and F derived IgG glycan traits values shows CRO1 clusters with the cohort of children with DS. **(d)** Graphical representation of the 4.083 Mbp segmental duplication in CRO1 which includes 31 chr21 genes spanning from *DOPEY2* to *PCP4*. The pale orange background indicates the region referred to as the “DSCR”, while the red bar indicates the region duplicated in CRO1, which is then magnified under the yellow region to show the gene content. **(e)** Full karyotype of the CRO1 child. Normal number of chromosomes, with a small duplication in the 21q22 region (arrow). **(f–i)** Fresh PBMCs at primary collection from the CRO1 child during a routine health check-up, fixed with 4% PFA and compared to those from an age- and sex-matched normal/euploid child by immunostaining with γH2AX and Lamin B1. CRO1 primary blood cells showed **(f and g)** increased levels of γH2AX puncta per nucleus, ∗∗ p = 0.0017, and **(h and i)** reduced levels of Lamin B1, ∗∗∗∗ p < 0.0001, compared to the control. 4–5 images per sample, with a total of approximately 50 cells per sample analysed. Data points on the graph represent the average value per image. Statistical significance was calculated using an unpaired 2-tailed student’s T-test. Error bars: SEM.

We next extended the study to a cohort of age-matched children with and without DS (see Methods). We observed that 4-year-old children with DS had a higher level of G0 IgG glycans compared to 4-year-old healthy children (Fig. 3a and Supplementary Table S15), shifting (with effect size 1.45) in the same direction as for the adult population (Fig. 1a). In addition, we observed a nominally significant (p = 0.052) decrease in the level of G2 IgG glycans in children with DS compared to healthy children (Fig. 3a and Supplementary Table S15). Bearing in mind the difficulty in seeing significant differences with such a small number of children, these results are compatible with children with DS showing the IgG-glycan trait shifts in the same direction of accelerated biological ageing as the adult DS population, indicating that mechanisms that cause accelerated IgG-glycan age in DS are active from infancy.

### Segmentally duplicated chr21 31-gene segment, without a freely segregating supernumerary chromosome, is sufficient to cause accelerated ageing marks in IgG-glycans and fresh blood cells of a child with DS

We next asked the question whether the presence of the freely segregating supernumerary chr21 is necessary to produce the IgG glycan shifts observed, which is one of the arguments behind the “amplified genomic instability” concept.

**Supplementary Figure S6:**
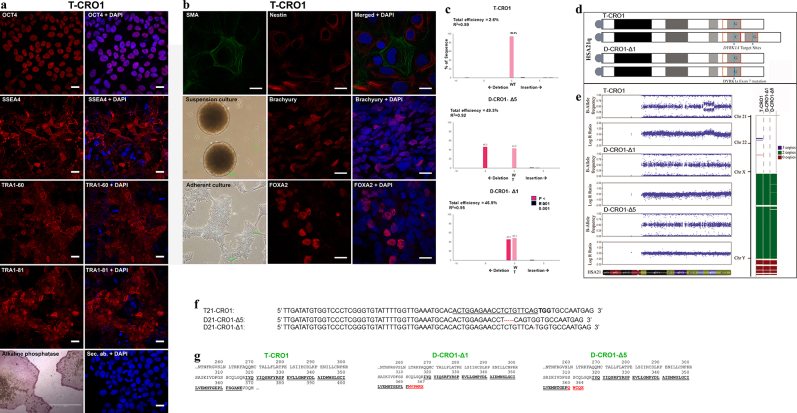


A 2-year-old child (labelled “x” in Fig. 3a–c) with signs of developmental delay and dysmorphic features in the spectrum of DS was analysed by karyotyping which showed a normal (euploid) karyotype of 46,XX (Fig. 3e). On high resolution banding, a small duplication in one copy of the chr21 was observed (Fig. 3e, arrow). A high-resolution SNP-array (Supplementary Figure S6) showed a segmental duplication of 4.083 Mbp on one chr21 copy, encompassing 31 genes (Fig. 3d) from (and including) *DOPEY2*, to (and including) *PCP4*, as the only genomic DNA anomaly. This corresponds to a region between chr21 co-ordinates 37,450,000–42,000,000 (GRCh37), which contains the entire duplication along with the proximal and distal disomic SNPs. This region approximately corresponds to the previous definition of the “Down syndrome Critical Region (DSCR)”,^42^^,^^43^ a region on chr21 that was originally thought to be responsible for many features of DS, so we named this sample “Critical Region Only-1 (CRO1)”. The values for IgG glycan traits: G0 (higher); G1, G2 and S (lower) for this child (Fig. 3a, marked as a single black cross) were all outside the range of the euploid 4-year-old children (n = 11), and clearly segregating with 1–5 year-old children with DS on derived glycan trait graphs (Fig. 3a), as well as on two different ways of calculating the Principal Component Analysis (PCA) using directly measured IgG glycan peak values (GP1–GP24) (Fig. 3b), or the PCA using derived IgG glycan trait parameters (Fig. 3c). The values for 4 year-olds with DS are also separately shown as they were the only ones exactly age-matching the controls. In this comparison, the CRO1 child gave IgG glycan values clearly more extreme than the mean of the DS group that was already statistically significantly different from the euploid controls’ mean (Fig. 3a). The data clearly indicate that the presence of the freely segregating supernumerary chr21 might not be necessary for the accelerated IgG-glycan ageing profiles, but that trisomy of one or more of chr21 genes in the CRO1 duplicated segment could be sufficient to produce the IgG glycome profile changes observed for DS very early in life.

In peripheral blood samples of DS, we found significantly increased biological age of DS populations using IgG glycan profiling (Figs. 1 and 2). This is concordant with previously observed increased DNA damage in DS primary peripheral blood mononuclear cells (PBMCs).^19^, ^20^, ^21^ We wanted to see if the same phenotype reproduces in PBMCs from the CRO1 child, given its IgG-glycan profile was clearly segregating with DS, and not euploid/control children. A limited number of PBMCs from the CRO1 child was collected fresh as consented surplus diagnostic material, and compared to fresh PBMCs of another typically developing child of the same age and sex. This allowed us to examine the cells without any stress impacted by freezing-thawing or cell culturing. These cells were spread, fixed and stained for γH2AX, a nuclear marker of DNA double-strand breaks (DSB) repair foci. Quantification of γH2AX showed a very significant increase of γH2AX from near zero γH2AX puncta per nucleus in the control child to an average of 3.59 (p = 0.0017, t-test) in CRO1 (Fig. 3f and g), providing a hitherto untested genetic link between IgG glycan ageing marks and cellular DNA damage by finding that segmental overdose of chr21 31 genes is sufficient to cause both phenotypes.

DNA damage is one of the stimuli that can cause cellular senescence. A reduction in levels of the nucleo-skeletal protein Lamin B1 is a highly reliable marker of cellular senescence of multiple cell types,^44^ accompanying physiological cellular ageing^45^ and particularly pronounced in some premature ageing syndromes, such as the laminopathy Hutchinson Guilford Progeria Syndrome (HGPS).^46^, ^47^, ^48^ We therefore stained the same fresh PBMCs with a Lamin B1-specific antibody. The CRO1 primary cells showed a very significant decrease in relative Lamin B1 level compared to an age/sex-matched control (from 0.42 to 0.05 (p < 0.0001, t-test), Fig. 3h and i). This suggests that an overdose of one or more genes resident in the CRO1 duplication can cause systemic progeroid signs, accompanied by Lamin B1-type laminopathy, increased DNA damage and cellular senescence.

### Spontaneous DSBs of undifferentiated hiPSCs are further increased by full and segmental T21, driven by the dose and kinase activity of DYRK1A

**Fig. 4:**
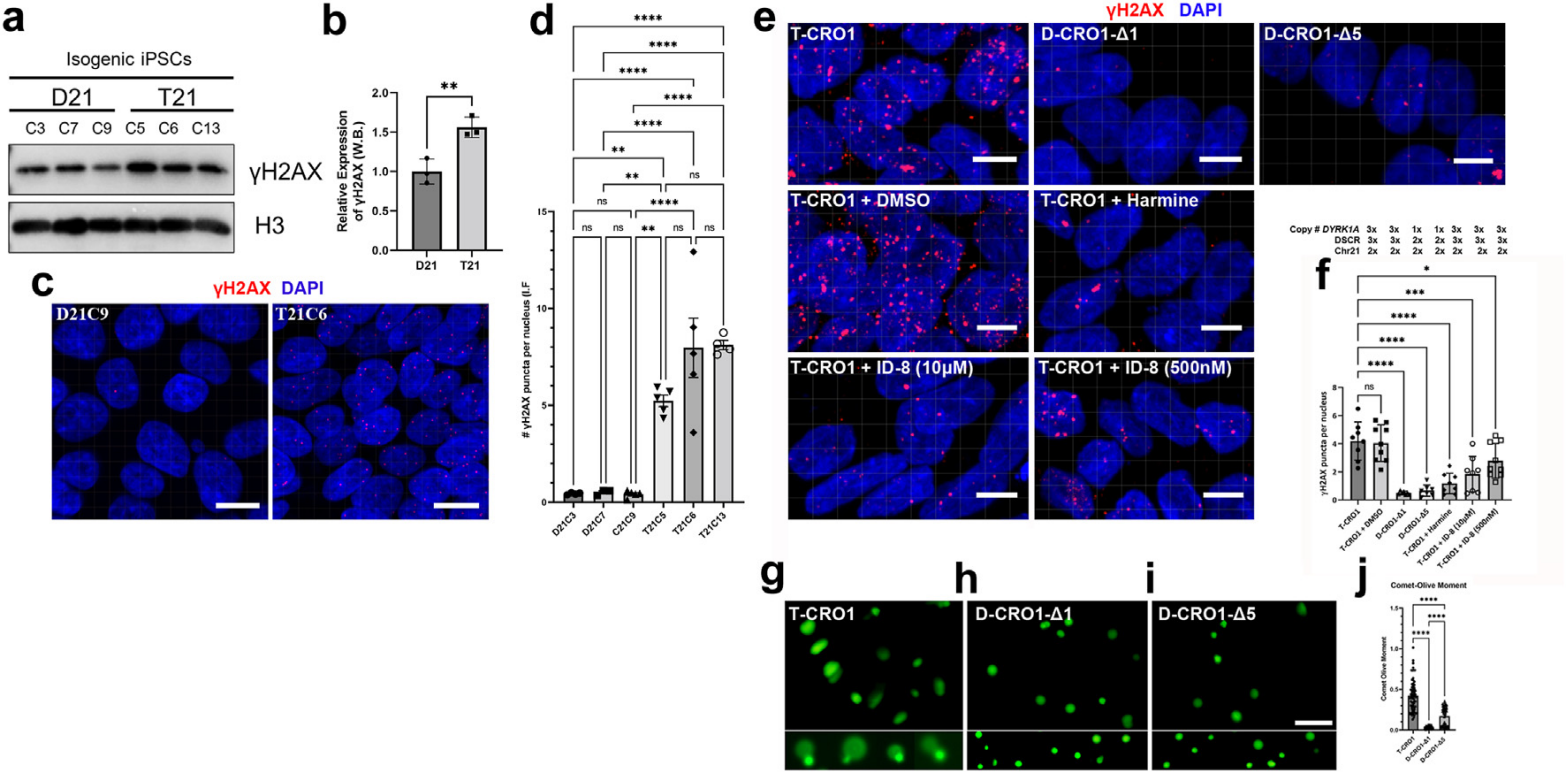
**Increased levels of γH2AX in isogenic models of full and partial trisomy 21. (a)** Histones were purified from 3 disomic (D21) and 3 trisomic (T21) isogenic iPSC clones. Increased levels of γH2AX were observed in each of the three independent clones of T21 iPSCs as compared to the three independent isogenic D21 iPSCs. Histone H3 was used as histone purification and loading control in Western blotting. **(b)** Relative expression of γH2AX to H3 was quantified and represented as individual dots using D21 levels as 1. Error bars show SEM (unpaired Student’s 2-tailed t-test, p = 0.0092). **(c)** Representative immunofluorescence images used for analysis show an increased number of γH2AX puncta per nucleus in T21 compared to isogenic D21 iPSC clones. Scale bar 5 μm. **(d)** Numbers of γH2AX puncta per nucleus in T21 and D21 iPSC clones. Sufficient images for each cell line were taken to ensure a minimum of 2000 nuclei counted. Each dot on the histogram represents the data of an individual image. Error bars: SEM. Statistical analysis was carried out by one-way ANOVA followed by Tukey’s correction for multiple comparisons. **(e)** T-CRO1 iPSCs were treated with DMSO or DYRK1A inhibitors (harmine or ID-8) for 12 h before fixation alongside untreated D-CRO1-Δ1 and D-CRO1-Δ5. Representative confocal images used for analysis of γH2AX stainings are shown. Scale bars: 5 μm. **(f)** Quantification of γH2AX foci per nucleus was performed automatically using IMARIS. Sufficient images were taken from two independent experiments consisting of three wells each to ensure that a minimum of 2000 nuclei were counted for each cell line. Each dot on the histogram represents the data of an individual image. Statistical significance was calculated by one-way ANOVA followed by Dunnett’s correction for multiple comparisons. Error bars: SEM. **(g–i)** Representative images from T-CRO1, D-CRO1-Δ1 and D-CRO1-Δ5 iPSCs used for analysis with the OpenComet plug-in for ImageJ. The top panels show one field of view, zoomed out. Scale bar 50 μm. The lower panel shows zoomed and cropped single cells which the ImageJ plug-in recognised as single, non-overlapping cells. DNA tail is visible in parental, T-CRO1 clone. **(j)** A minimum of 70 well-separated cells were analysed per cell line in the Comet assay to calculate the Comet-Olive Moment. One-way ANOVA followed by Tukey’s correction for multiple comparisons demonstrated a significant reduction in DNA damage in the CRISPR clones. Error bars: SEM. For all parts: ∗p < 0.05, ∗∗p < 0.01, ∗∗∗p < 0.001, ∗∗∗∗p < 0.0001, ns = not significant.

Basic levels of DSBs, without any genotoxic treatments, were found to be intrinsically elevated in undifferentiated hiPSCs grown in culture, significantly above the levels seen in primary cells from which the iPSCs were generated, and above the levels in downstream cells differentiated from iPSCs.^49^ These DSBs were found to be the result of the rapid cell replication state of hiPSCs with low level of co-localisation with 53BP1 (a non-homologous end joining-DNA damage response (NHEJ-DDR) mediator).^49^ We previously established an isogenic full chromosome T21 and D21 hiPSC model^14^ successfully used to model the APP processing in DS.^39^ We first wanted to establish to what extent the γH2AX foci were visible in undifferentiated iPSCs of our isogenic T21:D21 model, and whether T21 modulated the basic level of DSB foci. Using both immunofluorescence, and immunoblotting of histones, we measured an increased presence of γH2AX in three independent T21 iPSC lines (C5, C6 and C13) compared to three independent isogenic D21 iPSC lines (C3, C7, C9) (Fig. 4a–d). Immunoblotting of purified histones showed a 1.56-fold increase in γH2AX in T21 compared to D21 iPSCs (Fig. 4a and b), while immunofluorescence showed an increase in γH2AX puncta per nucleus from 0.42–0.52 in D21 to 5.22–8.1 in T21 iPSCs (Fig. 4c and d). This suggests that T21 causes a cell-autonomous increase in DNA damage that can be modelled using undifferentiated iPSCs.

Since CRO1 primary PBMCs showed significantly increased γH2AX foci, and its plasma IgG-glycan profile segregated with full trisomy DS, we set out to generate an iPSC model from the CRO1 child. Such a model would also be particularly useful as a genetic dissection tool for cellular phenotypes since the genetic anomaly in CRO1 is narrowed to a trisomic overdose of only 31 chr21 genes (once called the DSCR). A sample of CRO1 primary blood cells was reprogrammed into iPSCs, via the non-integrational Sendai virus method, optimised for small primary blood samples^50^ (see Methods and Supplementary Figure S6a and S6b).

From within the CRO1 duplicated region, *DYRK1A* was selected as a candidate gene based on its multiple reported dose-sensitive effects on brain abnormalities in DS, including neurodevelopment, retina development and Alzheimer’s type neurodegeneration,^51^, ^52^, ^53^, ^54^ as well as its interaction with proteins involved in DNA-repair^55^, ^56^, ^57^ and cellular quiescence.^58^ We aimed to correct the copy number of *DYRK1A* to disomy, using CRISPR-Cas9 gene targeting, in the same way as we recently accomplished in T21 iPSCs, for the chr21 gene *BACE2*.^39^ The CRISPR-Cas9 editing produced knockout mutations in two separate clones (hereinafter referred to as D-CRO1-Δ1 and D-CRO1-Δ5 with a deletion of 1 bp and a deletion of 5 bp, respectively), both changing the reading frame and introducing a translation stop codon (Supplementary Figure S6c–S6g). The “Tracking InDels by dEcomposition” (TIDE) quantification, surprisingly, gave 50:50 ratios of *DYRK1A* mutated and wild-type sequence in both mutants (Supplementary Figure S6c). Using SNP-arrays, we verified that the CRISPR-guide-RNA hit the *DYRK1A* locus in both copies of the segmental duplication, and the repair mechanism simply excised everything in between the two gRNAs (Supplementary Figure S6d and S6e), thereby correcting to disomy the entire CRO1 duplicated segment (turning T-CRO1 into D-CRO1), while additionally introducing Δ1 and Δ5 mutations, respectively in each clone (Supplementary Figure S6c–S6g). This means that D-CRO1-Δ1 and D-CRO1-Δ5 clones have a functional disomy of all 31 chr21 genes in the CRO1 region, with the exception of *DYRK1A*, which was turned into functional monosomy in each clone. Though not producing an exact genetic model of *DYRK1A* disomy, we proceeded to analyse these isogenic models, as we speculated that any gene dose-driven effects would be even more pronounced in isogenic lines reduced for *DYRK1A* copy number from 3 to 1.

Since CRO1 PBMCs showed a robust increase in γH2AX compared to euploid PBMCs of the same age and sex (Fig. 3f and g), we hypothesised that correction of T-CRO1 to D-CRO1 could verify whether the presence of the spontaneously generated γH2AX foci in hiPSCs can further be affected/modulated by the segmental duplication. First, we observed that T-CRO1 hiPSCs display increased numbers of γH2AX foci per nucleus (concordant with the effect seen in full chromosome T21 hiPSCs) (Fig. 4c–f). Second, the D-CRO1-Δ1 and D-CRO1-Δ5 clones showed significantly less γH2AX foci than T-CRO1 hiPSCs (Fig. 4e and f), reminiscent of the difference observed when CRO1 primary cells were compared to a disomic control (Fig. 3f and g). To further evaluate the specific role of DYRK1A overdose in T-CRO1 on these measurements, we treated the T-CRO1 iPSCs using two DYRK1A kinase inhibitors: harmine and ID8. Both treatments significantly reduced the levels of γH2AX to 0.28 (harmine 300 nM), 0.44 (ID8 10 μM) and 0.66 (ID8 500 nM) relative to the levels detected in untreated cells set to 1.0 (Fig. 4e and f). This strongly suggests that DYRK1A dose and kinase activity are responsible for additionally increasing the number of γH2AX foci in T-CRO1, over and above the basic iPSC endogenous levels.

**Supplementary Figure S7:**
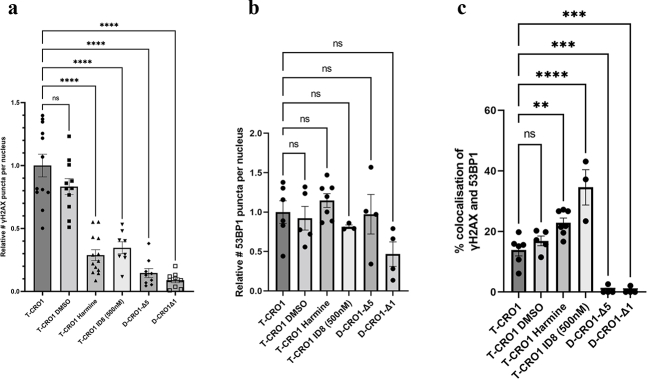


As DYRK1A was recently found interacting with proteins involved in DNA repair,^55^, ^56^, ^57^ we repeated once more the γH2AX staining on the same iPSC panel, this time co-staining with 53BP1 (NHEJ-DDR mediator) (Supplementary Figure S7). The relative profile of γH2AX levels seen in Fig. 4f was fully reproduced again in the cell lines and upon DYRK1A inhibitors treatment (Supplementary Figure S7a). We observed that harmine and ID8 treatments both improved the co-localisation of γH2AX and p53BP1 foci, from an average of 13.8%–22.9% and 34.6%, respectively, indicating that chemical inhibition of DYRK1A kinase might increase the efficiency of DDR (Supplementary Figure S7c). In D-CRO1-Δ1 and D-CRO1-Δ5 clones, we observed that 53BP1 co-localisation with γH2AX was almost zero (Supplementary Figure S7c), probably because γH2AX foci number dropped to background levels. Put together, these data demonstrate that trisomic increase in DYRK1A kinase activity is necessary to significantly de-range the DDR process, in line with the recently observed interaction of DYRK1A with proteins of DDR. In order to evaluate if this derangement decreases the repair efficiency, we sought to verify whether the excess γH2AX foci in our model iPSC lines really represent unrepaired DSBs. To this end, we performed the COMET assay, which verified the significant excess of unrepaired DSBs for T-CRO1, compared to D-CRO1-Δ1 and D-CRO1-Δ5 iPSCs (Fig. 4g–j). Put together, these data provide at least one mechanism by which the trisomic dose of DYRK1A in DS might cause accelerated cellular ageing and senescence.

**Fig. 5:**
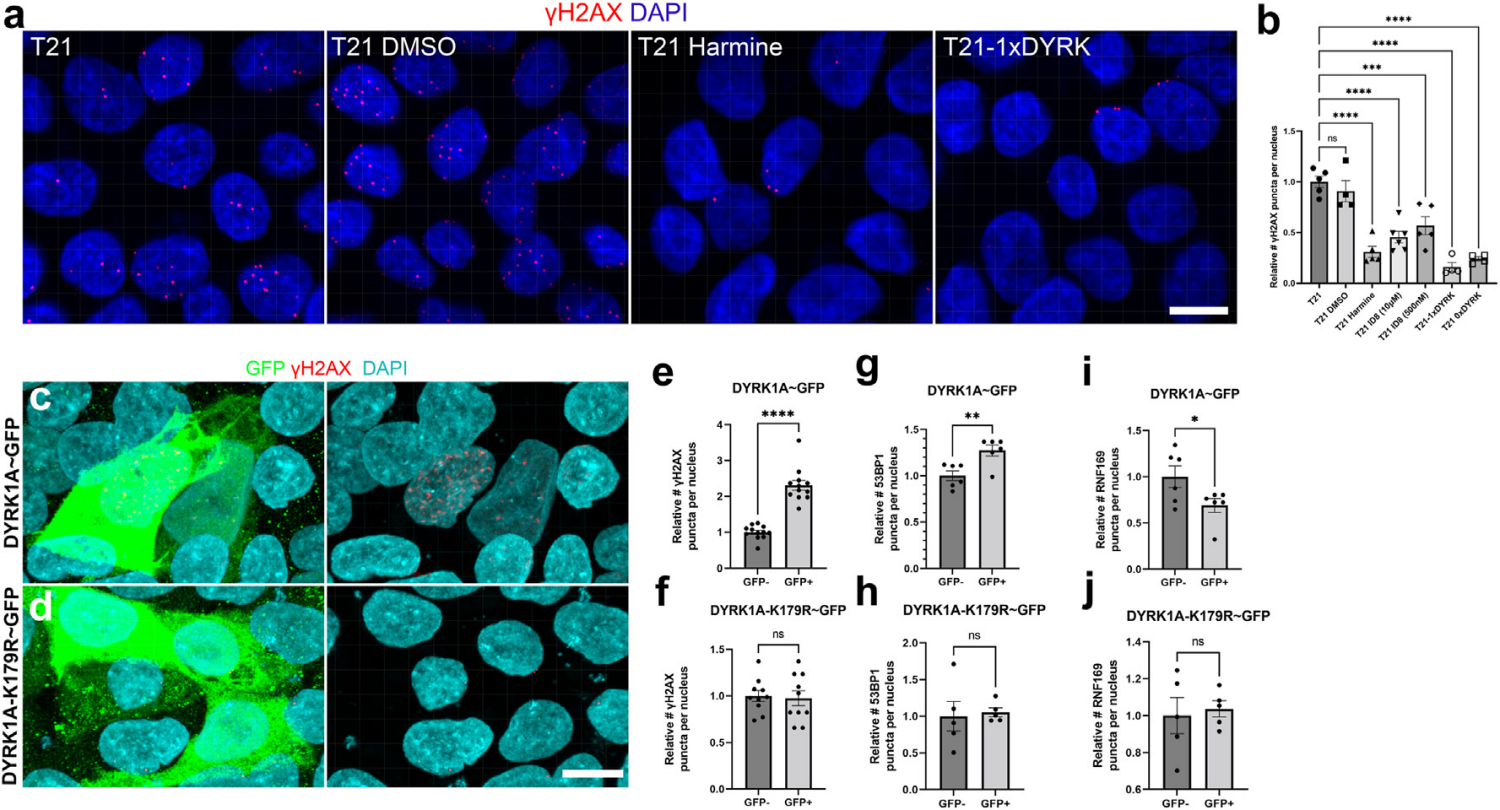
**Number of γH2AX foci per nucleus in T21 iPSCs is driven by the dose of *DYRK1A*. (a)** Representative images of T21 iPSCs used for analysis, treated with DMSO or a DYRK1A inhibitor (300 nM harmine) for 12 h alongside untreated T21 and T21-1xDYRK. iPSCs were fixed and stained for γH2AX then imaged by confocal microscopy. Scale bar: 10 μm. **(b)** Quantification of γH2AX foci was performed automatically using IMARIS. Sufficient images for each cell line were taken to ensure a minimum of 2000 nuclei were counted. Data are shown relative to T21 in which γH2AX puncta per nucleus was set to 1. Each dot on the histogram represents the data of an individual image. Error bars: SEM. Statistical significance was calculated by one-way ANOVA followed by Dunnett’s correction for multiple comparisons. **(c and d)** T21-0xDYRK iPSCs were transiently transfected with a plasmid to express wild-type DYRK1A or a kinase-dead mutant (K179R) DYRK1A (and GFP under the control of an IRES). Cells were fixed 72 h post-transfection, stained with γH2AX then imaged by confocal microscopy. Representative images used for analysis are shown. Scale bars: 10 μm. **(e–j)** Quantification of γH2AX and 53BP1 foci was performed automatically using IMARIS. Sufficient images for each cell population were taken to ensure a minimum of 200 nuclei were counted. Each dot on the histogram represents the data of an individual image. The number of γH2AX puncta in cells expressing the exogenous DYRK1A (GFP^+^ cells) was compared to that in DYRK1A null (untransfected GFP^−^ cells) on the same coverslip, for both the **(e)** wild-type and **(f)** mutant DYRK1A. The number of 53BP1 **(g and h)** and RNF169 **(i and j)** puncta in cells expressing the exogenous DYRK1A (GFP^+^ cells) was compared to that in DYRK1A null (untransfected GFP^−^ cells) on the same coverslip, for both the **(g and i)** wild-type and **(h and j)** mutant DYRK1A. In each case values are shown relative to the value of GFP^−^ cells set to 1. Error bars: SEM. Statistical significance was calculated using an unpaired 2-tailed Student’s T-test. For all parts: ∗p < 0.05, ∗∗p < 0.01, ∗∗∗p < 0.001, ∗∗∗∗p < 0.0001, ns = not significant.

**Supplementary Figure S8:**
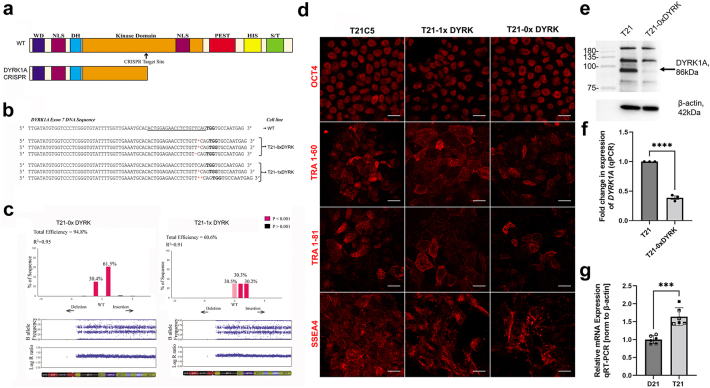


In order to confirm the causative link between the trisomic dose and kinase activity of DYRK1A and increased DSB in undifferentiated iPSCs, we targeted DYRK1A in the full chromosome T21 hiPSC model. After establishing that all T21 isogenic clones show a nearly identical (1.6×) increase in *DYRK1A* mRNA level by qRT-PCR (Supplementary Figure S8g), CRISPR-Cas9 targeting in a T21 iPSC clone C5 from this model produced T21-1xDYRK and T21-0xDYRK iPSC lines (Supplementary Figure S8). Reduction of DYRK1A dose in both of these lines, as well as treatment with harmine or ID8 inhibitors, reduced the number of γH2AX foci per nucleus relative to untreated, or vehicle-treated T21-C5 iPSC line by two to five-fold (Fig. 5a and b). Transient overexpression of wild-type DYRK1A, but not of its kinase-dead mutant (DYRK1A-K179R), each with an independently translated GFP tag, increased the number of γH2AX and 53BP1 foci in T21-0xDYRK iPSCs (Fig. 5c–f). Compared to un-transfected cells, cells overexpressing wild-type DYRK1A showed an increase in γH2AX puncta per nucleus by 2.3-fold (p < 0.0001, t-test), while those overexpressing DYRK1A-K179R showed no difference (p = 0.8091, t-test). Similarly, wild-type DYRK1A overexpression increased 53BP1 (NHEJ-DDR protein) puncta per nucleus by 1.27-fold (p = 0.0065, t-test), with no difference detected in cells expressing DYRK1A-K179R (p = 0.8033, t-test) (Fig. 5g and h), while significantly decreasing the RNF169 (Homologous Recombination (HR) DDR protein) puncta (Fig. 5i and j). Put together, these experiments verify that effects of trisomic DYRK1A dose and kinase activity on DDR derangement are reproducible in isogenic iPSC systems from two unrelated individuals, and that in the context of the full chr21 trisomy, DYRK1A dose and kinase activity are both necessary and sufficient to explain the increase in DSB in DS iPSCs.

### A decrease in Lamin B1 is caused by trisomy of *DYRK1A* in undifferentiated iPSCs from full and segmental T21 models and reproduces in multiple DS foetal and infant tissues

**Fig. 6:**
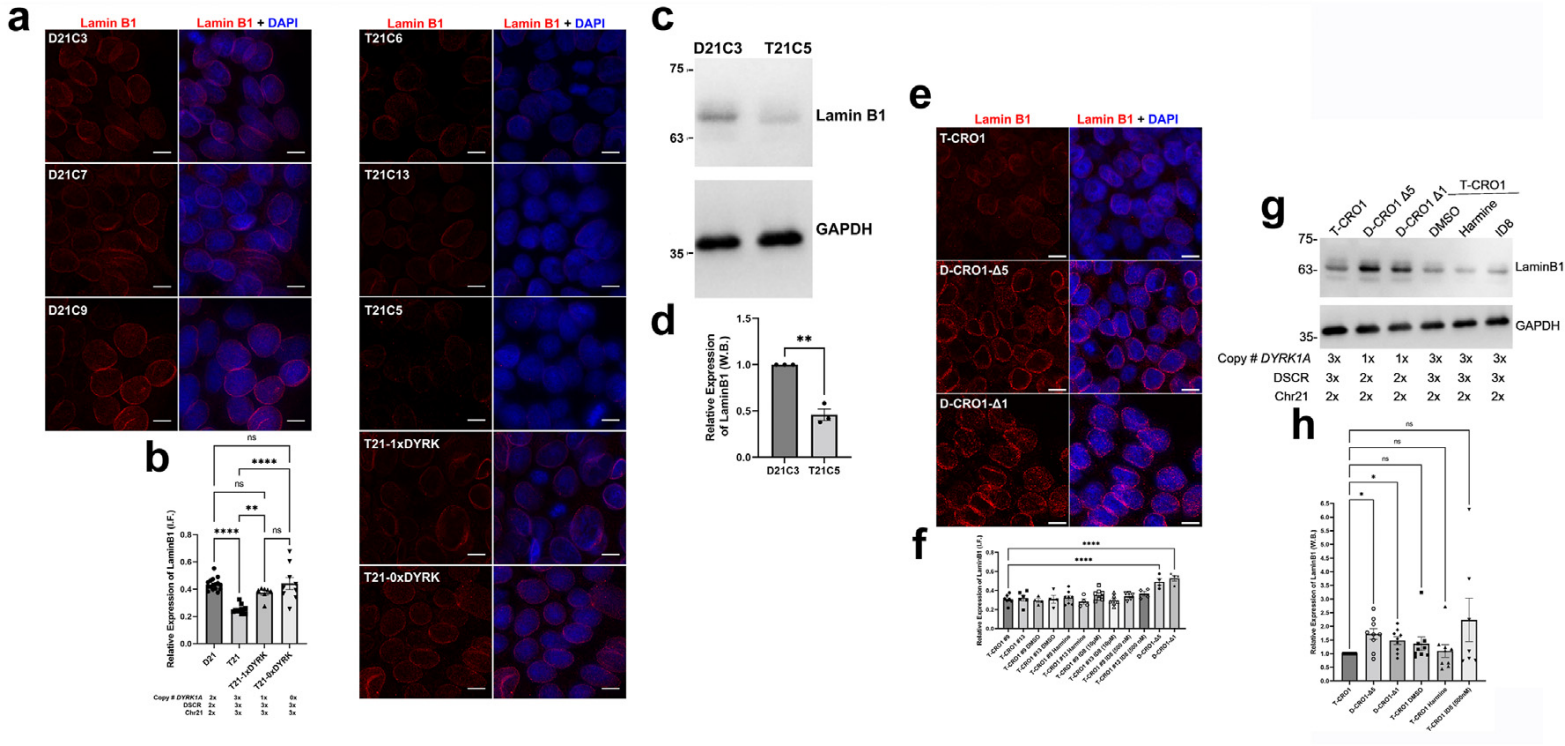
**Expression of Lamin B1 in T21 and T-CRO1 iPSCs is inversely correlated with the dose of *DYRK1A*. (a)** Representative images from independent isogenic T21 and D21 iPSC clones alongside the isogenic CRISPR/Cas9-edited T21-1xDYRK and T21-0xDYRK were stained for Lamin B1 then imaged by confocal microscopy. Scale bars: 10 μm. **(b)** The relative expression of Lamin B1 in a minimum of 4000 DAPI positive nuclei from 4 or more images per cell line were analysed. Each dot on the histogram represents the data of an individual image. Quantification of Lamin B1 expression was performed using IMARIS and normalised to DAPI. Error bars: SEM. Statistical significance was calculated by one-way ANOVA followed by Dunnett’s correction for multiple comparisons: D21 vs T21, p < 0.0001; T21 vs T21 1xDYRK, p = 0.0044; T21 vs T21-0xDYRK, p < 0.0001. **(c)** Lamin B1 expression in iPSC lysates was analysed by Western blot, which showed reduced Lamin B1 expression in T21 iPSCs compared to isogenic D21 iPSCs relative to the GAPDH loading control. **(d)** The Lamin B1 signal in three independent Western blots was quantified and plotted relative to that of GAPDH with the values in D21C3 set as 1. Error bars: SEM. Statistical significance was calculated with an unpaired two-tailed Student’s t-test (p = 0.001). **(e and f)** T-CRO1 iPSCs (2 independent clones #9 and #13) were treated or not with DMSO or a DYRK1A inhibitor for 12 h before fixing or harvesting alongside untreated D-CRO1-Δ1 and D-CRO1-Δ5. iPSCs were fixed and stained for Lamin B1 then imaged by confocal microscopy. Representative images of the untreated iPSC clones are shown in **(e)**. Scale bars: 10 μm. In **(f)**, quantification of Lamin B1 expression was performed using IMARIS and normalised to DAPI. The relative expression of Lamin B1 in n > 4000 DAPI positive nuclei were counted for each cell line. Each dot on the histogram represents an individual image. Error bars: SEM. Statistical significance was calculated by one-way ANOVA followed by Dunnett’s correction for multiple comparisons, comparing each cell line/condition to untreated T-CRO1 #9. Only significant comparisons to T-CRO1 are shown on the graph (∗∗∗∗p < 0.0001). A similar analysis comparing everything to T-CRO1 #13 gave a similar result with only D-CRO1-Δ1 and D-CRO1-Δ5 showing significance (not shown). **(g and h)** iPSC lysates were analysed by Western blot for Lamin B1 expression. In **(g)**, a representative blot is shown. In **(h)**, the signal of Lamin B1 was quantified in independent Western blots, normalised to GAPDH, and set as 1 in T-CRO1 iPSCs Error bars: SEM. Statistical significance was calculated by fitting a mixed model followed Dunnett’s correction for multiple comparisons (D-CRO1-Δ1 vs T-CRO1, p = 0.0345; D-CRO1-Δ5 vs T-CRO1, p = 0.0224). For all parts: ∗ p <0.05, ∗∗ p <0.01, ∗∗∗∗ p <0.0001, ns = not significant

The cellular senescence marker Lamin B1 was quantified by immunofluorescence and three independent T21 lines showed significantly reduced Lamin B1 signal when compared to three independent D21 lines (p < 0.0001, ANOVA), or when compared to both T21-1xDYRK (p = 0.0044, ANOVA) and T21-0xDYRK (p < 0.0001, ANOVA) hiPSC lines (Fig. 6a and b). A short treatment with harmine or ID8, or transient overexpression of DYRK1A did not significantly alter Lamin B1 signals (not shown), possibly due to the long half-life of Lamin B1 protein.^59^ Comparing D21-C3 to T21-C5 iPSCs by immunoblotting further validated that Lamin B1 expression was reduced in T21-C5 (Fig. 6c and d).

Notably, a decrease in Lamin B1 levels was observed in two independent iPSC clones of T-CRO1 (#9 and #13) when compared to isogenic D-CRO1-Δ1 and D-CRO1-Δ5 clones (Fig. 6e and f). This result is concordant with the phenotype in CRO1 PBMCs compared to control samples (Fig. 3h and i). As a control for clonal variation in iPSC lines, T-CRO1 #9 and T-CRO1 #13 showed no significant difference in Lamin B1 expression. Immunoblotting also showed a significant increase in Lamin B1 expression in both D-CRO1-Δ1 and D-CRO1-Δ5 compared to T-CRO1 (p = 0.0345 and p = 0.0224, respectively, mixed-model) (Fig. 6g and h). In contrast to gene dose reduction by CRISPR/Cas9, chemical inhibition of DYRK1A had no significant effect in either immunostaining (Fig. 6e and f) or immunoblotting (Fig. 6g and h). This could mean that genes other than *DYRK1A* in the CRO1 region cause the Lamin B1 decrease, or that it is a non-kinase effect of DYRK1A dose. However, this could also be the result of the very long half-life of Lamin B1 protein,^59^ whereby a 12-h drug treatment is too short to show an effect (we corroborate this explanation later in the manuscript, after a prolonged drug treatment of cerebral organoids, see below).

**Fig. 7:**
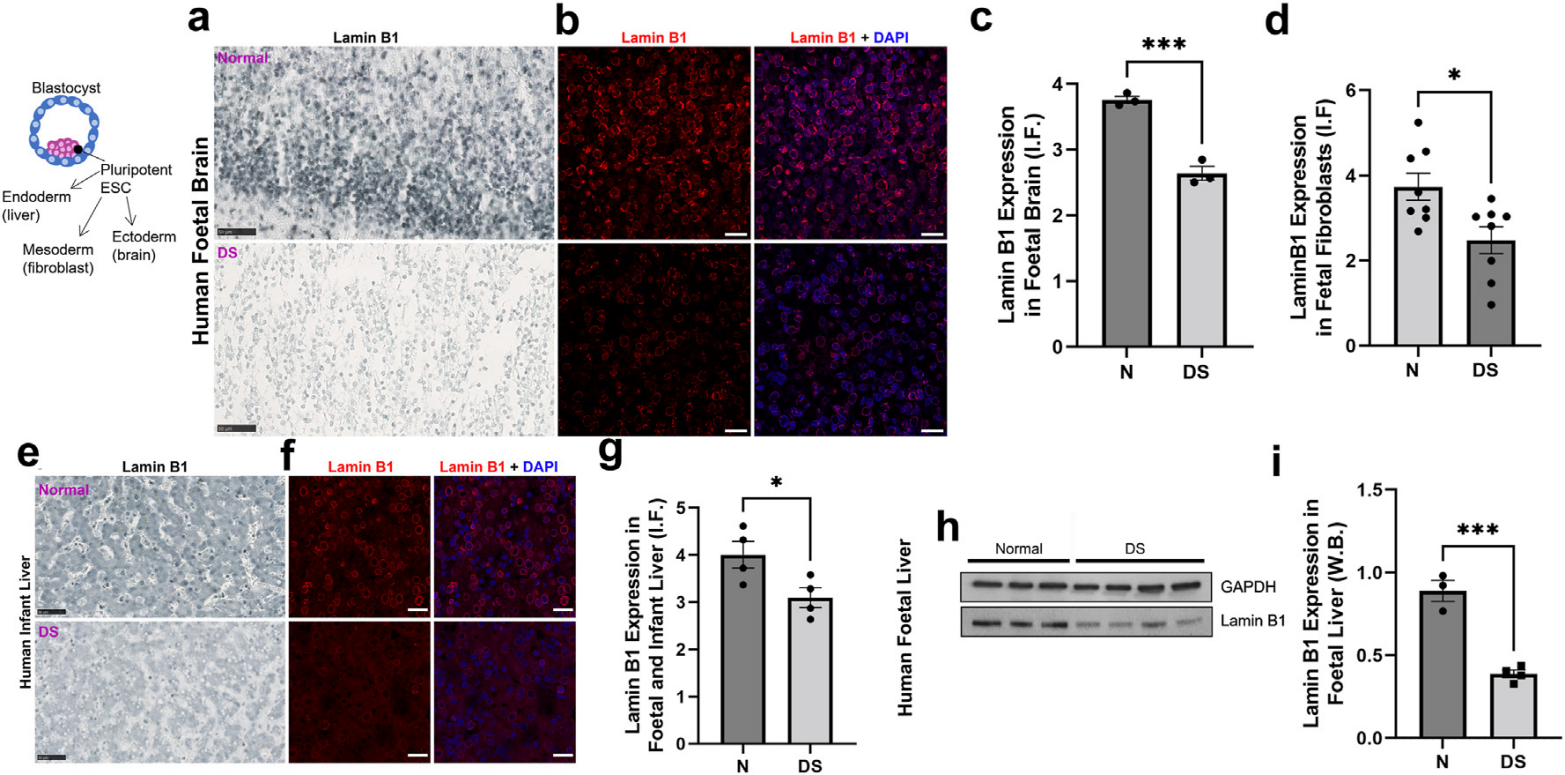
**Lamin B1 expression is reduced in T21 primary cells representative of all three germ layers. (a–c)** Gestational age- sex- and neuroanatomical region-matched (n = 3 pairs) DS and normal human foetal brain (frontal cortex) samples were analysed for Lamin B1 expression by bright immunohistochemistry **(a)** (scale bar: 50 μm) and immunofluorescence **(c)** (scale bar: 20 μm). Top row: normal sample. Bottom row: DS sample. Representative images are shown from 1 of the 3 pairs of samples. **(c)** Quantification of Lamin B1 expression, normalised to nuclear stain, was carried out on 5 immunofluorescence images for each sample. Each dot on the histogram represents the average from each individual brain, with n > 4000 nuclei analysed per sample (DS, n = 3; N, n = 3). Error bars: SEM. Statistical significance was calculated using an unpaired 2-tailed Student’s T-test (p = 0.0007). **(d)** Gestational-age- and passage-number- matched DS (n = 4) and normal (n = 4) human foetal fibroblasts were analysed by immunofluorescence for Lamin B1 expression, normalised to the number of nuclei. Each dot on the histogram represents the average of 3 images per coverslip and 2 coverslips per cell line were analysed. Error bars: SEM. Statistical significance was calculated using an unpaired 2-tailed Student’s T-test (p = 0.0142). **(e–g)** Age- and sex-matched DS and normal human foetal and infant liver samples from 18 to 20 gestational weeks (DS, n = 3; N, n = 3) and 11 months infants (DS, n = 1; N, n = 1) were analysed for Lamin B1 expression by bright immunohistochemistry **(e)** (scale bar: 50 μm) and immunofluorescence **(f)** (scale bar: 20 μm). Top row: normal sample. Bottom row: DS sample. Representative images are shown from the 11-month old infant pair of samples. **(g)** Quantification of Lamin B1 expression, normalised to nuclear stain, was carried out on 3–4 immunofluorescence images per each sample. Each dot on the histogram represents the average from each individual liver, with n > 5000 nuclei analysed per sample (DS, n = 4; N, n = 4). Error bars: SEM. Statistical significance was calculated using an unpaired 2-tailed Student’s T-test (p = 0.0415). **(h and i)** An independent set of age-matched DS and normal human foetal liver samples were compared by Western blotting and normalised to GAPDH. **(i)** Lamin B1 expression was quantified and plotted relative to GAPDH signal (DS, n = 4; N, n = 3). Error bars: SEM. Statistical significance was calculated using an unpaired 2-tailed Student’s T-test (p = 0.0004). For all parts: ∗ p <0.05, ∗∗∗ p <0.001.

A nearly 20-year accelerated IgG-glycan-profile age-clock (Figs. 1 and 2) is a reflection of general accelerated biological ageing, not limited to one tissue or organ system.^24^^,^^25^^,^^60^ As many diverse DS cell and tissue types show accelerated ageing, we hypothesised that these systemic progeroid features in DS could be caused by a cell autonomous genetic insult that originates before the tissue specification of the embryonic germ layers. To test this explanation, we first looked at Lamin B1 levels (whose drop is a marker of increased senescence^44^) in DS foetal tissues derived from all 3 embryonic germ layers, compared to age-matched euploid controls. Using a combination of immunohistochemistry, immunofluorescence and immunoblotting we show that Lamin B1 levels were significantly lower in foetal DS skin fibroblasts (mesodermal origin), foetal DS brain (ectodermal origin), and foetal and infant DS liver tissue (endodermal origin) (Fig. 7). Three pairs of neuro-anatomically matched foetal brain samples at gestational age 20–23 weeks analysed by immunohistochemistry and immunofluorescence showed a significant reduction in Lamin B1 expression in DS compared to controls (p = 0.0007, t-test, Fig. 7a–c). Four DS and four normal gestational age and passage number matched primary fibroblast cell lines were analysed by immunofluorescence and showed significantly decreased Lamin B1 staining for DS (p = 0.0142, t-test, Fig. 7d). A total of 8 foetal DS liver samples were compared to 7 age- and sex-matched control foetal liver samples by a combination of immunofluorescence (Fig. 7e–g) and Western blotting (Fig. 7h and i), depending on the nature of the tissue. Each method showed independently a significant reduction in Lamin B1 in the DS samples compared to controls (p = 0.0415 and p = 0.0004, respectively; t-test, Fig. 7g and i). Bearing in mind the caveat of a very small number of analysed samples (Fig. 7), when put together with the result in primary CRO1 PBMCs (Fig. 3h and i), these results indicate a systemic trend for reduction in Lamin B1 levels in DS, in multiple organ systems detectable from mid-gestation to childhood.

### Neuronal DNA damage, senescence and laminopathy can be corrected by genetic and chemical suppression of DYRK1A overdose in cerebral organoids

**Fig. 8:**
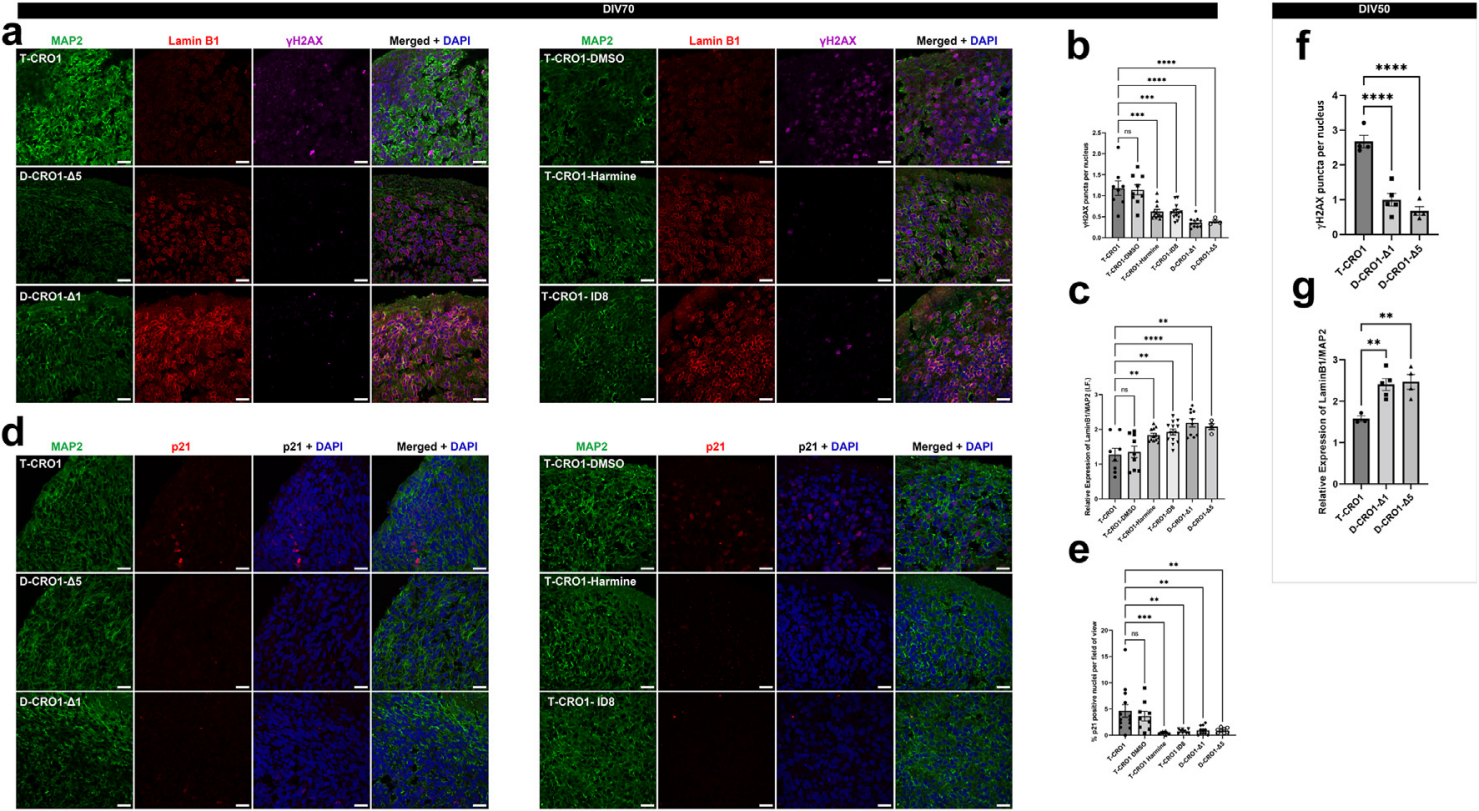
**γH2AX and Lamin B1 expression by immunofluorescence in DIV70 and DIV50 T-CRO1 and D-CRO1 cerebral organoids. (a)** Immunofluorescence images of cerebral organoid sections after 70 days of differentiation showing MAP2, Lamin B1 and γH2AX expression in T-CRO1 organoids alongside D-CRO1-Δ1 and D-CRO1-Δ5 organoids. T-CRO1 organoids were separately treated with DYRK1A inhibitors for 40 days (Harmine or ID8), while DMSO was used as a vehicle only control for each drug. Scale bar 20 μm. **(b)** Cerebral organoids with gene-dose reduction of *DYRK1A* by genome-editing or chemical DYRK1A kinase inhibition resulted in decreased γH2AX puncta per nucleus compared to T-CRO1 control organoids at DIV70. **(c)** Cerebral organoids with gene-dose reduction of *DYRK1A* by genome-editing or chemical DYRK1A kinase inhibition resulted in increased expression of Lamin B1 when compared to T-CRO1 organoids at DIV70. Lamin B1 was normalised to MAP2 expression in co-stained sections. **(d)** Immunofluorescence images of organoid sections after 70 days of differentiation showing MAP2 and p21 expression in T-CRO1 organoids alongside D-CRO1-Δ1 and D-CRO1-Δ5 organoids. T-CRO1 organoids were separately treated with DYRK1A inhibitors for 40 days (300 nM harmine or 500 nM ID8), while DMSO was used as a vehicle only control. Scale bar 20 μm. **(e)** Cerebral organoids with gene-dose reduction of *DYRK1A* by genome-editing or chemical DYRK1A kinase inhibition resulted in a decreased proportion of p21 positive cells compared to T-CRO1 organoids at DIV70. For **(b, c and e)** 3–5 organoids per genotype or condition were analysed (harmine: 300 nM, ID8: 10 μM), and 8–13 images containing a total of n > 1000 nuclei per cell line analysed. Graphs show mean ± SEM. Statistics were calculated by one-way ANOVA followed by Dunnett's correction for multiple comparisons. **(f and g)** Organoids were examined by immunostaining at DIV50. Organoids generated from both D-CRO1 lines showed **(f)** decreased γH2AX per nucleus compared to T-CRO1 organoids and **(g)** increased Lamin B1 expression compared to T-CRO1 organoids. Three organoids were analysed per cell line, and 3–5 images containing a total of n > 1000 nuclei per cell line analysed. Graphs show mean ± SEM. Statistics were calculated by one-way ANOVA followed by Dunnett's correction for multiple comparisons. For all parts: ∗∗p < 0.01, ∗∗∗p < 0.001, ∗∗∗∗p < 0.0001, ns = not significant.

**Supplementary Figure S9:**
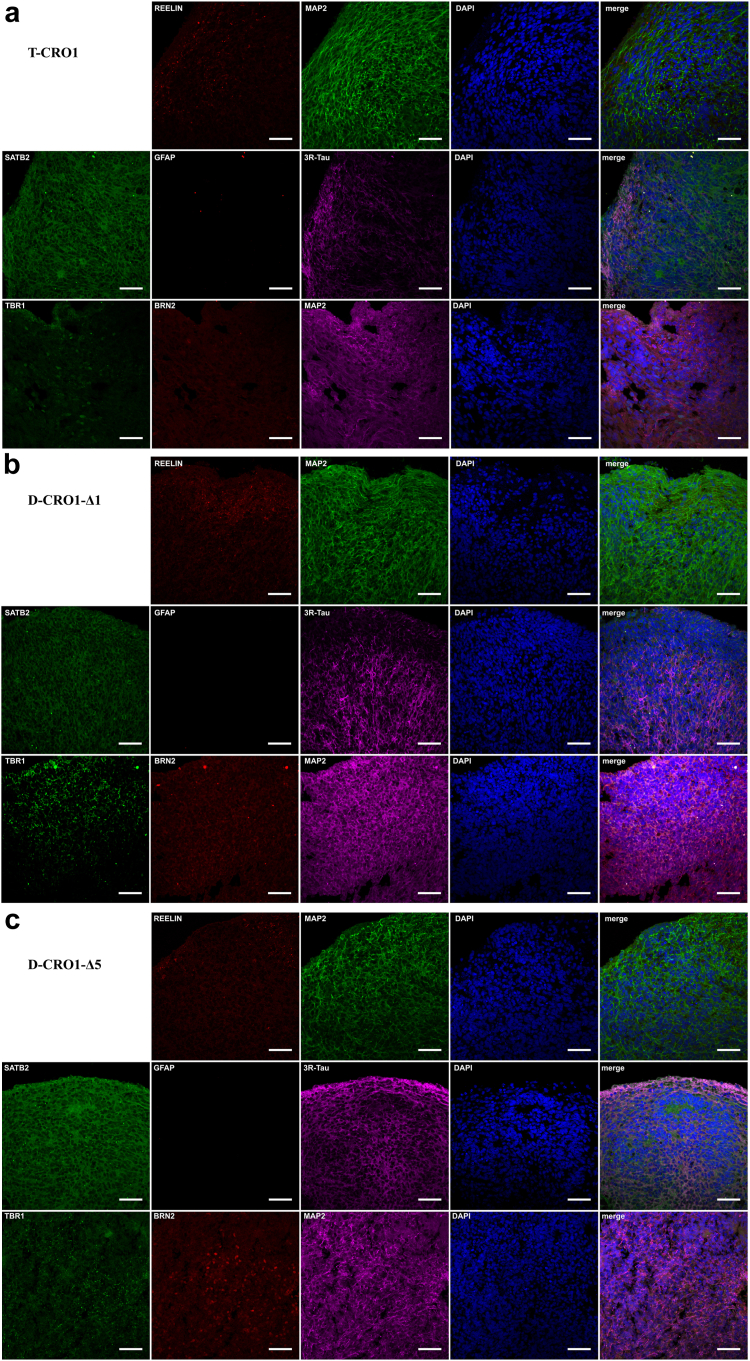


**Supplementary Figure S10:**
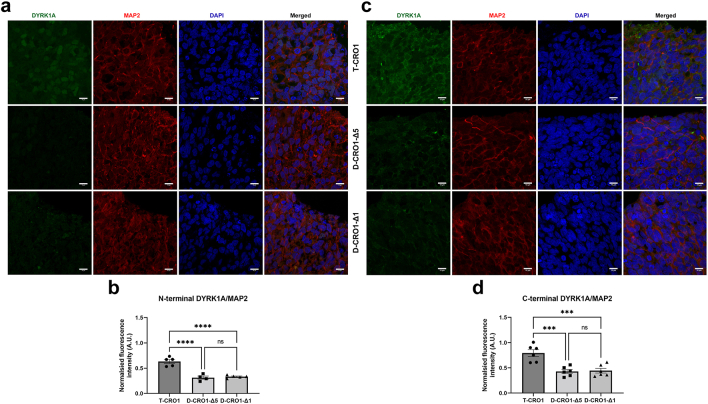


We next questioned if a link could be established between laminopathy in DS foetal tissues, and the laminopathy and DNA damage driven by DYRK1A, seen in hiPSC models. To this end, we generated cerebral organoids from the DYRK1A-dose-dissecting isogenic hiPSC models (Supplementary Figure S9), and tested if D-CRO1-Δ1 and D-CRO1-Δ5-derived organoids have significantly reduced DYRK1A levels in neurons (Supplementary Figure S10). The analysis shows that DYRK1A-dose-driven cellular ageing traits of Lamin B1 reduction and increase in γH2AX foci are also observed in cerebral organoids derived from hiPSCs (Fig. 8). We also added p21 (CDKN1A), a marker of age-dependent, DDR-induced senescence in neuronal lineages.^61^ The T-CRO1-derived organoids, compared to those derived from D-CRO1-Δ1 and D-CRO1-Δ5 hiPSCs showed markedly increased DSB numbers and p21 positive neurons and reduced Lamin B1 levels at both culturing timepoints examined (DIV50 and DIV70; Fig. 8). Treatment of T-CRO1 organoids with DYRK1A inhibitors for 40 days significantly reduced the DSB and p21 values, and restored the Lamin B1 levels (Fig. 8), demonstrating that this senescence and laminopathy-associated effect of DYRK1A can be, in principle, chemically counter-acted.

**Supplementary Figure S11:**
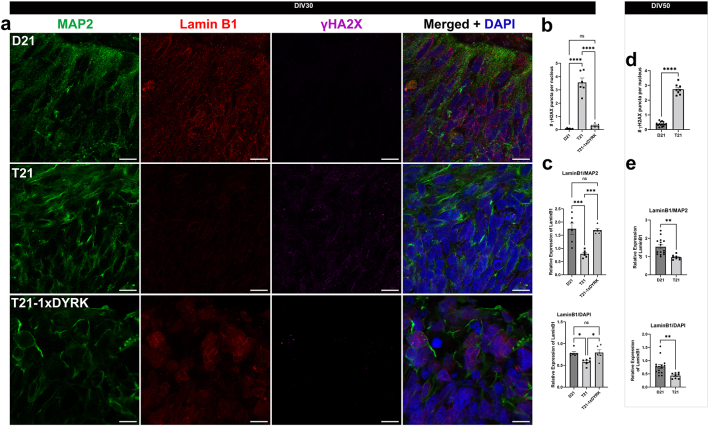


The level of DSBs and the level of Lamin B1 protein was further assessed in cerebral organoids grown from the full chromosome T21 isogenic model. For early organoids (at DIV30), the differences seen in the segmental duplication model CRO1, i.e., the number of γH2AX foci per nucleus was significantly higher in T21 compared to each D21 and T21-1xDYRK, were fully reproduced; in addition, the level of Lamin B1 relative to MAP2 (or relative to DAPI) was significantly lower in T21 compared to each D21 and T21-1xDYRK (Supplementary Figure S11a–S11c). A similar result was also obtained for the isogenic T21:D21 comparison in DIV50 organoids (Supplementary Figure S11d and S11e). The T21-1xDYRK and T21-0xDYRK organoids did not develop properly when cultured past DIV40 and DIV25, respectively, and cells started to die before acquiring the correct neuronal morphology (not shown). This suggests that other trisomic genes on chr21 render such organoids much more sensitive to dose imbalance of DYRK1A than in the case for CRO1 organoids.

### DS share many features with *LMNB1*-haploinsufficiency patients, including reduced cortical folding, reproducible in cerebral organoids, and correctable by *DYRK1A* gene dose ablation

**Fig. 9:**
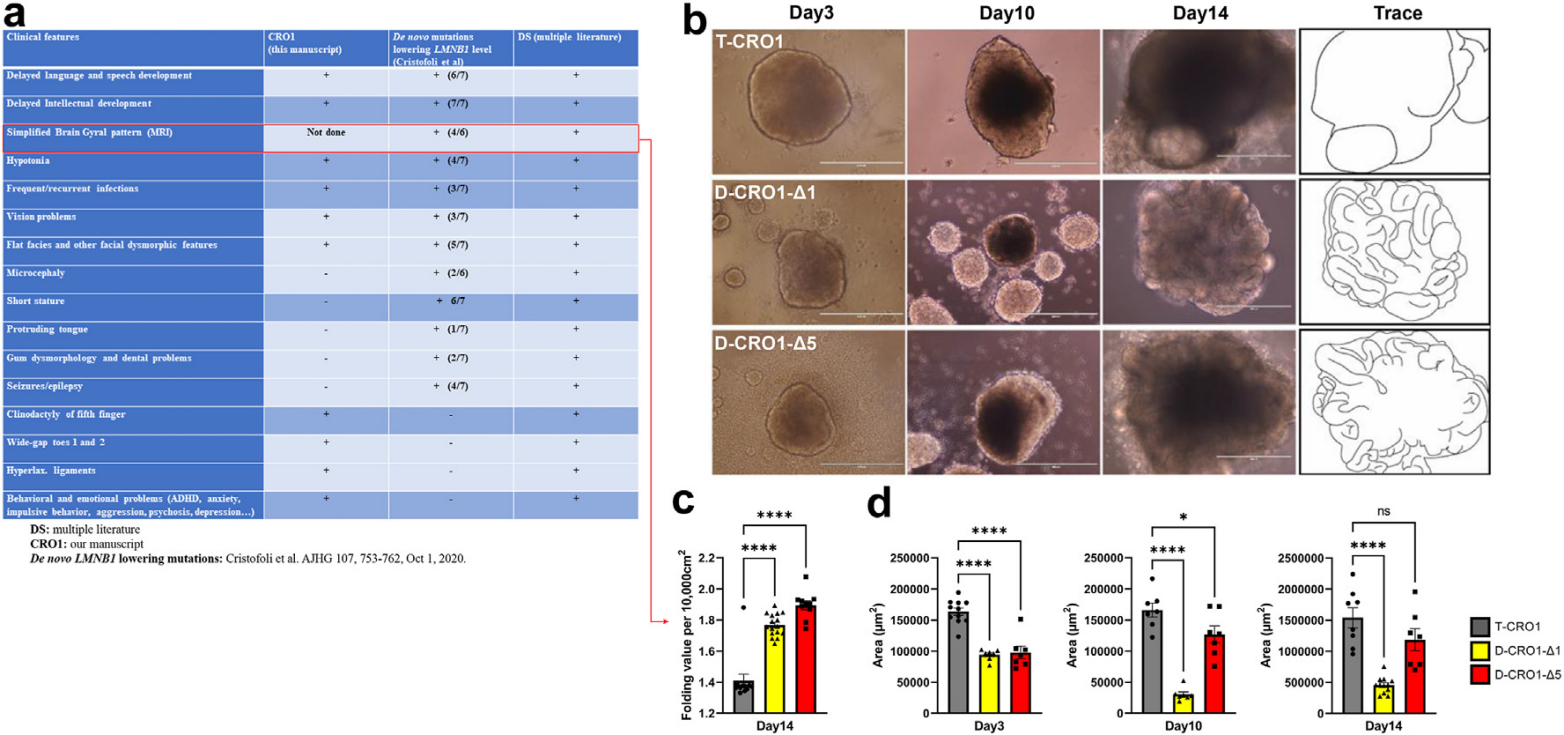
**T-CRO1 cerebral organoids show reduced cortical folding compared to D-CRO1 organoids, a feature shared by brains of *LMNB1***^**+/−**^**patients and DS foetuses. (a)** Comparison of the clinical features between the CRO1 child, patients with *LMNB1* mutations that lower Lamin B1 expression and individuals with DS, demonstrates a number of shared features. **(b)** Representative bright-field images of cerebral organoids generated in parallel. Day 3: Embryoid bodies (EBs) prior to neural induction. Starting cell number was consistent per line. Day 10: EBs after 4 days in neural induction medium. Clearing of the outer surface indicates the formation of neural ectoderm. Day 14: Cerebral organoids display radial organisation of neuroectoderm into organoid cortical folds. A simplified schematic outline is drawn to the right. Scale bars represent 400 μm. **(c)** Both D-CRO1 iPSC lines generated significantly more folded organoids than T-CRO1. The organoid folding value per 10,000 cm^2^ on image traces were calculated using ImageJ software of n = 11–16 images per cell line taken from two batches of cerebral organoids. Error bars: SEM. Statistical significance was calculated by one-way ANOVA followed by Dunnett’s correction for multiple comparisons (∗∗∗∗p < 0.0001). **(d)** Both D-CRO1 iPSC lines generated significantly smaller EB/organoids than T-CRO1. Average EB/organoid surface area on bright-field images were calculated using ImageJ software of n = 7–12 images per cell line taken from two batches of cerebral organoids. Error bars: SEM. Statistical significance was calculated by one-way ANOVA followed by Dunnett’s correction for multiple comparisons, p > 0.05 (ns), p ≤ 0.05 (∗), p ≤ 0.0001 (∗∗∗∗).

Taken together, the decrease in Lamin B1 in our *in vitro* models, fresh PBMCs from the segmental duplication case, and various DS foetal tissues lead us to hypothesise that DS contains a strong B-type laminopathy component. This explanation would predict shared pathological *in vivo* phenotypes between DS and developmental syndromes in which Lamin B1 levels are constitutionally decreased. Laminopathies represent a subset of progerias, with the most extremes of accelerated ageing phenotypes.^48^ While some are caused by splicing mutations in *LMNA* and *LMNC* genes (such as HGPS, resulting in a systemic drop in Lamin B1 levels), others caused by mutations in *LMNB* genes were connected with their over-expression (such as autosomal dominant leukodystrophy).^46^^,^^48^ Recently, however, several novel laminopathy cases with heterozygous null mutations in *LMNB1*, causing decreased Lamin B1 levels were described.^62^ We compared the detailed clinical features of these *LMNB1*-deficient laminopathies with DS (from literature) and CRO1 child (this work) phenotypes. This analysis revealed many shared features, including altered facial characteristics, hypotonia, microcephaly, delayed speech, learning and intellectual development, as well as simplified gyral folding in foetal brain by magnetic resonance imaging (MRI)^62^^,^^63^ (Fig. 9a). For ethical reasons the MRI could not be performed on the CRO1 child, but remarkably, the early cerebral organoid development showed much reduced “cortical” folding of the T-CRO1 organoids, compared to D-CRO1-Δ1 and D-CRO1-Δ5 organoids (p < 0.0001 for each compared to T-CRO1, ANOVA) (Fig. 9b and c), suggesting that the early correction of Lamin B1 levels correlates with improved gyrification during brain development. Of note, *DYRK1A* haploinsufficiency in D-CRO1-Δ1 and D-CRO1-Δ5 organoids did result in these two lines producing smaller organoids than T-CRO1 (Fig. 9d), underpinning the clinical reports of *DYRK1A* haploinsufficiency causing microcephaly.^64^ This re-confirms the previously reported successful modelling of microcephaly using cerebral organoids as a system.^65^ Our study adds here a new insight into how cerebral organoids could also model the MRI-phenotype of simplified gyrification/cortical folding.

## Discussion

Primary DS foetal neurons grown *in vitro* display increased reactive oxygen species (ROS) production, malfunctioning mitochondria, and an increased tendency to apoptosis, compared to euploid controls.^12^ Modelling DS neurons derived from iPSCs recapitulate some of these phenotypes: T21 neurons in 2D-neuronal cultures show malformed and malfunctioning mitochondria and increased DSB marks (γH2AX foci) compared to isogenic normal controls.^14^ The NPCs differentiated from T21 iPSCs, compared to their isogenic normal controls, display genome-wide disruption of lamina-associated domains and global chromatin accessibility changes consistent with nuclear architecture changes seen in senescent cells.^13^ These chromatin changes were not seen at undifferentiated pluripotent iPSC stage.^13^

However, in children and adults with DS, marks of premature senescence or accelerated ageing were seen in primary cells from organ systems other than CNS: immune cells,^66^ gingival cells, fibroblasts and lymphocytes.^15^, ^16^, ^17^, ^18^, ^19^, ^20^, ^21^, ^22^ The CpG-methylation defined “epigenetic clock” showed increased epigenetic ageing in primary blood cells from children^67^ and adults^23^ with DS.

Accelerated ageing in such diverse DS tissues and cell types could either be caused by the actions of different, tissue and cell-type specific overdosed chr21 genes via different mechanisms,^1^^,^^4^^,^^19^^,^^66^ or by an over-arching mechanism driving DS cells into a progeroid state very early in differentiation. No single chr21 gene action could so far explain the accelerated ageing in these diverse cell types originating from different embryonic layers, leaving room for alternative, no-specific-overdosed-protein mechanistic explanations, such as the amplified chromosomal instability, caused by a freely segregating supernumerary chromosome.^68^

We uncovered very significantly accelerated IgG glycosylation-based biological ageing (by 18.4–19.1 years on average) in DS (Fig. 1, Fig. 2, Fig. 3a, Supplementary Figure S2, Supplementary Tables S9–S13), independent of co-morbidities, reproducible in 3 different DS populations, and beginning in early childhood. The “amplified chromosomal instability” explanation would predict an accelerating rate of accumulation of ageing changes throughout lifetime.^68^ This is not observed in our data (Fig. 2, Supplementary Table S14), where the slope of glycan change with age does not differ between DS and controls for any of the three populations. Surprisingly, the IgG glycan profile of a 2-year-old child with DS caused by a segmental duplication of only 31 chr21 genes (CRO1) had values similar to those of age-matched children with DS, outside the euploid children’s range (Fig. 3a), suggesting that one or more of these genes, at trisomic dose, could be sufficient to accelerate general biological ageing, not needing a freely segregating supernumerary chromosome.

As there are signs of ageing and senescence in multiple tissues of DS that originate from both neuroectodermal and mesodermal embryonic layers, we hypothesised that modelling in pluripotent iPSCs, before the differentiation commitment to specific embryonal lineages, could be used to uncover such mechanisms. The reprogramming process of iPSC generation is known to erase all epigenetic marks of ageing that might have been present in primary cells used for reprogramming,^69^ leaving a “tabula rasa” cellular system, in which newly emerging cellular changes underpinning developmental abnormalities may occur. However, due to their fast proliferation rate, human iPSCs in general also show a much greater presence of DSBs compared to the primary cells they were derived from.^49^ We examined whether the presence of supernumerary genes from chr21 could further affect this basic level of endogenous, iPSC-generated DSBs.

We therefore generated iPSCs from a child that bears only 31 genes in trisomic overdose, and generated two isogenic control iPSC lines (using CRISPR/Cas9) in which the trisomic dose of all overdosed 31 genes was corrected to disomy, with an additional, accidental decrease of *DYRK1A* copy dose to monosomy (Fig. 3, Supplementary Figure S6). This isogenic system proved that trisomic overdose of this chromosomal segment caused an increase in nuclear γH2AX foci, likely reflecting an increase in unrepaired DSBs in iPSC nuclei (Fig. 4, Supplementary Figure S7). As DYRK1A was previously seen interacting with and phosphorylating DNA repair proteins,^55^, ^56^, ^57^ we inhibited this phosphorylation activity using two selective chemical inhibitors. Each of these inhibitors corrected the number of γH2AX foci nearly to the same level as the CRISPR/Cas9 editing (Fig. 4), showing that DYRK1A overdose is the likely main driver of this phenotype, but not completely excluding the potential additional, weaker contribution of other genes in the CRO1 duplicated segment. The same result was reproduced when *DYRK1A* dose (using gene-editing), or its kinase activity (using two chemical inhibitors) were reduced in the full chr21 trisomy iPSC model and compared to its isogenic disomic control (Figs. 4 and 5, Supplementary Figure S8). Moreover, in the iPSC line trisomic for the entire chr21, but lacking functional endogenous DYRK1A kinase, exogenous over-expression of wild-type, but not kinase-dead, DYRK1A, increased the number of γH2AX foci (Fig. 5), proving that dose and kinase activity of DYRK1A are both necessary and sufficient to cause the state of increased DNA damage in pluripotent, undifferentiated iPSCs, that are trisomic for the entire chr21.

Increased γH2AX foci and unrepaired DSBs are found in primary fibroblasts from children and adults with DS,^17^ as well as DS blood cells.^19^ Nuclei of primary fibroblasts from classical laminopathy-associated progeria (HGPS) have increased γH2AX foci, accompanied by a decreased Lamin B1 level,^45^ a well-established early marker of senescence.^44^ We observed the same two phenotypes in freshly isolated nucleated blood cells from the CRO1 child (Fig. 3f–i). T-CRO1 and full chromosome T21 undifferentiated iPSCs showed a significant decrease in Lamin B1, that can be corrected to normal by depletion of *DYRK1A* copy number (Fig. 6). Loss of Lamin B1 in senescence shares common features with HGPS in the destabilisation of nuclear lamina,^70^ and causes redistribution of heterochromatin domains and disturbance in the distribution of lamina-associated domains (LADs).^71^ Similar heterochromatin re-structuring (chromosomal introversion), along with the redistribution of LADs, as well as a significant drop in Lamin B1 level was recently reported in NPCs derived from T21 iPSCs, but the cause of this perturbation remained elusive.^13^ These authors did not find a significant decrease in Lamin B1 in undifferentiated iPSCs, however, in their Supplementary Figure S3, we noticed that T21 iPSCs were showing a strong decreasing trend for Lamin B1, compared to isogenic controls, just short of statistical significance,^13^ which hints at the Lamin B1 decrease reproducibility in other hiPSC models, unrelated to our two isogenic hiPSC systems. We cannot exclude that differentiation into NPCs exacerbates these effects, and/or triggers additional mechanisms operating only at NPC stage. Importantly, our data show that neurons in cerebral organoids derived from the CRO1 iPSC model trisomic only for the 31 genes including *DYRK1A*, showed decreased Lamin B1 and increased γH2AX foci, compared to controls disomic for the 31 genes, and monosomic for *DYRK1A* (Fig. 8, Supplementary Figures S9 and S10). Finally, a prolonged culturing of organoids with media containing either of the two chemical inhibitors of DYRK1A restored to normal both γH2AX foci and Lamin B1 levels (Fig. 8). This opens up a theoretical possibility of early therapeutic interventions quenching the action of DYRK1A, in order to restore the levels of Lamin B1, and stop further development of laminopathy-associated phenotypes.

While HGPS is caused by a *LMNA/C* mutation driving a splicing aberration, it is also accompanied by a strong decrease in Lamin B1 levels.^45^ Though performed on a very small number of samples, our analysis of Lamin B1 expression in DS vs euploid age-matched foetal and infant tissues, suggests a widespread decrease in Lamin B1 levels in DS cells representing all three embryonic layers (Fig. 7). Our conclusions on Lamin B1 levels in foetal and infant DS and control tissues are limited due to a very small number of samples (n = 21 in total), and were included as an attempt to validate the iPSC-modelling results in primary tissues. The extent to which the drop in Lamin B1 levels is visible in DS tissues could also be subject to compensatory effects driven by the cell proliferation status at the particular developmental stage of the tissue examined, oxidative stress status, and/or action of other, tissue specific genes de-regulated by T21. Recently, the first cases of syndromes caused by loss-of-function mutations in *LMNB1* were described.^62^ Comparison of their clinical features with DS reveals a spectrum of shared phenotypes, many of which were also shared with the CRO1 segmental duplication case we described here (Fig. 9a). We highlight that a simplified pattern of gyral sulci (reduced cortical folding) is shared between DS foeti^63^ and *LMNB1*^+/−^ patients,^62^ and that our CRO1 cerebral organoid model shows a reduction in cortical folding, that is restored by the correction of the segmental trisomy and depletion of *DYRK1A* copy number (Fig. 9b). Examination of published literature, driven by the Lamin B1-laminopathy-hypothesis for DS, reveals that indeed, multiple, major DS neurodevelopmental abnormalities have been mirrored in *LMNB1* knockdown models: reduced dendritic spine density,^72^ Tau redistribution in dendrites (neurons from hiPSCs),^73^ decrease in synaptic puncta^74^ (neurons from hiPSCs), are all seen in *Lmnb1*-K.O. mice.^75^ Gliogenic shift seen in DS brains and mouse models^76^ and iPSC neurospheres^77^ can also be caused by the constitutional loss of *LMNB1*.^78^ Neuronal migration defects (seen in neurons from DS hiPSCs)^79^ are mimicked by *Lmnb1*-null mice,^80^ that also show reduced neuronal numbers in the cortex.

All these observations put together compel us to propose that: (i) cell-autonomous excess un-repaired DNA damage-associated progeroid status is a significant constituent component of DS, (ii) trisomy of *DYRK1A* causes this (not excluding contributions from other genes in specific cell types), (iii) this can drive a reduction of Lamin B1 levels in a variety of cell types during development, (iv) the above pathogenetic processes can be reversed by an exogenous intervention correcting the excess kinase activity of DYRK1A.

Recently, clinical trials using the DYRK1A inhibitor EGCG have been conducted in young adults with DS^81^ (see also NCT02432716 and NCT01699711). Our data suggests that inhibition of DYRK1A-overdose effects, if applied during development, might ameliorate important neurodevelopmental phenotypes of DS. This concept should be approached carefully, as constitutional *DYRK1A* haploinsufficiency causes microcephaly.^64^ It therefore remains a challenge to find modalities of fine-tuning this inhibition, in order to ameliorate, and not worsen, the neurological phenotypes of DS. In mature neurons derived from iPSCs by direct differentiation, a significant drop in Lamin B1 protein expression can be seen for T21 and familial early-onset Alzheimer’s disease (searching specifically for *LMNB1* in SuppTables 1A-C^82^), which aligns with our models and interpretation, but also with some of our unpublished data suggesting an additional role of Aβ aggregate toxicity on the degree of senescence of mature neurons. This phenomenon demands a much deeper analysis, as it suggests that, depending on when the corrective chemical inhibition is administered, other gene over-actions would need to be inhibited in addition to the partial inhibition of DYRK1A. In conclusion, our findings suggest that DYRK1A overdose triggers the DNA damage-borne stress signal causing cellular senescence, which consequently drives a tendency towards reduced Lamin B1 levels, but cannot exclude additional direct DYRK1A overdose effects on levels or function of Lamin B1 protein. More experiments are needed to fully unravel these questions.

## Contributors

AM, IA, GL, and DN conceptualised the study.

AStr, CF, IB, TS, and MT lead the clinical teams providing the samples and co-morbidities stratification.

AC, JJ, MPB, ASla, HD, and JK performed glycan analysis.

FV and AF conducted statistical analysis for glycan experiments.

VB, SH, CS, HD’S, MK, LjO, BK, ŽK, and IK obtained the primary samples.

JG, NO’B, DP, DM, AB, AP, VH, and FD processed samples.

AM, GG, IA, RB, YYJ, HYC, JNF, and YHL performed iPSC, CRISPR/Cas9, and organoid experiments.

SH, NRD, and SdlL provided constructs and design of the specific CRISPR/Cas9 tools.

IA, AM, GG, and LG performed immunofluorescence image analysis.

AM, IA, GG, FV, DK, VB, SdlL, AStr, GL, JK, and DN interpreted the results.

AM, GG, AC, FV, IA, JK, and DN wrote the manuscript.

GL, SdlL, and AStr provided detailed manuscript revisions.

AM, JK, IA, and DN verified the underlying data.

All other authors reviewed and approved the final version of the manuscript.

## Data sharing statement

The data generated in this study has largely been included in the manuscript and supplementary data files. Any additional datasets generated and analysed in this study are available from the corresponding authors on reasonable request.

## Declaration of interests

GL is the founder and owner of Genos Ltd., a private research organisation that specialises in high-throughput glycomic analyses and has several patents in this field and is also a shareholder in GlycanAge Ltd., a company that sells the GlycanAge test of biological age. AC, FV, JJ, MPB, ASla, HD, AF, DP and JK are employees of Genos Ltd. AStr has served on the Advisory Boards of AC Immune and ProMIS Neuroscience, and is a past president of the Trisomy21 Research Society. TS is the scientific co-founder and a shareholder of Zoe Ltd.

## Appendix A Supplementary data

Supplementary Tables S1–S15

Supplementary Discussion

Caption for Supplementary Material

Supplementary Western Blots
