# Supplementary material for: Dose imbalance of DYRK1A kinase causes systemic progeroid status in Down syndrome by increasing the un-repaired DNA damage and reducing LaminB1 levels
Source: eBioMedicine. 2023 Jul 12;94:104692. doi: 10.1016/j.ebiom.2023.104692 (PMC10435767; doi:10.1016/j.ebiom.2023.104692)
Supplement: Supplementary Tables S1–S15 [file mmc1.docx]

**Supplementary Tables**

**Supplementary Table 1. Human Foetal Fibroblasts**

| **Cell line** | **Code** | **Sex** | **Gestational age** | **Diagnosis** | **Source** |
| --- | --- | --- | --- | --- | --- |
| DS2 | RA03300F | F | 18 weeks | 47XX, +21 | Galliera Genetic Bank, Genova, Italy |
| DS3 | E-A0363M | M | 19 weeks | 47XY, +21 |  |
| DS4 | N-P0047M | M | 18 weeks | 47XY, +21 |  |
| N5 | DA05370F | F | 18 weeks | 46XX |  |
| N6 | CR07265F | F | 12+2 weeks | 46XX |  |
| N7 | BE06633M | M | 20 weeks | 46XY |  |
| N8 | HM07148M | M | 14+4 weeks | 46XY |  |

**Supplementary Table 2. Primer sequences**

| **Oligo name** | **Sequence 5’-3’** | **Product size (bp)** | **Genome region** |
| --- | --- | --- | --- |
| DYRK1A_F | CCGTATTCGTAGTCTAATGTTG | 528 | chr21:37493131-37493153 |
| DYRK1A_R | CTAACTGTTCTCCTTCATGAGTAG |  |  |
| OT1-F | CTAAGTGTCACTGGCTGCTC | 255 | chr1:147750425-147750447 |
| OT1-R | CAATTTCACATGGGTGCAG |  |  |
| OT2-F | GAGTCTATCTGCAGAGGCAAC | 307 | chr3:11965449-11965471 |
| OT2-R | GACCCCATGCTTAATGTTG |  |  |
| OT3-F | GTGAGTGGATGGAAACTGAG | 265 | chr10:69913547-69913569 |
| OT3-R | GATGATGGGGAGGTGAGAG |  |  |
| OT4-F | GTACAACTTGGCTTCTCTTGAG | 236 | chr2:140140711-140140733 |
| OT4-R | CAATAGGTCCTGGCTTCTG |  |  |
| OT5-F | CATGGCTGGTGACCTTACAC | 266 | chr2:140140711-140140733 |
| OT5-R | CTTCTCTCCCACTCCCTTG |  |  |
| OT6-F | CAGCACTCTCCCAGGTTAG | 273 | chr14:69564944-69564966 |
| OT6-R | GATGGGTAAAATGACAGAATGC |  |  |
| OT7-F | GTTTGTGCAGGAGGGTATAGAC | 185 | chr1:33282935-33282957 |
| OT7-R | CTGATATTGGCAAACACGTG |  |  |
| OT8-F | GTAGAGGCTGTGTGGAAATG | 295 | chr9:17932113-17932135 |
| OT8-R | CAACAGATGTGGCTTTTGAC |  |  |
| OT9-F | CACAGAAGTAGGCAGTCAATG | 254 | chr11:115190613-115190635 |
| OT9-R | CTGTTTTCTCTGTGGTAGCG |  |  |
| OT10-F | GGAAGATTCTTTGTTTTGGAG | 327 | chr1:189190111-189190133 |
| OT10-R | CTGTGAGCTGAGTGGAGATG |  |  |

**Supplementary Table 3: Primers used for qPCR**

| **Sequence (5’-3’)** | **Name/Gene** | **Tm (°C)** | **Product size (bp)** |  |
| --- | --- | --- | --- | --- |
| GTGGTGCCAATGAGGTAGAT | Niz066_*DYRK1A*-F | 61.4 | 85 | Used for T21 and T21-0xDYRK iPSC qRT-PCR |
| GGTGCTTGGTCAAGAATAT | Niz067_*DYRK1A*-R | 59.6 |  |  |
| GAAGAACTGATGGAAAGCC | Niz068_*LMNB1*-F | 58.8 | 84 |  |
| CAAACATGCTCTAGACTCTTTCTG | Niz069_*LMNB1*-R | 61 |  |  |
| CATTTCCTGGTATGACAACG | Niz064_*GAPDH*-F | 60.4 | 83 |  |
| GTCTTACTCCTTGGAGGCC | Niz065_*GAPDH*-R | 60.3 |  |  |
|  |  |  |  |  |
| AAGAAGCGAAGACACCAACAG | DNO642_h*DYRK1A*-for | 63.3 | 139 | Used to T21 and D21 iPSC qRT-PCR |
| TTTCGTAACGATCCATCCACTTT | DNO643_h*DYRK1A*-rev | 65.3 |  |  |
| GGACTTCGAGCAAGAGATGG | DNO570_*B-actin*_F | 63.9 | 329 |  |
| AGCACTGTGTTGGCGTACAG | DNO571_ *B-actin*_R | 64.0 |  |  |

**Supplementary Table 4. Primary antibodies:**

| **antibody** | **clone** | **species** | **source** | **Cat.No** | **RRID** | **dilution** | | **Note** |
| --- | --- | --- | --- | --- | --- | --- | --- | --- |
|  |  |  |  |  |  | IHC/IF | WB |  |
| Beta-Actin |  | mouse IgG | Sigma | A5441 | AB_476744 | N/A | 1:10000 |  |
| Brachyury | D2Z3J | rabbit IgG | Cell Signaling | #81694 | AB_2799983 | 1:600 | N/A |  |
| DYRK1A-N-terminal |  | rabbit IgG | Abcam | ab65220 | AB_1140733 | 1:200 | 1:1000 |  |
| DYRK1A-C-terminal |  | rabbit IgG | Abcam | ab180910 | N/A | 1:200 | 1:1000 |  |
| FOXA2 | EPR4466 | rabbit IgG | Abcam | ab108422 | AB_11157157 | 1:200 | N/A |  |
| GFP |  | chicken IgY | Abcam | ab13970 | AB_300798 | 1:1000 | N/A |  |
| GAPDH | 6C5 | mouse IgG | Abcam | ab8245 | AB_2107448 | N/A | 1:1000 |  |
| Histone H3 | 96C10 | mouse IgG1 | Cell Signaling | 3638S | AB_1642229 | N/A | 1:1000 |  |
| Lamin B1 | L-5 | mouse IgG1 | ThermoFisher | 33-2000 | AB_2533106 | 1:200  iPSCs | 1:2000 | MeOH fixation for Fig 6  WB: Fig 7H |
| Lamin B1 |  | rabbit IgG1 | Abcam | ab16048 | AB_443298 | 1:200  Primary cells and tissues (PBMCs, fibroblasts, brain & liver),  Organoid sections | 1:1,000  iPSC lysates and human foetal brain | 4% PFA fixation for: Figs: 3, 7A-G, 8 and S10  WB: Fig:6C (D21/T21) and 6G (CRO1 series) |
| MAP2 |  | chicken IgY | Abcam | ab5392 | AB_2138153 | 1:1000 | N/A |  |
| Nestin | 196908 | mouse IgG1 | Abcam | ab6320 | AB_308832 | 1:200 | N/A |  |
| OCT4 |  | rabbit IgG | Cell Signaling | #2750 | AB_823583 | 1:400 | N/A |  |
| p21 | DCS60 | mouse IgG2a | Cell Signaling | #2946 | AB_2260325 | 1:100 | N/A |  |
| phospho-Histone H2A.X (Ser139) [γH2AX] | JBW301 | mouse IgG1 | Merck (Sigma) | 05-636 | AB_309864 | 1:200 | 1:1000 |  |
| RNF169 |  | rabbit IgG | Abcam | ab220993 | N/A | 1:200 | N/A |  |
| SMA |  | rabbit IgG | Abcam | ab5694 | AB_2223021 | 1:200 | N/A |  |
| SSEA |  | mouse IgG3 | Cell Signaling | #4755 | AB_1264259 | 1:500 | N/A |  |
| TRA 1-60 |  | mouse IgM | Cell Signaling | #4746 | AB_2119059 | 1:1000 | N/A |  |
| TRA 1-81 |  | mouse IgM | Cell Signaling | #4745 | AB_2119060 | 1:250 | N/A |  |
| 53BP1 |  | rabbit IgG | Bethyl | A300-273A | AB_185521 | 1:200 | N/A |  |

**Supplementary Table 5. Secondary antibodies:**

| **antibody** | **conjugate** | **source** | **Cat.No** | **RRID** | **dilution** |
| --- | --- | --- | --- | --- | --- |
| Donkey anti-Mouse IgG (H + L) | Alexa Fluor 488 | ThermoFisher Scientific | A-21202 | AB_141607 | 1:1000 |
| Donkey anti-Mouse IgG (H + L) | Alexa Fluor 555 | ThermoFisher Scientific | A-31570 | AB_2536180 | 1:1000 |
| Donkey anti-Mouse IgG (H + L) | Alexa Fluor 647 | ThermoFisher Scientific | A-31571 | AB_162542 | 1:500 |
| Donkey anti-Rabbit IgG (H + L) | Alexa Fluor 488 | ThermoFisher Scientific | A-21206 | AB_2535792 | 1:1000 |
| Donkey anti-Rabbit IgG (H + L) | Alexa Fluor 555 | ThermoFisher Scientific | A-31572 | AB_162543 | 1:1000 |
| Donkey anti-Rabbit IgG (H + L) | Alexa Fluor 647 | ThermoFisher Scientific | A-31573 | AB_2536183 | 1:500 |
| Goat anti-Mouse IgM | Alexa Fluor 568 | ThermoFisher Scientific | A-21043 | AB_2535712 | 1:500 |
| Goat anti-Chicken IgY (H + L) | Alexa Fluor 488 | ThermoFisher Scientific | A-11039 | AB_2534096 | 1:1000 |
| Goat anti-Chicken IgY (H + L) | Alexa Fluor 633 | ThermoFisher Scientific | A-21103 | AB_2535756 | 1:500 |
| Goat anti-Rabbit IgG (H + L) | HRP | Abcam | ab97051 | AB_10679369 | 1:10000 |
| Goat anti-Mouse IgG (H + L) | HRP | Abcam | ab97023 | AB_10679675 | 1:10000 |
| VECTASTAIN ABC HRP Kit | HRP | Vector | PK-4001 | AB_2336810 | 1:200 |

**Supplementary Table 6: Primary human tissues**

| **Sample ID** | **Organ** | **Sex** | **Age** | **Diagnosis** | **Source** |
| --- | --- | --- | --- | --- | --- |
| Foetal Liver 1 | Liver | M | 19-20 gestation weeks | Normal | Department of Pathology, University of Zagreb, Croatia |
| Foetal Liver 2 | Liver | M | 19-20 gestation weeks | Normal |  |
| Foetal Liver 3 | Liver | M | 18-19 gestation weeks | Normal |  |
| Infant Liver 1 | Liver | F | 11 months | Normal |  |
| Foetal Liver 4 | Liver | M | 19-20 gestation weeks | DS |  |
| Foetal Liver 5 | Liver | M | 18-19 gestation weeks | DS |  |
| Foetal Liver 6 | Liver | M | 19 gestation weeks | DS |  |
| Infant Liver 2 | Liver | F | 11 months | DS |  |
| Foetal Brain 1 | Brain |  | 20 gestation weeks | Normal | Brain Bank of the Croatian Institute for Brain Research (CIBR) |
| Foetal Brain 2 | Brain |  | 20 gestation weeks | Normal |  |
| Foetal Brain 3 | Brain |  | 21 gestation weeks | Normal |  |
| Foetal Brain 4 | Brain |  | 20 gestation weeks | DS |  |
| Foetal Brain 5 | Brain |  | 21 gestation weeks | DS |  |
| Foetal Brain 6 | Brain |  | 21-23 gestation weeks | DS |  |
| Foetal Liver 7 | Liver (WB) | M | 21.4 gestation weeks | Normal | UK MRC foetal tissue bank |
| Foetal Liver 8 | Liver (WB) | F | 20 gestation weeks | Normal |  |
| Foetal Liver 9 | Liver (WB) | F | 20.4 gestation weeks | Normal |  |
| Foetal Liver 10 | Liver (WB) | M | 22 gestation weeks | DS | Galliera Genetic Bank, Genova, Italy |
| Foetal Liver 11 | Liver (WB) | F | 23 gestation weeks | DS |  |
| Foetal Liver 12 | Liver (WB) | F | 20 gestation weeks | DS |  |
| Foetal Liver 13 | Liver (WB) | F | 20 gestation weeks | DS |  |

**Supplementary Table 7. Chemicals:**

| **Chemical** | **Source** | **Cat.No** | **Dilution/final concentration** |
| --- | --- | --- | --- |
| Acetone | Sigma | 32201-2.5L-M | undiluted |
| BCA assay kit | Thermo Fisher | 23227 | N/A |
| Comet Assay Kit | Abcam | ab238544 | N/A |
| Dako Fluorescence Medium | DAKO | S3023 | N/A |
| DAPI | Sigma | D9542 | 1:8000 |
| DMSO | Sigma | D2650 | N/A |
| Harmine | Sigma | 286044 | 300nM |
| H_2_SO_4_ | Rapid Electronics | 522-7570 | 0.4N |
| ID-8 | Adipogen | AG-CRI-3655 | 10µM and 500nM |
| Methanol | EMPLURA® | 8.22283.2500 | N/A |
| Paraformaldehyde | Sigma | P6148 | 4% |
| Sodium Butyrate | Sigma | 303410 | 5mM |
| Trichloroacetic acid | Fisher Scientific | BP555-250 | N/A |

|  | **FRA, Control**, N = 109 | | **FRA, DS**, N = 98 | | **ITA, Control**, N = 53 | | **ITA, DS**, N = 57 | | **UK, Control**, N = 42 | | **UK, DS**, N = 53 | |
| --- | --- | --- | --- | --- | --- | --- | --- | --- | --- | --- | --- | --- |
| **Characteristic** | **F**, N = 54 | **M**, N = 55 | **F**, N = 51 | **M**, N = 47 | **F**, N = 25 | **M**, N = 28 | **F**, N = 24 | **M**, N = 33 | **F**, N = 17 | **M**, N = 25 | **F**, N = 22 | **M**, N = 31 |
| **Age** | 44 (41 – 51)^1^ | 46 (43 – 53)^1^ | 44 (41 – 52)^1^ | 47 (44 – 54)^1^ | 39 (29 – 45)^1^ | 38 (32 – 46)^1^ | 41 (31 – 49)^1^ | 33 (29 – 43)^1^ | 46 (28 – 52)^1^ | 49 (40 – 55)^1^ | 48 (44 – 52)^1^ | 49 (42 – 57)^1^ |
| **Autoimmunity** | 0 (NA) | 0 (NA) | 24 (47%) | 10 (21%) | 0 (NA) | 0 (NA) | 9 (39%) | 13 (39%) | 0 (NA) | 0 (NA) | 14 (64%) | 11 (37%) |
| **ThyroidDis** | 0 (NA) | 0 (NA) | 17 (33%) | 6 (13%) | 0 (NA) | 0 (NA) | 10 (43%) | 14 (42%) | 0 (NA) | 0 (NA) | 13 (59%) | 11 (37%) |
| **Dementia** | 0 (NA) | 0 (NA) | 10 (20%) | 10 (21%) | 0 (NA) | 0 (NA) | 0 (NA) | 0 (NA) | 0 (NA) | 0 (NA) | 6 (27%) | 7 (23%) |
| **FreqInfections** | 0 (NA) | 0 (NA) | 6 (12%) | 9 (19%) | 0 (NA) | 0 (NA) | 6 (26%) | 10 (30%) | 0 (NA) | 0 (NA) | 3 (14%) | 4 (15%) |
| ^1^Median (IQR) | | | | | | | | | | | | |

**Supplementary Table 8. Sex-disaggregated data on characteristics of Down syndrome cohorts and healthy controls.**

**Supplementary Table 9: Comparison of directly measured IgG glycan traits and derived IgG glycan traits between persons with Down syndrome (DS) and healthy controls from the general population.**

| **Directly measured IgG glycan traits** | | | | | | | | | | | | | | | | | | | |
| --- | --- | --- | --- | --- | --- | --- | --- | --- | --- | --- | --- | --- | --- | --- | --- | --- | --- | --- | --- |
|  | **FRA** | | | | | **ITA** | | | | | **UK** | | | | | **meta** | | | |
| **Glycan** | **effect** | **DF** | **SE** | **p.val** | **p.adj** | **effect** | **DF** | **SE** | **p.val** | **p.adj** | **effect** | **DF** | **SE** | **p.val** | **p.adj** | **effect** | **SE** | **p.val** | **p.adj** |
| **GP1** | **0.345** | **198** | **0.120** | **3.747E-03** | **4.371E-03** | **0.171** | **101** | **0.158** | **2.616E-01** | **2.930E-01** | **0.576** | **86** | **0.175** | **8.050E-04** | **1.252E-03** | **0.349** | **0.084** | **3.360E-05** | **4.091E-05** |
| **GP2** | **-0.867** | **198** | **0.118** | **1.938E-12** | **4.933E-12** | **-0.372** | **101** | **0.172** | **2.548E-02** | **3.397E-02** | **-0.507** | **86** | **0.190** | **5.948E-03** | **7.570E-03** | **-0.665** | **0.087** | **1.703E-14** | **2.980E-14** |
| **GP3** | **0.650** | **198** | **0.111** | **8.559E-09** | **1.410E-08** | **0.579** | **101** | **0.161** | **2.759E-04** | **5.518E-04** | **0.818** | **86** | **0.167** | **1.315E-06** | **2.629E-06** | **0.671** | **0.080** | **5.452E-17** | **1.018E-16** |
| **GP4** | **0.873** | **198** | **0.104** | **2.050E-15** | **9.568E-15** | **0.645** | **101** | **0.156** | **3.208E-05** | **1.210E-04** | **0.973** | **86** | **0.156** | **2.602E-09** | **1.575E-08** | **0.843** | **0.076** | **7.750E-29** | **3.617E-28** |
| **GP5** | **-0.016** | **198** | **0.118** | **8.915E-01** | **8.915E-01** | **-0.383** | **101** | **0.161** | **1.419E-02** | **2.208E-02** | **-0.737** | **86** | **0.175** | **2.392E-05** | **4.185E-05** | **-0.281** | **0.084** | **7.965E-04** | **9.293E-04** |
| **GP6** | **0.044** | **198** | **0.115** | **6.950E-01** | **7.484E-01** | **0.368** | **101** | **0.164** | **2.054E-02** | **2.875E-02** | **0.512** | **86** | **0.162** | **1.279E-03** | **1.790E-03** | **0.243** | **0.082** | **2.947E-03** | **3.300E-03** |
| **GP7** | **-1.322** | **198** | **0.106** | **4.801E-28** | **6.722E-27** | **-0.891** | **101** | **0.166** | **1.429E-07** | **8.001E-07** | **-1.053** | **86** | **0.178** | **1.218E-08** | **5.504E-08** | **-1.168** | **0.080** | **1.080E-48** | **1.513E-47** |
| **GP8** | **0.770** | **198** | **0.122** | **7.336E-10** | **1.369E-09** | **0.669** | **101** | **0.167** | **5.725E-05** | **1.603E-04** | **0.158** | **86** | **0.190** | **3.818E-01** | **3.818E-01** | **0.613** | **0.087** | **2.499E-12** | **4.116E-12** |
| **GP9** | **-0.665** | **198** | **0.127** | **2.033E-07** | **2.995E-07** | **-1.031** | **101** | **0.168** | **3.383E-09** | **4.737E-08** | **-0.916** | **86** | **0.186** | **1.145E-06** | **2.466E-06** | **-0.825** | **0.089** | **1.500E-20** | **3.001E-20** |
| **GP10** | **-0.424** | **198** | **0.137** | **1.752E-03** | **2.133E-03** | **0.076** | **101** | **0.184** | **6.678E-01** | **6.678E-01** | **-0.295** | **86** | **0.202** | **1.259E-01** | **1.305E-01** | **-0.257** | **0.096** | **7.680E-03** | **8.271E-03** |
| **GP11** | **-0.593** | **198** | **0.127** | **3.040E-06** | **4.054E-06** | **-0.623** | **101** | **0.164** | **1.230E-04** | **2.870E-04** | **-0.444** | **86** | **0.176** | **9.281E-03** | **1.130E-02** | **-0.565** | **0.087** | **8.342E-11** | **1.298E-10** |
| **GP12** | **-1.393** | **198** | **0.099** | **7.437E-33** | **2.082E-31** | **-1.032** | **101** | **0.152** | **1.231E-10** | **3.446E-09** | **-1.228** | **86** | **0.166** | **8.773E-12** | **2.456E-10** | **-1.273** | **0.074** | **1.260E-65** | **3.528E-64** |
| **GP13** | **-1.023** | **198** | **0.116** | **1.525E-16** | **8.540E-16** | **-0.524** | **101** | **0.177** | **2.522E-03** | **4.154E-03** | **-0.975** | **86** | **0.187** | **3.250E-07** | **7.584E-07** | **-0.894** | **0.086** | **3.806E-25** | **1.522E-24** |
| **GP14** | **-0.766** | **198** | **0.098** | **1.042E-13** | **4.166E-13** | **-0.523** | **101** | **0.147** | **3.228E-04** | **6.026E-04** | **-0.810** | **86** | **0.146** | **7.059E-08** | **2.196E-07** | **-0.719** | **0.071** | **6.806E-24** | **2.382E-23** |
| **GP15** | **-0.878** | **198** | **0.113** | **1.267E-13** | **4.436E-13** | **-0.632** | **101** | **0.167** | **1.371E-04** | **2.953E-04** | **-0.892** | **86** | **0.167** | **1.807E-07** | **5.058E-07** | **-0.823** | **0.082** | **8.085E-24** | **2.515E-23** |
| **GP16** | **-0.866** | **198** | **0.125** | **2.099E-11** | **4.521E-11** | **-0.714** | **101** | **0.183** | **8.368E-05** | **2.130E-04** | **-1.049** | **86** | **0.178** | **1.376E-08** | **5.504E-08** | **-0.876** | **0.089** | **1.049E-22** | **2.938E-22** |
| **GP17** | **-0.918** | **198** | **0.125** | **1.547E-12** | **4.332E-12** | **-0.903** | **101** | **0.154** | **1.460E-08** | **1.022E-07** | **-1.105** | **86** | **0.178** | **2.812E-09** | **1.575E-08** | **-0.956** | **0.085** | **2.908E-29** | **1.628E-28** |
| **GP18** | **-0.752** | **198** | **0.104** | **2.731E-12** | **6.372E-12** | **-0.472** | **101** | **0.156** | **1.944E-03** | **3.401E-03** | **-0.823** | **86** | **0.155** | **2.218E-07** | **5.645E-07** | **-0.703** | **0.075** | **1.091E-20** | **2.350E-20** |
| **GP19** | **0.785** | **198** | **0.128** | **1.942E-09** | **3.399E-09** | **0.174** | **101** | **0.193** | **3.474E-01** | **3.602E-01** | **0.464** | **86** | **0.205** | **1.908E-02** | **2.226E-02** | **0.570** | **0.095** | **1.708E-09** | **2.392E-09** |
| **GP22** | **-0.733** | **198** | **0.129** | **2.470E-08** | **3.843E-08** | **-0.175** | **101** | **0.189** | **3.333E-01** | **3.589E-01** | **-0.720** | **86** | **0.198** | **2.283E-04** | **3.761E-04** | **-0.592** | **0.094** | **3.005E-10** | **4.428E-10** |
| **GP23** | **0.612** | **198** | **0.130** | **2.643E-06** | **3.701E-06** | **0.263** | **101** | **0.177** | **1.226E-01** | **1.430E-01** | **0.374** | **86** | **0.177** | **2.845E-02** | **3.187E-02** | **0.460** | **0.090** | **3.247E-07** | **4.133E-07** |
| **GP24** | **0.919** | **198** | **0.122** | **5.999E-13** | **1.866E-12** | **0.895** | **101** | **0.175** | **5.001E-07** | **2.334E-06** | **0.639** | **86** | **0.207** | **1.539E-03** | **2.052E-03** | **0.859** | **0.090** | **1.762E-21** | **4.110E-21** |
| **Derived IgG glycan traits** | | | | | | | | | | | | | | | | | | | |
|  | **FRA** | | | | | **ITA** | | | | | **UK** | | | | | **meta** | | | |
| **Glycan** | **effect** | **DF** | **SE** | **p.val** | **p.adj** | **effect** | **DF** | **SE** | **p.val** | **p.adj** | **effect** | **DF** | **SE** | **p.val** | **p.adj** | **effect** | **SE** | **p.val** | **p.adj** |
| **G0 total** | **0.708** | **198** | **0.103** | **2.900E-11** | **5.800E-11** | **0.616** | **101** | **0.150** | **3.892E-05** | **1.211E-04** | **0.876** | **86** | **0.155** | **4.255E-08** | **1.489E-07** | **0.724** | **0.074** | **2.517E-22** | **6.406E-22** |
| **G1 total** | **-0.034** | **198** | **0.133** | **7.911E-01** | **8.204E-01** | **-0.288** | **101** | **0.184** | **1.044E-01** | **1.271E-01** | **-0.625** | **86** | **0.194** | **1.012E-03** | **1.491E-03** | **-0.240** | **0.094** | **1.093E-02** | **1.134E-02** |
| **G2 total** | **-0.897** | **198** | **0.097** | **8.792E-18** | **6.154E-17** | **-0.607** | **101** | **0.147** | **3.457E-05** | **1.210E-04** | **-0.919** | **86** | **0.147** | **2.222E-09** | **1.575E-08** | **-0.834** | **0.071** | **7.375E-32** | **5.163E-31** |
| **S total** | **-0.424** | **198** | **0.119** | **3.506E-04** | **4.462E-04** | **-0.383** | **101** | **0.169** | **1.955E-02** | **2.875E-02** | **-0.726** | **86** | **0.170** | **1.905E-05** | **3.557E-05** | **-0.488** | **0.085** | **7.698E-09** | **1.026E-08** |
| **F total** | **1.126** | **198** | **0.117** | **5.820E-19** | **5.432E-18** | **0.960** | **101** | **0.160** | **7.031E-09** | **6.562E-08** | **1.136** | **86** | **0.177** | **9.812E-10** | **1.374E-08** | **1.083** | **0.083** | **1.357E-38** | **1.266E-37** |
| **B total** | **-0.065** | **198** | **0.131** | **6.127E-01** | **6.862E-01** | **0.300** | **101** | **0.173** | **7.122E-02** | **9.065E-02** | **0.278** | **86** | **0.186** | **1.189E-01** | **1.280E-01** | **0.119** | **0.091** | **1.909E-01** | **1.909E-01** |

**Supplementary Table 10: Comparison of directly measured IgG glycan traits and derived IgG glycan traits between persons with Down syndrome and their healthy siblings from Italian Down syndrome cohort.**

| **Directly measured IgG glycan traits** | | | | |
| --- | --- | --- | --- | --- |
| **Glycan** | **effect** | **SE** | **p.val** | **p.adj** |
| **GP1** | **0.003** | **0.200** | **2.376E-01** | **3.024E-01** |
| **GP2** | **-0.234** | **0.206** | **8.646E-01** | **8.646E-01** |
| **GP3** | **0.322** | **0.203** | **2.886E-01** | **3.367E-01** |
| **GP4** | **0.643** | **0.170** | **1.252E-03** | **5.010E-03** |
| **GP5** | **-0.321** | **0.221** | **3.424E-01** | **3.763E-01** |
| **GP6** | **0.602** | **0.234** | **2.088E-02** | **4.872E-02** |
| **GP7** | **-0.603** | **0.158** | **9.655E-04** | **4.506E-03** |
| **GP8** | **0.106** | **0.182** | **2.673E-01** | **3.254E-01** |
| **GP9** | **-1.052** | **0.225** | **4.748E-05** | **4.431E-04** |
| **GP10** | **0.466** | **0.247** | **1.143E-01** | **1.684E-01** |
| **GP11** | **-0.373** | **0.203** | **1.720E-01** | **2.409E-01** |
| **GP12** | **-0.853** | **0.139** | **5.165E-07** | **1.446E-05** |
| **GP13** | **-0.677** | **0.200** | **2.829E-03** | **8.801E-03** |
| **GP14** | **-0.417** | **0.192** | **7.988E-02** | **1.378E-01** |
| **GP15** | **-0.292** | **0.149** | **1.845E-01** | **2.459E-01** |
| **GP16** | **-0.897** | **0.196** | **1.941E-04** | **1.087E-03** |
| **GP17** | **-0.774** | **0.173** | **9.815E-05** | **6.870E-04** |
| **GP18** | **-0.502** | **0.188** | **2.432E-02** | **5.237E-02** |
| **GP19** | **0.433** | **0.220** | **1.068E-01** | **1.661E-01** |
| **GP22** | **-0.192** | **0.228** | **5.182E-01** | **5.374E-01** |
| **GP23** | **0.121** | **0.224** | **3.495E-01** | **3.763E-01** |
| **GP24** | **0.984** | **0.196** | **3.156E-05** | **4.418E-04** |
| **Derived IgG glycan traits** | | | | |
| **Glycan** | **effect** | **SE** | **p.val** | **p.adj** |
| **G0 total** | **0.614** | **0.186** | **4.664E-03** | **1.306E-02** |
| **G1 total** | **-0.479** | **0.222** | **6.922E-02** | **1.292E-01** |
| **G2 total** | **-0.486** | **0.185** | **2.661E-02** | **5.323E-02** |
| **S total** | **-0.433** | **0.203** | **8.365E-02** | **1.378E-01** |
| **F total** | **0.611** | **0.176** | **2.447E-03** | **8.564E-03** |
| **B total** | **0.702** | **0.247** | **9.500E-03** | **2.418E-02** |

**Supplementary Table 11. Comparison of derived IgG glycan traits between persons with Down syndrome (DS) without certain comorbidity and healthy controls from the general population.**

**Supplementary Table 12. Comparison of derived IgG glycan traits between persons with Down syndrome (DS) with and without a certain comorbidity.**

**Supplementary Table 13. Association of derived IgG glycan traits with age in controls and in persons with Down syndrome (DS).**

Analyses were performed by implementing Pearson correlation test. Cor = Pearson correlation

| **Controls** | | | | | | | | | |
| --- | --- | --- | --- | --- | --- | --- | --- | --- | --- |
|  | **FRA** | | **ITA** | | **UK** | | **meta** | | |
| **Glycan** | **Cor** | **p.val** | **Cor** | **p.val** | **Cor** | **p.val** | **effect** | **p.val** | **p.adj** |
| G0 total | 0.551 | **5.360E-10** | 0.441 | **9.414E-04** | 0.636 | **5.899E-06** | 0.554 | 9.545E-30 | **5.727E-29** |
| G1 total | -0.189 | **4.875E-02** | 0.124 | 3.771E-01 | -0.007 | 9.660E-01 | -0.074 | 2.820E-01 | 2.820E-01 |
| G2 total | -0.496 | **4.204E-08** | -0.445 | **8.322E-04** | -0.662 | **1.785E-06** | -0.540 | 3.305E-27 | **9.914E-27** |
| S total | -0.438 | **1.952E-06** | -0.373 | **5.931E-03** | -0.525 | **3.608E-04** | -0.445 | 3.375E-15 | **6.749E-15** |
| F total | -0.096 | 3.203E-01 | -0.148 | 2.918E-01 | -0.160 | 3.103E-01 | -0.123 | 7.654E-02 | 9.185E-02 |
| B total | 0.350 | **1.884E-04** | 0.377 | **5.444E-03** | 0.408 | **7.239E-03** | 0.370 | 1.215E-09 | **1.823E-09** |
| **Persons with Down syndrome** | | | | | | | | | |
|  | **FRA** | | **ITA** | | **UK** | | **meta** | | |
| **Glycan** | **Cor** | **p.val** | **Cor** | **p.val** | **Cor** | **p.val** | **effect** | **p.val** | **p.adj** |
| G0 total | 0.464 | **1.471E-06** | 0.518 | **3.715E-05** | 0.532 | **4.148E-05** | 0.498 | 2.073E-21 | **6.220E-21** |
| G1 total | -0.224 | **2.684E-02** | -0.191 | 1.544E-01 | -0.241 | 8.236E-02 | -0.219 | 9.607E-04 | **1.153E-03** |
| G2 total | -0.555 | **3.012E-09** | -0.526 | **2.675E-05** | -0.575 | **6.709E-06** | -0.553 | 3.711E-30 | **2.227E-29** |
| S total | -0.356 | **3.162E-04** | -0.438 | **6.490E-04** | -0.543 | **2.702E-05** | -0.440 | 4.632E-15 | **9.264E-15** |
| F total | -0.059 | 5.611E-01 | -0.216 | 1.072E-01 | -0.039 | 7.789E-01 | -0.100 | 1.459E-01 | 1.459E-01 |
| B total | 0.327 | **1.009E-03** | 0.440 | **6.110E-04** | 0.524 | **5.684E-05** | 0.422 | 1.424E-13 | **2.136E-13** |

**Supplementary Table 14. Comparison of the rate of change in the IgG glycan levels with age between persons with Down syndrome (DS) and healthy controls.**

|  | **FRA** | | | | | **ITA** | | | | | **UK** | | | | | **meta** | | | |
| --- | --- | --- | --- | --- | --- | --- | --- | --- | --- | --- | --- | --- | --- | --- | --- | --- | --- | --- | --- |
| **Glycan** | **effect** | **DF** | **SE** | **p.val** | **p.adj** | **effect** | **DF** | **SE** | **p.val** | **p.adj** | **effect** | **DF** | **SE** | **p.val** | **p.adj** | **effect** | **SE** | **p.val** | **p.adj** |
| G0 total | -0.002 | 202 | 0.013 | 0.870 | 0.870 | 0.008 | 105 | 0.013 | 0.546 | 0.931 | 0.010 | 90 | 0.011 | 0.378 | 0.454 | 0.006 | 0.007 | 0.438 | 0.526 |
| G1 total | -0.009 | 202 | 0.016 | 0.570 | 0.851 | -0.016 | 105 | 0.016 | 0.314 | 0.931 | -0.018 | 90 | 0.014 | 0.199 | 0.454 | -0.015 | 0.009 | 0.102 | 0.526 |
| G2 total | -0.007 | 202 | 0.012 | 0.544 | 0.851 | -0.006 | 105 | 0.013 | 0.647 | 0.931 | -0.004 | 90 | 0.011 | 0.709 | 0.709 | -0.006 | 0.007 | 0.421 | 0.526 |
| S total | 0.016 | 202 | 0.014 | 0.267 | 0.851 | -0.008 | 105 | 0.014 | 0.586 | 0.931 | -0.012 | 90 | 0.012 | 0.308 | 0.454 | -0.003 | 0.008 | 0.743 | 0.743 |
| F total | 0.005 | 202 | 0.014 | 0.709 | 0.851 | 0.001 | 105 | 0.014 | 0.931 | 0.931 | 0.013 | 90 | 0.013 | 0.282 | 0.454 | 0.007 | 0.008 | 0.370 | 0.526 |
| B total | 0.006 | 202 | 0.015 | 0.705 | 0.851 | -0.003 | 105 | 0.015 | 0.853 | 0.931 | 0.017 | 90 | 0.013 | 0.198 | 0.454 | 0.007 | 0.008 | 0.374 | 0.526 |

**Supplementary Table 15. Comparison of derived IgG glycan traits between around 4-year-old children with Down syndrome (DS) from the UK DS cohort and 4-year-old healthy children.**

| **Glycan** | **Cohort** | **effect** | **DF** | **SE** | **p.val** | **p.adj** |
| --- | --- | --- | --- | --- | --- | --- |
| G0 total | UK | 1.446 | 15 | 0.551 | 0.007 | **0.044** |
| G1 total | UK | -0.520 | 15 | 0.651 | 0.374 | 0.463 |
| G2 total | UK | -1.241 | 15 | 0.543 | 0.017 | 0.052 |
| S total | UK | -1.195 | 15 | 0.631 | 0.043 | 0.087 |
| F total | UK | 0.537 | 15 | 0.690 | 0.386 | 0.463 |
| B total | UK | 0.279 | 15 | 0.584 | 0.592 | 0.592 |
