# Supplementary material for: Dose imbalance of DYRK1A kinase causes systemic progeroid status in Down syndrome by increasing the un-repaired DNA damage and reducing LaminB1 levels
Source: eBioMedicine. 2023 Jul 12;94:104692. doi: 10.1016/j.ebiom.2023.104692 (PMC10435767; doi:10.1016/j.ebiom.2023.104692)
Supplement: Supplementary Discussion [file mmc2.docx]

**Supplementary Discussion**

**Whole plasma proteome glycosylation in DS**

An earlier study of the whole blood plasma protein glycosylation found differences in 76 individuals with DS from a single population compared to euploid controls(1), indicating that glycosylation changes associated with ageing of uncharacterised plasma proteins appeared earlier in DS, but were only partially overlapping with ageing markers of euploid persons, revealing a DS-specific ageing phenotype(1). However, this study investigated changes in glycans that originate from total plasma proteins, and not from one specific protein such as IgG, thus, in addition to changes in glycosylation, the observed differences also probably reflected changes in the levels of individual plasma proteins. Our data allow us to calculate a chronological age estimate, which based on DS values for G2 and G0 corresponded on average to 19 years older euploid individuals (range 11.7-31.1 years).

**Additional discussion on glycans with core fucose in DS**

Our study also reveals the increase in fucosylated (F) structures of IgG, which appears to be a DS-specific trait. Increase in IgG glycans with core fucose has rarely been reported in other diseases, and these reports include rheumatoid arthritis, ANCA-associated vasculitis, Chron’s disease, visceral leishmaniasis, HIV infection, hepatocellular carcinoma, galactosaemia and hypertension(2). The presence of core fucose is known to decrease the binding of IgG to activating FcγRIIIA and FcγRIIIB receptors up to 100-fold, dampening the downstream antibody-dependent cellular cytotoxicity (ADCC)(2, 3). Increased core fucosylation that we reveal here as a DS-specific phenotype could therefore explain the mechanism causing the decreased ADCC ability observed in DS lymphocytes(4), which may contribute to a higher frequency, severity, duration and mortality of infections, in particular those of the upper respiratory tract, observed in DS(5, 6).

**Additional notes on IgG glycan profiles for DS with and without co-morbidities**

Persons with DS also show a much higher incidence of a whole spectrum of auto-immune diseases, in particular thyroid conditions and type-1-diabetes(7, 8), while the most frequent co-morbidity and cause of death in DS remains Alzheimer’s disease (AD)(9). In the general population, it has been reported that several immune system disorders, a whole spectrum of auto-immune diseases and Alzheimer’s disease (AD), are all among the diseases exhibiting an altered plasma IgG glycosylation profile in the general population(2). We therefore wanted to examine if the differences between DS and euploid controls we observed for the IgG glycosylation profiles were merely a secondary effect of the much higher prevalence of these co-morbidities in the DS population. We therefore repeated the analysis for DS without specific co-morbidities (thyroid dysfunction + any other autoimmune conditions, frequent infections, or AD-dementia), against the control population. We found nearly identical IgG glycan differences as for the whole (co-morbidity-unfiltered) DS cohorts, proving these alterations are caused by trisomy 21 as a genetic condition, and not as a secondary effect of DS co-morbidities. Corroborating to the observations in the general population, we did find that persons with DS with diagnosed autoimmunity had a significantly lower level of G2 IgG glycans (**Figure 1** and **Supplementary Table 12**) than persons with DS without this diagnosis. Also, comparison of DS with and without autoimmune thyroid disease, the most common autoimmune disease associated with DS, revealed a higher level of G0 IgG glycans, and lower levels of G2 and S IgG glycans in persons with DS diagnosed with thyroid disease (**Figure 1** and **Supplementary Table 12**).

We observed no significant differences in IgG glycosylation patterns between DS with and without dementia, nor between DS with and without frequent infections (**Supplementary Figure 4**, **Supplementary Figure 5** and **Supplementary Table 12).** The main limitation of our study is in the relatively small sub-population of DS patients with specific co-morbidities. This potentially prevents the detection of correlations of IgG glycan profiles with certain diseases within the DS cohorts. This is particularly important for AD-dementia correlations. People with DS have a similar curve of positive correlation of the incidence of dementia with age as euploids, but at younger age of onset and with a much increased frequency(10). As IgG glycan profiles also change with age in both DS with and without dementia, any additional profile-skewing correlating with dementia is difficult to separate from the effect of age alone, in the sample size we studied. A larger study of older adults with DS would be required to tease out these differences, with uniformly applied criteria for the dementia diagnosis, and with sufficient numbers of those with and without dementia.

**References for Extended Online Methods**

1. Borelli V, Vanhooren V, Lonardi E, Reiding KR, Capri M, Libert C, et al. Plasma N-Glycome Signature of Down Syndrome. Journal of proteome research. 2015;14(10):4232-45.

2. Gudelj I, Lauc G, Pezer M. Immunoglobulin G glycosylation in aging and diseases. Cellular Immunology. 2018;333(January):65-79.

3. Shields RL, Lai J, Keck R, O'Connell LY, Hong K, Meng YG, et al. Lack of fucose on human IgG1 N-linked oligosaccharide improves binding to human Fcgamma RIII and antibody-dependent cellular toxicity. The Journal of biological chemistry. 2002;277(30):26733-40.

4. Warren RP, Healey MC, Johnston AV, Sidwell RW, Radov LA, Murray RJ, et al. PR 879-317A enhances in vitro immune activity of peripheral blood mononuclear cells from patients with Down syndrome. Int J Immunopharmacol. 1987;9(8):919-26.

5. Chicoine B, Rivelli A, Fitzpatrick V, Chicoine L, Jia G, Rzhetsky A. Prevalence of Common Disease Conditions in a Large Cohort of Individuals With Down Syndrome in the United States. J Patient Cent Res Rev. 2021;8(2):86-97.

6. Ram G, Chinen J. Infections and immunodeficiency in Down syndrome. Clin Exp Immunol. 2011;164(1):9-16.

7. Ferrari M, Stagi S. Autoimmunity and Genetic Syndromes: A Focus on Down Syndrome. Genes (Basel). 2021;12(2).

8. Gimenez-Barcons M, Casteras A, Armengol Mdel P, Porta E, Correa PA, Marin A, et al. Autoimmune predisposition in Down syndrome may result from a partial central tolerance failure due to insufficient intrathymic expression of AIRE and peripheral antigens. J Immunol. 2014;193(8):3872-9.

9. Hithersay R, Startin CM, Hamburg S, Mok KY, Hardy J, Fisher EMC, et al. Association of Dementia With Mortality Among Adults With Down Syndrome Older Than 35 Years. JAMA Neurol. 2018.

10. Startin CM, D'Souza H, Ball G, Hamburg S, Hithersay R, Hughes KMO, et al. Health comorbidities and cognitive abilities across the lifespan in Down syndrome. J Neurodev Disord. 2020;12(1):4.
