# Supplementary material for: Dose imbalance of DYRK1A kinase causes systemic progeroid status in Down syndrome by increasing the un-repaired DNA damage and reducing LaminB1 levels
Source: eBioMedicine. 2023 Jul 12;94:104692. doi: 10.1016/j.ebiom.2023.104692 (PMC10435767; doi:10.1016/j.ebiom.2023.104692)
Supplement: Caption for Supplementary Material [file mmc3.docx]

**Captions for Supplementary Material – Supplementary Figure Legends and Supplementary Table Captions**

**Supplementary Figures Legends**

**Supplementary Figure 1. Chromatographic profile of N-glycans released from immunoglobulin G (IgG) isolated from human plasma**. IgG glycans are separated into 24 peaks labelled GP1-GP24. Majority of individual peaks correspond to a single glycan structure (composition indicated by the colour code as shown in the lower panel). In case of multiple glycan structures per glycan peak, the uppermost one is the most abundant structure in the corresponding peak and the lower ones are minor glycan structures (GP – glycan peak). Top row defines the derived traits used in the rest of the manuscript: G0 – IgG glycans without galactose, G1 – IgG glycans with one galactose, G2 – IgG glycans with two galactoses, S – IgG glycans with sialic acid(s).

**Supplementary Figure 2. Relative abundances of directly measured IgG glycan traits (GP1-GP24) in persons with Down syndrome (DS) and in healthy controls shown separately for three cohorts of adults with DS from France (FRA), Italy (ITA) and United Kingdom (UK).** Data are shown as box plots. Each box represents the 25^th^ to 75^th^ percentiles (the interquartile range (IQR)). Lines inside the boxes represent the median. Lines outside the boxes indicate data within 1.5 x IQR from the 25^th^ and 75^th^ percentiles. Black dots indicate outliers. Glycan structures corresponding to individual glycan peaks (GP1-24) are provided in Figure S1. Asterisk * indicates statistically significant differences (p <0.05, meta-analysis) between DS individuals and healthy controls (additional information is available in **Supplementary Table 8**).

**Supplementary Figure 3. Comparison of levels of derived IgG glycan traits between healthy control individuals, persons with DS without frequent infections and persons with DS with frequent infections.** FRA – DS cohort from France, ITA – DS cohort from Italy, UK – DS cohort from the UK. G0 total – sum of IgG glycans without galactose, G1 total – sum of IgG glycans with one galactose, G2 total – sum of IgG glycans with two galactoses, S total – sum of IgG glycans with sialic acid(s), F total – sum of IgG glycans with core fucose, B total – sum of IgG glycans with bisecting GlcNAc. Data are shown as box plots. Each box represents the 25^th^ to 75^th^ percentiles (IQR). Lines inside the boxes represent the median. Lines outside the boxes indicate data within 1.5 x IQR from the 25^th^ and 75^th^ percentiles. Black dots indicate outliers. No significant difference was observed between persons with DS persons with or without frequent infections (additional information is available in **Supplementary Tables 11** and **12)**.

**Supplementary Figure 4. Relative abundances of directly measured IgG glycan traits (GP1-GP24) in persons with DS and in their healthy siblings (DSS) from the Italian cohort of DS.** Each dot represents a single individual. Lines connect the dots of each sibling pair. The age of each individual in years is shown on the x-axis. Asterisk * indicates statistically significant differences (p <0.05, meta-analysis) between DS individuals and their healthy siblings and asterisk in brackets (*) indicates nominal significance (p-value slightly above 0.05) (additional information is available in **Supplementary Table 9**).

**Supplementary Figure 5. Levels of derived IgG glycan traits in persons with DS and in their healthy siblings (DSS) from the Italian cohort of DS.** Each dot represents a single individual. Lines connect the dots of each sibling pair. The age of each individual in years is shown on the x-axis. G0 total – sum of IgG glycans without galactose, G1 total – sum of IgG glycans with one galactose, G2 total – sum of IgG glycans with two galactoses, S total – sum of IgG glycans with sialic acid(s), F total – sum of IgG glycans with core fucose, B total – sum of IgG glycans with bisecting GlcNAc. Asterisk * indicates statistically significant differences (p <0.05, meta-analysis) between DS individuals and their healthy siblings and asterisk in brackets (*) indicates nominal significance (p-value slightly above 0.05) (additional information is available in **Supplementary Table 9**).

**Supplementary Figure 6: Generation and characterisation of T-CRO1 iPSC and its CRISPR/Cas9 edited isogenic derivates D-CRO1-Δ1 and D-CRO1-Δ5.**

**a:** Immunostaining with OCT4, TRA-1-60, TRA-1-81 and SSEA4 along with Alkaline Phosphatase staining and morphology of colonies confirm the successful reprogramming of T-CRO1 iPSCs from PBMCs.

**b:** Embryoid bodies generated from T-CRO1 iPSCs and allowed to spontaneously differentiate. Immunostaining for SMA, Nestin, FOXA2 and Brachyury confirm the differentiation of endoderm, mesoderm and ectoderm, demonstrating the pluripotency of T-CRO1 iPSCs.

**c:** Graphs displaying TIDE analysis of the PCR amplified *DYRK1A* target site in CRO1 iPSCs. Unedited T-CRO1 shows close to 100% wild-type sequence. In both CRO1Δ1 and CRO1Δ5 the mutation to WT ratio is 1:1 indicating total loss of one of the three copies of *DYRK1A*.

**d:** Schematic representation of the chromosomal structure for chr21 in T-CRO1 (unedited) iPSCs and its D-CRO1 (CRISPR/Cas9-edited) isogenic iPSC clones, in which genome editing resulted in excision of the entire duplicated region.

**e:** SNP array analysis of genomic DNA from untargeted parental T-CRO1 and the *DYRK1A* CRISPR/Cas9 targeted D-CRO1-Δ1 and D-CRO1-Δ5 iPSCs. The B-allele frequency and Log R ratio of chr21 (left) confirms excision of the 4.083 Mb duplication that is present in the parental T-CRO1 cell line. Excision was also confirmed by copy number variant analysis (right) as the blue band indicating a copy number of 3 has been eliminated on Chr21 D-CRO1-Δ1 and D-CRO1-Δ5.

**f:** A portion of the DNA sequence from *DYRK1A* exon 7 is shown. In the WT sequence (top), the gRNA is underlined, and the PAM is shown in bold. The two CRISPR/Cas9 edited cell lines (D-CRO1-Δ1 and D-CRO1-Δ5) are shown below WT and their respective 1 and 5 bp deletions indicated by red dashes.

**g:** *In silico* analysis of *DYRK1A* DNA translation reveals that the 5 bp and 1 bp deletions in exon 7 (**underlined**) cause the appearance of some amino acids (red) within the kinase domain followed by stop codons leading to premature termination

**Supplementary Figure 7: The effect of DYRK1A dose imbalance on DNA repair pathways in iPSCs.**

**a-b:** T-CRO1 iPSCs were treated with DMSO or a DYRK1A inhibitor (300 nM harmine or 500 nM ID8) for 12 hours before fixation, alongside untreated T-CRO1, D-CRO1-Δ1 and D-CRO1-Δ5. iPSCs were stained for γH2AX or γH2AX and 53BP1 then imaged by confocal microscopy. Sufficient images were taken from two independent experiments consisting of three wells each to ensure that a minimum of 2,000 nuclei were counted for each staining condition and each cell line. Data are shown relative to T-CRO1 in which the number of puncta per nucleus was set to 1. Each dot on the histogram represents the data of an individual image: (**a**) γH2AX and (**b**) 53BP1. Quantification of foci was performed automatically using IMARIS. Statistical significance was calculated by one-way ANOVA followed by Dunnett’s correction for multiple comparisons. Error bars: SEM.

**c:** Colocalisation of γH2AX and 53BP1 foci was determined by IMARIS software. Statistical significance was calculated by one-way ANOVA followed by Dunnett’s correction for multiple comparisons. Error bars: SEM.

For all parts: **p <0.01, *** p <0.001, ****p <0.0001, ns= not significant.

**Supplementary Figure 8: Validation of T21 *DYRK1A*-edited iPSC clones**

**a:** A schematic representation of the protein domain structure of human DYRK1A (Top) adapted from [Tejedor FJ. 2018. Dyrk1a. In Encyclopedia of Signaling Molecules, doi:10.1007/978-3 -319-

67199-4_101613]. The region in which a premature stop codon would prevent further translation is indicated below the full-length protein. Exon 7, which was targeted by CRISPR/Cas9, falls within the kinase domain. **WD**: binding motif to adaptor protein WDR68 (DCAF7), **NLS**: Nuclear Localisation Signal, **DH**: DYRK Homology region, **PEST**: Proline-glutamic acid-serine-rich domain, **HIS**: consecutive histidine residues, **S/T**: serine/threonine rich domain.

**b:** *DYRK1A* exon 7 sequence indicating the gRNA (underlined) and the PAM site (bold). For each of the CRISPR/Cas9 edited iPSC lines, deletions are indicated by red dashes and insertions are indicated by red asterisks. T21-0xDYRK has a mutation on all three alleles and T21-1xDYRK has two mutated and one normal allele.

**c:** TIDE analysis of the PCR amplified *DYRK1A* exon 7 target site in T21C5 *DYRK1A-*targeted iPSCs. Graphs indicate the percentage of sequence corresponding to each mutation. Each mutation accounts for approximately one third of the total sequence trace, which is expected in trisomic cells. The corresponding HSA21 SNP array below confirms T21 was maintained following CRISPR-Cas9 editing.

**d:** Representative confocal images of the parental T21C5 and the CRISPR/Cas9 edited (T21-1xDYRK and T21-0xDYRK) iPSCs immunostained for markers of pluripotency. TRA 1-60, TRA 1-81 and SSEA4 are localised to the cell surface, and OCT4 is nuclear. Scale bars represent 20 µm.

**e:** Western blot of iPSC lysates confirm lack of DYRK1A protein expression (indicated with an arrow) in the T21-0xDYRK clone when compared to the parental T21 clone.

**f:** qRT-PCR confirms a 61% reduction in *DYRK1A* transcript levels in the T21-0xDYRK iSPC clone. [Some transcript was detected, however this will be subject to nonsense-mediated mRNA decay and not translated into functional protein]. The average of each of three independent experiments is shown. Error bars: SEM. **** p <0.0001 (unpaired two-tailed Student’s t-test).

**g:** For comparison, qRT-PCR confirms the extent to which *DYRK1A* transcript levels are increased in the unedited T21 iPSC clones compared to their isogenic D21 clones set to 1. The different symbols in the histograms for D21 and T21 represent the 3 isogenic clones used per genotype (same clones as in Figure 4A-D). Error bars: SD. *** p <0.001 (unpaired two-tailed Student’s t-test).

**Supplementary Figure 9: Characterisation of T-CRO1, D-CRO1-Δ1 and D-CRO1-Δ5 Cerebral Organoids at DIV63**

Representative confocal images of histological sections of **a:** T-CRO1, **b:** D-CRO1-Δ1 and **c:** D-CRO1-Δ5 organoids labelled with a variety of neuronal and astrocytic markers which demonstrate formation of mature neurons expressing a range of cortical layer markers appropriate for the organoid age in vitro and showing the absence of any detectable astrocytes. Nuclei are stained with DAPI. MAP2 (neuronal dendrites and perikarya), 3R-Tau (neurons), Reelin (Layer I), SATB2 (Layer III), TBR1 (Layer IV) and BRN2 (Layer VI), GFAP (astrocytes). Scale bars, 50 μm.

**Supplementary Figure 10: DYRK1A expression in DIV63 Cerebral Organoids**

**a:** Representative confocal images of histological sections from DIV63 T-CRO1, D-CRO1-Δ1 and D-CRO1-Δ5 organoids labelled with a N-terminal DYRK1A antibody. Neurons are labelled with MAP2. Scale bars, 10 μm.

**b:** Quantification of DYRK1A expression using the N-terminal antibody and normalised to MAP2. Lower expression was detected in D-CRO1-Δ1 and D-CRO1-Δ5 sections compared to T-CRO1.

**c:** Representative confocal images of histological sections from DIV63 T-CRO1, D-CRO1-Δ1 and D-CRO1-Δ5 organoids labelled with a C-terminal DYRK1A antibody. Neurons are labelled with MAP2. Scale bars, 10 μm.

**d:** Quantification of DYRK1A expression using the C-terminal antibody and normalised to MAP2. DYRK1A expression was significantly lower in D-CRO1-Δ1 and D-CRO1-Δ5 compared to T-CRO1.

In all cases, enough images were taken to capture n>20,000 nuclei from 3 different organoids per genotype. Each dot represents one image. Error bars: SEM. Statistics were calculated by one-way ANOVA followed by Tukey’s correction for multiple comparisons (ns p > 0.05, *** p < 0.001, **** p < 0.0001).

**Supplementary Figure 11: γH2AX and Lamin B1 expression by immunofluorescence in D21, T21 and T21-1xDYRK organoids at DIV30, and D21 and T21 organoids at DIV50**

**a:** Immunofluorescence images of organoid sections after 30 days of differentiation showing MAP2, Lamin B1 and γH2AX expression in D21 organoids alongside T21 and T21-1xDYRK organoids. Scale bar 20 μm.

**b:** T21 cerebral organoids showed increased γH2AX puncta per nucleus compared to D21 control organoids at DIV30. Reduction of DYRK1A dose by CRISPR/Cas9 in the T21-1xDYRK organoids resulted in reduced γH2AX puncta per nucleus compared to the T21 organoids, to almost the levels observed in D21 organoids at DIV30.

**c:** T21 cerebral organoids showed decreased relative expression of Lamin B1 compared to D21 control organoids at DIV30. Reduction of DYRK1A dose by CRISPR/Cas9 in the T21-1xDYRK organoids resulted in increased Lamin B1 expression compared to the T21 organoids, to similar levels observed in D21 organoids at DIV30. Lamin B1 expression was separately normalised to either MAP2 or DAPI, to account for the fact that neurons in DIV30 organoids may not yet all express MAP2.

**d:** T21 cerebral organoids showed increased γH2AX puncta per nucleus compared to D21 control organoids at DIV50. T21-1xDYRK organoids did not survive sufficiently to allow quantification at this timepoint.

**e:** T21 cerebral organoids showed increased relative expression of Lamin B1 compared to D21 control organoids at DIV50. T21-1xDYRK organoids did not survive sufficiently to allow quantification at this timepoint.

For b-e, 2-3 organoids per genotype or condition were analysed. For DIV30 6 images, and for DIV50 8-16 images, containing a total of n>1,000 nuclei per cell line were analysed and graphs show mean ± SEM. Statistics were calculated by one-way ANOVA followed by Dunnett's correction for multiple comparisons.

**Supplementary Tables Captions and Legends**

**Supplementary Table 1. Human Foetal Fibroblasts**

**Supplementary Table 2. Primer sequences**

**Supplementary Table 3: Primers used for qPCR**

**Supplementary Table 4. Primary antibodies**

**Supplementary Table 5. Secondary antibodies**

**Supplementary Table 6: Primary human tissues**

**Supplementary Table 7. Chemicals**

**Supplementary Table 8. Sex-disaggregated data on characteristics of Down syndrome cohorts and healthy controls.** Sex-disaggregated data of cohorts presented in Table 1.

**Supplementary Table 9. Comparison of directly measured IgG glycan traits and derived IgG glycan traits between persons with Down syndrome (DS) and healthy controls from the general population.** FRA – DS cohort from France, ITA – DS cohort from Italy, UK – DS cohort from United Kingdom (UK), meta – meta-analysis. G0 total – sum of IgG glycans without galactose, G1 total – sum of IgG glycans with one galactose, G2 total – sum of IgG glycans with two galactoses, S total – sum of IgG glycans with sialic acid(s), F total – sum of IgG glycans with core fucose, B total – sum of IgG glycans with bisecting GlcNAc (see Figure S1 for definition of the traits). The statistical significance of the differences between the DS group and the control group was assessed using a general linear model. Glycan data were adjusted for age and sex. False discovery rate was controlled using the Benjamini–Hochberg method. Effect sizes are expressed in standard deviation units. Statistically significant findings (p.adj <0.05) are shown in bold. The rows shaded gray indicate glycans that differed significantly between persons with DS and controls in all three examined cohorts.

**Supplementary Table 10. Comparison of directly measured IgG glycan traits and derived IgG glycan traits between persons with DS and their healthy siblings from the Italian cohort.** G0 total – sum of IgG glycans without galactose, G1 total – sum of IgG glycans with one galactose, G2 total – sum of IgG glycans with two galactoses, S total – sum of IgG glycans with sialic acid(s), F total – sum of IgG glycans with core fucose, B total – sum of IgG glycans with bisecting GlcNAc. The significance of the differences between the affected and unaffected siblings was assessed using a general linear model. Glycan data were adjusted for age and sex. False discovery rate was controlled using the Benjamini–Hochberg method. Effect sizes are expressed in standard deviation units. Statistically significant findings (p.adj <0.05) are shown in bold.

**Supplementary Table 11. Comparison of derived IgG glycan traits between persons with DS without certain comorbidity and healthy controls from the general population.** FRA – DS cohort from France, ITA – DS cohort from Italy, UK – DS cohort from the UK, meta – meta-analysis. G0 total – sum of IgG glycans without galactose, G1 total – sum of IgG glycans with one galactose, G2 total – sum of IgG glycans with two galactoses, S total – sum of IgG glycans with sialic acid(s), F total – sum of IgG glycans with core fucose, B total – sum of IgG glycans with bisecting GlcNAc. Comorbidities under study were: autoimmune disease, dementia, frequent infections, thyroid disease. The significance of the differences between the DS group and the control group was assessed using general linear model. Glycan data were adjusted for age and sex. False discovery rate was controlled using Benjamini–Hochberg method. Effect sizes are expressed in standard deviation units. Statistically significant findings (p <0.05) are shown in bold.

**Supplementary Table 12. Comparison of derived IgG glycan traits between persons with DS with and without a certain comorbidity.** FRA – DS cohort from France, ITA – DS cohort from Italy, UK – DS cohort from the UK, meta – meta-analysis. G0 total – sum of IgG glycans without galactose, G1 total – sum of IgG glycans with one galactose, G2 total – sum of IgG glycans with two galactoses, S total – sum of IgG glycans with sialic acid(s), F total – sum of IgG glycans with core fucose, B total – sum of IgG glycans with bisecting GlcNAc. Comorbidities under study were: autoimmune disease, dementia, frequent infections, thyroid disease. The significance of the differences between the two DS groups (with and without certain comorbidity) was assessed using general linear model. Glycan data were adjusted for age and sex. False discovery rate was controlled using the Benjamini–Hochberg method. Effect sizes are expressed in standard deviation units. Statistically significant findings (p <0.05) are shown in bold. NA = data not available.

**Supplementary Table 13. Association of derived IgG glycan traits with age in controls and in persons with Down syndrome (DS).** FRA – DS cohort from France, ITA – DS cohort from Italy, UK – DS cohort from the UK, meta – meta-analysis. G0 total – sum of IgG glycans without galactose, G1 total – sum of IgG glycans with one galactose, G2 total – sum of IgG glycans with two galactoses, S total – sum of IgG glycans with sialic acid(s), F total – sum of IgG glycans with core fucose, B total – sum of IgG glycans with bisecting GlcNAc. Statistically significant associations (p <0.05) are shown in bold. Cor = correlation coefficient. False discovery rate was controlled using the Benjamini–Hochberg method. Data yielding a p-value of less than 0.05 was considered statistically significant. Effect sizes are expressed in standard deviation units. No significant difference was observed in the rate of change in IgG glycan levels with age between controls and persons with DS.

**Supplementary Table 14. Comparison of the rate of change in the IgG glycan levels with age between persons with Down syndrome (DS) and healthy controls.** FRA – DS cohort from France, ITA – DS cohort from Italy, UK – DS cohort from the UK, meta – meta-analysis. G0 total – sum of IgG glycans without galactose, G1 total – sum of IgG glycans with one galactose, G2 total – sum of IgG glycans with two galactoses, S total – sum of IgG glycans with sialic acid(s), F total – sum of IgG glycans with core fucose, B total – sum of IgG glycans with bisecting GlcNAc. False discovery rate was controlled using the Benjamini–Hochberg method. Data yielding a p-value of less than 0.05 was considered statistically significant. Effect sizes are expressed in standard deviation units. No significant difference was observed in the rate of change in IgG glycan levels with age between controls and persons with DS.

**Supplementary Table 15. Comparison of derived IgG glycan traits between around 4-year-old children with Down syndrome (DS) from the UK DS cohort and 4-year-old healthy children.** G0 total – sum of IgG glycans without galactose, G1 total – sum of IgG glycans with one galactose, G2 total – sum of IgG glycans with two galactoses, S total – sum of IgG glycans with sialic acid(s), F total – sum of IgG glycans with core fucose, B total – sum of IgG glycans with bisecting GlcNAc. The significance of the differences between children with DS and healthy children was assessed using general linear model. Glycan data were adjusted for age and sex. False discovery rate was controlled using the Benjamini–Hochberg method. Effect sizes are expressed in standard deviation units. Statistically significant findings (p <0.05) are shown in bold.
