# Supplementary material for: Dose imbalance of DYRK1A kinase causes systemic progeroid status in Down syndrome by increasing the un-repaired DNA damage and reducing LaminB1 levels
Source: eBioMedicine. 2023 Jul 12;94:104692. doi: 10.1016/j.ebiom.2023.104692 (PMC10435767; doi:10.1016/j.ebiom.2023.104692)
Supplement: Supplementary Western Blots [file mmc4.pptx]

## Slide 1
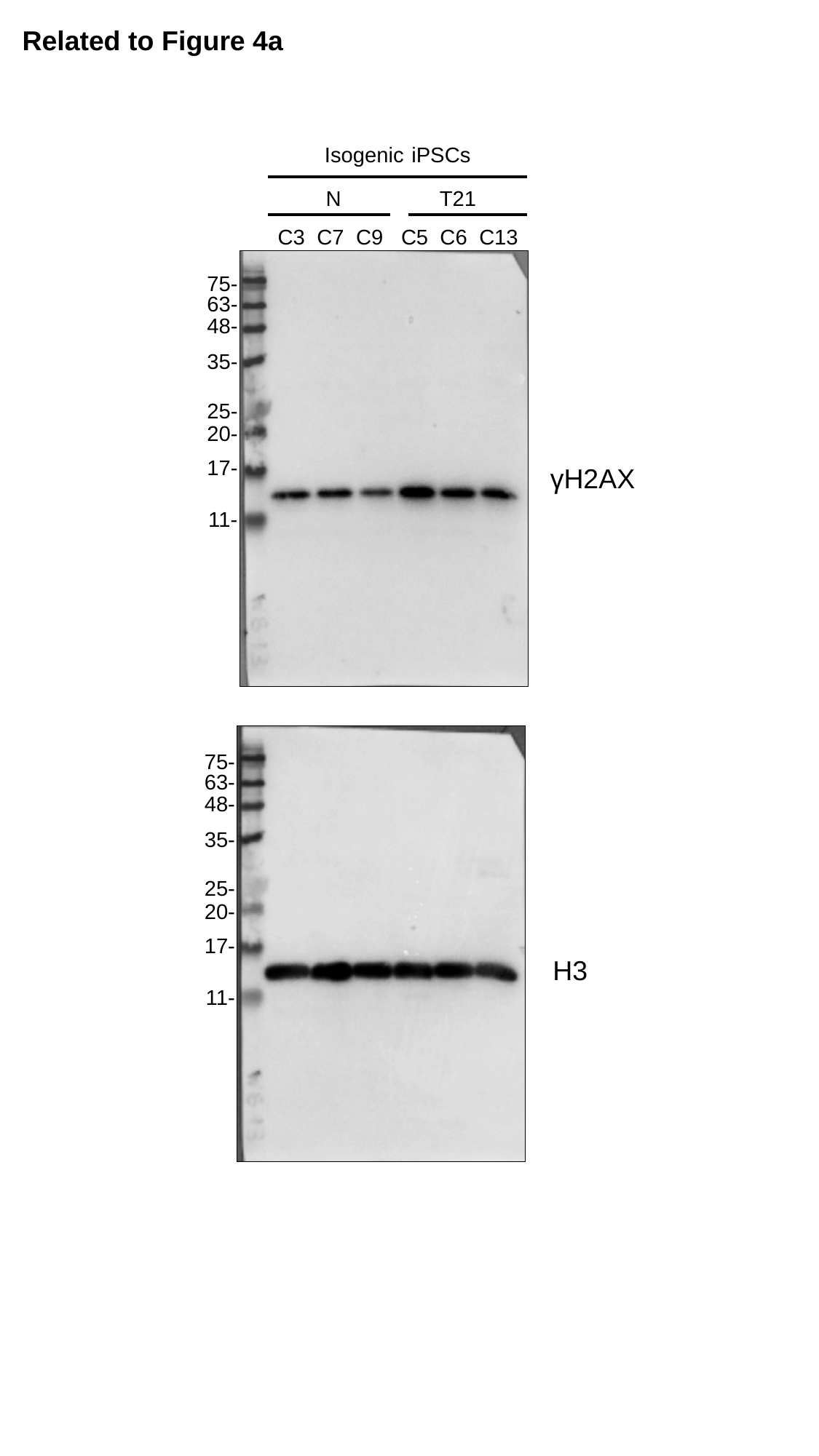

Related to Figure 4a
Isogenic iPSCs
N
T21
C3 C7 C9 C5 C6 C13
75-
63-
48-
35-
25-
20-
17-
11-
γH2AX
H3
75-
63-
48-
35-
25-
20-
17-
11-

## Slide 2
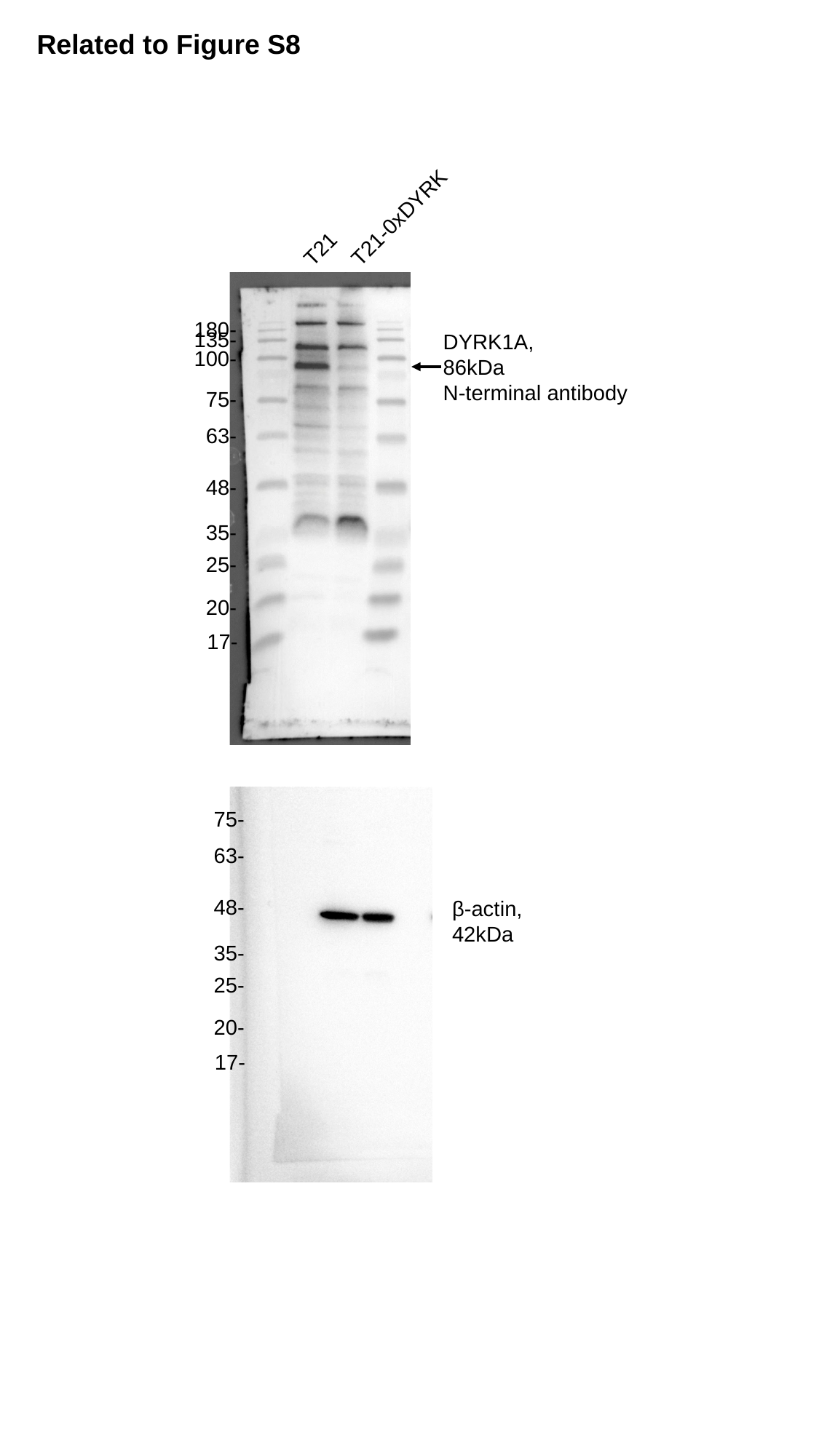

Related to Figure S8
T21-0xDYRK
T21
180-
135-
100-
75-
63-
48-
35-
25-
20-
17-
DYRK1A,
86kDa
N-terminal antibody
75-
63-
48-
35-
25-
20-
17-
β-actin,
42kDa

## Slide 3
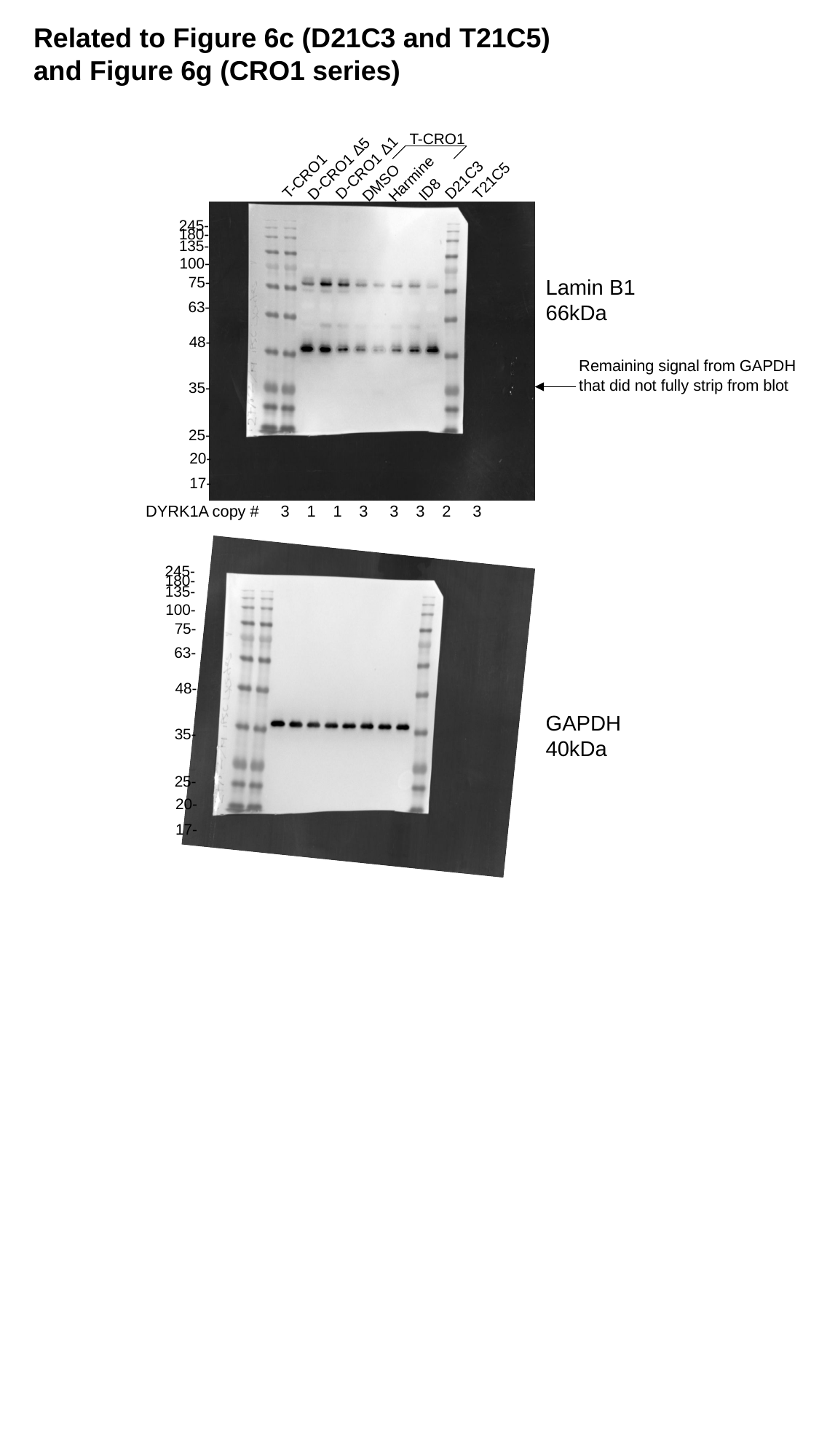

Related to Figure 6c (D21C3 and T21C5)
and Figure 6g (CRO1 series)
T-CRO1
T-CRO1
D-CRO1 Δ1
D-CRO1 Δ5
Harmine
D21C3
DMSO
T21C5
ID8
245-
180-
135-
100-
75-
63-
48-
35-
25-
20-
17-
Lamin B1
66kDa
Remaining signal from GAPDH that did not fully strip from blot
DYRK1A copy # 3 1 1 3 3 3 2 3
245-
180-
135-
100-
75-
63-
48-
35-
25-
20-
17-
GAPDH
40kDa

## Slide 4
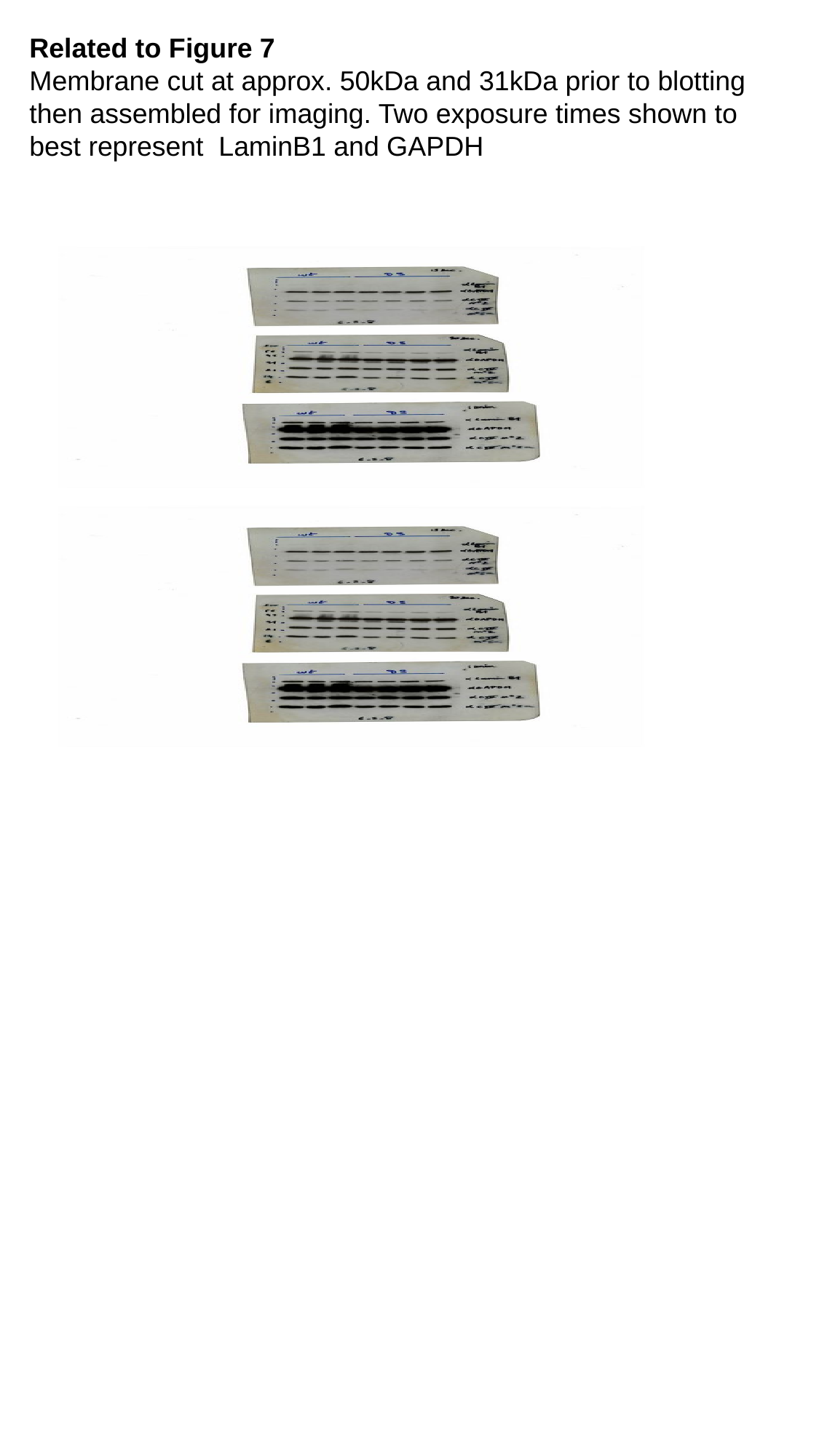

Related to Figure 7
Membrane cut at approx. 50kDa and 31kDa prior to blotting then assembled for imaging. Two exposure times shown to best represent LaminB1 and GAPDH
